# Proceedings of the 2018 Childhood Arthritis and Rheumatology Research Alliance (CARRA) Scientific Meeting: Denver, CO, USA. 12-15 April 2018

**DOI:** 10.1186/s12969-018-0252-y

**Published:** 2018-07-09

## O1 Identifying juvenile scleroderma immunophenotype subsets

### Kathryn Torok^1^, Suzanne Li^2^, Anne Stevens^3^, Marvin Fritzler^4^, Laura Schanberg^5^, Kelly Mieszkalski^5^, Anne Dennos^5^, Sarah Ringold^3^, Marc Natter^6^, Min-Lee Chang^6^, Emily Mirizio^1^, Kaila Schollaert-Fitch^1^, for the CARRA scleroderma subcommittee

#### ^1^University of Pittsburgh, Pittsburgh, PA, USA; ^2^Hackensack University Medical Center, Hackensack, NJ, USA, Hackensack, NJ, USA; ^3^Seattle Children’s Research Institute, University of Washington, Seattle, WA, USA; ^4^University of Calgary, Calgary, AB, Canada; ^5^Childhood Arthritis and Rheumatology Research Alliance, Inc./Duke Clinical Research Institute, Durham, NC, USA; ^6^Boston Children’s Hospital, Boston, MA, USA

**Background**

The overall aims are to: (1) determine the predominant circulating cellular phenotype, cytokine profile and autoantibody profile in pediatric systemic sclerosis (SSc) and localized scleroderma (LS) patients and (2) to test the ability of these immunophenotypes to predict disease manifestations, response to therapy, and define biological subsets of LS and SSc patients. Childhood Arthritis and Rheumatology Research Alliance (CARRA) centers interested in scleroderma will collect standardized outcome measures and biological specimens longitudinally. Preliminary immunophenotyping from the lead center is described and may serve as a platform for expansion of analyses of the larger sample set.

**Methods**

Eighteen CARRA centers were identified that could adequately enroll subjects and dedicate time and effort to this project. Disease-specific pediatric LS and SSc case report forms (CRFs) were designed and implemented into a REDCap database. Institutional review board (IRB) approval was obtained. Preliminary immunophenotyping was performed on the lead center’s pediatric LS and SSc cohort, investigating circulating cellular and cytokine/chemokine phenotypes of interest, to address the proposed aims and establish a statistical methodology template for the inclusion of data from multiple centers.

Plasma longitudinally obtained from 87 pediatric patients (n=70 LS, n=8 SSc, n=9 healthy) were examined via 60 analyte Luminex panel to compare active and inactive disease states. PBMC flow cytometry was performed (n =35 LS, n=8 SSc, n=12 healthy) with T-cell and monocyte markers. The modified Localized Scleroderma Severity Index (mLoSSI) anchored correlative cytokine/chemokine and cellular phenotype results for LS subjects, followed by multiple regression analyses of the significantly correlated cytokines using R language to improve prediction models of disease.

**Results**

Preliminary data from the lead center demonstrates moderate to strong correlations between interferon-gamma (IFN-γ) inducible cyto/chemokines and T_H_1-like associated cellular subtypes (T_H_1, Tc1) with the mLoSSI (*Table 1*). Of these, CXCL9 and IL-10 were statistically significant predictors in multiple linear regression analyses (*p* = 0.001 and 0.002, respectively), with an adjusted R-squared of 0.71; meaning the change of CXCL9 and IL-10 are related to the change in mLoSSI, signifying change in disease activity status.

**Conclusion**

The infrastructure of a multi-center translational pediatric scleroderma study established through CARRA is feasible. Preliminary immunophenotyping demonstrates an IFN-γ signature (CXCL9, CXCL10/ IP-10, IFN-γ) during active LS disease along with disease activity regulation via IL-10 production and M2 phenotype. Further analyses are underway using a best fit modeling approach to find the strongest core set of biomarkers/immunophenotype that highly predicts the mLoSSI in patients with LS, signifying degree of disease activity. Additional longitudinal samples across all 18 pediatric scleroderma CARRA sites will be collected and analyzed using this approach to determine core biomarkers for LS and SSc disease activity.

**Table 1:** **(abstract O1).** Correlation of the mLoSSI to the peripheral blood cyto/chemokine and cellular subtypes

Cyto/chemokine	Spearman’s correlation	p-value	Cell subtype	Spearman’s correlation	p-value
IP-10 (CXCL10)	0.57	0.0112	Monocyte CD40+CD163+	0.89	0.0029
IL-10	0.46	0.0005	T_H_1 (TCRαβ+CD4+IFNγ+)	0.87	0.0103
MIG (CXCL9)	0.42	0.0018	T_C_1 (TCRαβ+CD8+IFNγ+)	0.75	0.0544
GM-CSF	0.38	0.0049			
IFN-γ	0.33	0.0161			
MIP-3β	0.30	0.0255			
TNF-α	0.30	0.0266			

**Acknowledgements**

This study received funding from the Scleroderma Foundation through the SF Collaborative Research (SCORE) award, Childhood Arthritis and Rheumatology Research Alliance (CARRA) small grant award, and support from the Nancy Taylor Foundation for Chronic Diseases Inc.

## O2 Increased Exposure to Adverse Childhood Experiences is Associated with Arthritis in a National Sample of US Youth

### Tamar Rubinstein^1^, Danielle R. Bullock^2^, Kaveh Ardalan^3^, Wenzhu Mowrey^1^, Nicole Brown^1^, Ruth E. Stein^1^

#### ^1^Albert Einstein College of Medicine/Children’s Hospital at Montefiore, Bronx, NY, USA; ^2^University of Minnesota/Masonic Children’s Hospital, Minneapolis, MN, USA; ^3^Northwestern University Feinberg School of Medicine/Ann & Robert H. Lurie Children’s Hospital of Chicago, Chicago, IL, USA

##### **Correspondence:** Tamar Rubinstein

**Background**

Adverse Childhood Experiences (ACEs) such as witnessing violence in the home or neighborhood, parental incarceration, and racial or ethnic discrimination are associated with increased risk of chronic disease and poorer health in children and adults. Emerging data suggest an association between exposure to ACEs and autoimmune diseases in adults, but the relationship between ACEs and childhood-onset rheumatologic diseases has not been examined. Our objective was to investigate the relationship between ACEs and arthritis, the most common manifestation of childhood-onset rheumatologic disease.

**Methods**

We used data from the 2016 National Survey of Children’s Health (NSCH) to describe the distribution of ACEs among children with current arthritis compared to 1) children with other chronic acquired physical conditions (CAPC)* and 2) all other children. The NSCH is a survey of sampled households with children <18 years conducted by the National Center for Health Statistics at the Centers for Disease Control. Children were determined to have current conditions, including arthritis, if their guardian indicated yes to both “has a doctor or other health care provider ever told you that this child has…” and “does this child currently have the condition?” We performed bivariate and multivariable logistic regression to determine associations between arthritis and cumulative ACE scores, measured as a categorical variable (0 ACEs, 1 ACE, 2-3 ACEs, >=4 ACEs). Logistic regression was also utilized to determine the relationship between cumulative ACE exposure and comorbid depression/anxiety.

**Results**

Among 50,212 children included in the survey, 16,892 had CAPC and 138 had current arthritis. More children with arthritis had any ACE exposure compared to both children with other CAPC (63.4% vs 46.9%, p<0.001) and all other children (40.1%, p<0.001). The prevalence of all individual ACE categories except racial/ethnic discrimination was significantly higher among children with arthritis compared to other CAPC and all other children. A graded relationship was observed between ACE scores and arthritis in logistic regression models for 1) children with CAPC and 2) all children. An overall significant association between ACE score and arthritis persisted after adjusting for confounders in both models (Table 1). No significant interaction was found between ACE and minority status or between ACE and poverty. Among youth with current arthritis, a graded relationship was observed between ACE scores and odds of comorbid depression/anxiety (Table 2).

**Conclusions**

A markedly high prevalence of ACEs is reported among youth with arthritis from a large national survey. Higher ACE scores were associated with increased odds of arthritis. Future investigations should examine how adversity may play a role in arthritis development, disease severity, and physical and mental health outcomes. Protective psychosocial factors, such as resilience, may mitigate the impact of ACEs and should be studied further to inform development of effective interventions.

*Allergies, asthma, diabetes, and epilepsy.

**Ethics Approval**

Approval for exemption was obtained from the Einstein-Montefiore Institutional Review Board.

## ᅟ

**Table 1:** **(abstract O2).** Odds Ratios (OR) for Arthritis by Adverse Childhood Experience (ACE) Exposure

Number of ACEs	Arthritis among children with CAPC*			Arthritis among all children**		
	OR	Confidence Interval	*p* value	OR	Confidence Interval	*p* value
0 ACEs	1	---	---	1	---	---
1	1.17	0.66 - 2.08	0.59	1.45	0.82 - 2.57	0.20
2-3	2.38	1.55 - 3.67	<0.001	3.29	2.13 - 5.05	<0.001
>=4	3.39	2.02 - 5.68	<0.001	5.20	3.11 - 8.70	<0.001
Adjusted models^						
	OR^	Confidence Interval	p value	OR^	Confidence Interval	p value
0 ACEs	1	----	----	1	---	---
1	1.01	0.57 - 1.79	0.98	1.09	0.61 - 1.94	0.77
2-3	1.98	1.27 - 3.09	0.002	2.33	1.50 - 3.63	<0.001
>=4	2.54	1.48 - 4.36	0.001	3.30	1.93 - 5.65	<0.001

* CAPC = Chronic acquired physical conditions: allergies, asthma, arthritis, diabetes, and epilepsy.

** Children with a history of arthritis, but no current arthritis, were censored from this analysis.

^ Adjusted for age, sex, minority race/ethnicity, and poverty status.

**Table 2:** **(abstract O2).** Increased Odds Ratio (OR) of Comorbid Depression/Anxiety by Level of ACE Exposure in Youth with Arthritis

	Youth with current arthritis (N = 123)		
Cumulative ACE exposure	OR	Confidence Interval	p value
1 ACE vs none	4.48	1.22 – 16.47	0.024
2-3 ACEs vs none	5.30	1.87 – 15.05	0.002
≥4 ACEs vs none	12.47	3.70 – 42.00	<0.001

## O3 Working Towards Comparative Effectiveness Studies for Moderate to Severe Juvenile Localized Scleroderma: Results of a 1-Year Pilot Study of Three Methotrexate-Based Consensus Treatment Plans

### Suzanne Li^1^, Kathryn Torok^2^, Sandy Hong^3^, Polly Ferguson^3^, C. Egla Rabinovich^4^, Mara Becker^5^, Maria Ibarra^5^, Fatma Dedeoglu^6^, Robert Fuhlbrigge^7^, Katie Stewart^8^, Elena Pope^9^, Ronald Laxer^9^, Tom Mason^10^, Gloria Higgins^11^, Marilynn Punaro^8^, Laura Schanberg^4^, Tracy Andrews^12^, for the CARRA Registry

#### ^1^Hackensack University Medical Center, Hackensack, NJ, USA; ^2^University of Pittsburgh, Pittsburgh, PA, USA; ^3^University of Iowa, Iowa City, IA, USA; ^4^Duke University, Durham, NC, USA; ^5^Children's Mercy Hospital, Kansas City, MO, USA; ^6^Boston Children's Hospital, Boston, MA, USA; ^7^Children's Hospital of Colorado, Denver, CO, USA; ^8^Texas Children's Hospital, Dallas, TX, USA; ^9^Hospital for Sick Kids, Toronto, CA; ^10^ Mayo Clinic, Rochester, MN, USA; ^11^Nationwide Children's Hospital, Columbus, OH, USA; ^12^Hackensack University Medical Center, Hackensack, NJ, USA

##### **Correspondence:** Suzanne Li

**Background**

Juvenile localized scleroderma (jLS) is an inflammatory and fibrosing disease associated with major morbidity including hemiatrophy and arthopathy. Treatment is focused on controlling inflammation to limit damage. Optimal treatment is not known and regimens differ greatly. To work towards comparative effectiveness studies, standardized treatment regimens (consensus treatment plans, CTPs) were developed by an LS Childhood Arthritis Rheumatology Research Alliance (CARRA) group. We report on our pilot study of these CTPs.

**Methods**

We conducted a prospective observational study at 10 CARRA sites. Eligible subjects had a diagnosis of jLS, fulfilled activity criteria, and were willing to be treated with one of three CTPs: methotrexate (mtx) alone (A), mtx + intravenous corticosteroids [CS] (B), or mtx + oral CS (C). Treating physicians chose the specific CTP for their patients. Subjects were ineligible if they had received CS in preceding 2 weeks, or mtx or mycophenolate mofetil in preceding 3 months. Assessments included clinical scoring of lesion features; physician global assessments (PGA), physician assessment of activity status compared to baseline (MD Activity); and patient and parent health related quality of life (HRQOL) measures. Data was captured in the CARRA Legacy Registry. Analyses included Chi-Square or Fisher’s exact tests for categorical data, Wilcoxon Rank Sum Tests or Sign Rank Tests, and Spearman’s correlation.

**Results**

We met our target enrollment (50), with subjects enrolled into all CTPs. Subjects were typical for jLS, with most new to systemic treatment (Table 1).

**Table 1:** **(abstract O3).** Subject characteristics

	All, # (%) 50 subjects	CTP A, # (%) 15 subjects	CTP B, # (%) 24 subjects	CTP C, # (%) 11 subjects	P value
Female	35 (70.0)	11 (73.3)	16 (66.7)	8 (72.7)	NS
Caucasian	46 (92.0)	13 (86.7)	23 (95.8)	10 (90.9)	NS
Age of onset Median (IQR)	9.62 (6.3, 11.6)	9.30 (6.9, 11.9)	9.27 (4.8, 10.5)	10.3 (5.7, 13.4)	NS
Age at study entry Median (IQR)	13 (9.8, 14.5)	13.2 (9.8, 14.1)	11.9 (6.8, 15.2)	13.3 (11.2, 14.3)	NS
Subtype:					0.011
Circumscribed superficial	4 (8.0)	0	1 (4.2)	3 (27.3)	
Circumscribed deep	3 ( 6.0)	0	0	3 (27.3)	
Linear head	12 (24.0)	3 (20.0)	6 (25.0)	3 (27.3)	
Linear trunk/limb	18 (36.0)	8 (53.3)	9 (37.5)	1 (9.1)	
Generalized	5 (10.0)	2 (13.3)	3 (12.5)	0	
Pansclerotic	1 (2.0)	0	1 (4.2)	0	
Mixed	6 (12.0)	2 (13.3)	4 (16.7)	0	
Anatomic Location					
Head	20 (40.0)	4 (26.7)	11 (45.8)	5 (45.5)	NS
Trunk	23 (46.0)	7 (46.7)	11 (45.8)	5 (45.5)	NS
Limb	29 (58.0)	11 (73.3)	15 (62.5)	3 (27.3)	0.052
Prior Systemic Treatment	9 (18.0)	2 (13.3)	7 (29.2)	0	0.097
ANA positivity	23 (54.8)	5 (41.7)	15 (79.0)	3 (27.3)	0.013
Extracutaneous morbidity	37 (74.0)	11 (73.3)	18 (75.0)	8 (72.7)	NS
Deviation from CTP	24 (48.0)	8 (53.3)	11 (45.8)	5 (45.5)	NS
MD Activity status at 12 months vs baseline					NS
Major improvement	22 (50.0)	4 (33.3)	13 (56.5)	5 (55.5)	
Moderate improvement	15 (34.1)	6 (50.0)	7 (30.4)	2 (22.2)	
Mild improvement	6 (13.6)	2 (16.7)	2 (8.7)	2 (22.2)	
Mild worsening	1 (2.3)	0	1 (4.4)	0	
Inadequate response to CTP	13 (26.0)	4 (33.3)	8 (33.3)	1 (9.1)	NS
Any Adverse Event	33 (66.0)	7 (46.7)	17 (70.8)	9 (81.8)	NS
AE grade 1	26 (52.0)	6 (40.0)	11 (45.8)	9 (81.8)	NS
AE grade 2	22 (44.0)	4 (26.7)	14 (58.3)	4 (36.4)	NS
AE Grade 3	1 (2.3)	0	1 (4.4)	0	NS

At the 12-month visit, most subjects were rated as having a major or moderate improvement in activity status compared to baseline with no differences across groups (Table 1). Adverse events were common (Table 1), and typical for mtx and CS. There was one grade 3, hospitalization for viral gastroenteritis and dehydration. Almost half the subjects had a major deviation from initial CTP, with most common reasons medication intolerance resulting in dose adjustment or medication change (30%), inadequate response (26%), and non-compliance (18%). The PGA-Activity and Skin Activity scores changed significantly between the baseline and last visit, while PGA-Damage, Skin Damage scores, and HRQOL did not (Table 2).

**Table 2:** **(abstract O3).** Comparison of Medians Between Baseline and Last Visit

	All Subjects		
Covariate	Baseline: median (IQR)	Last Visit: median (IQR)	P-Value
PGA-Activity	4 (3,6)	0.5 (0,2)	<0.001
PGA-Damage	4 (3,6)	3 (2,5)	0.089
Overall HRQOL	2 (1,2)	2 (1,2)	0.311
CHAQ	0 (0,0.3)	0.1 (0,0.5)	<0.001
Pain Score	0 (0,2)	1 (0,4)	<0.001
Skin Activity score: consists of these variables:	6 (4,11)	1 (0,2)	<0.001
New lesion	1 (1,2)	0 (0,0)	<0.001
Erythema	2 (1,4)	0 (0,0)	<0.001
Violaceous color	0 (0,1)	0 (0,0)	0.001
Waxy white or Yellow	1 (0,2)	0 (0,1)	<0.001
Tactile Warmth	0 (0,1)	0 (0,0)	<0.001
Skin thickening of edge	1 (0,2)	0 (0,0)	<0.001
Skin Damage score: consists of these variables:	12 (4,19)	10 (5,16)	0.071
Skin thickening of center	2 (0,3)	1 (0,2)	<0.001
Dermal atrophy	3 (1,5)	3 (2,5)	0.690
Subcutaneous atrophy	2 (1,5)	2 (1,5)	0.474
Hyperpigmentation	2 (0,5)	2 (1,4)	0.493
Hypopigmentation	1 (0,2)	1 (0,1)	0.147

Significant correlations were found between PGA-Activity and MD Activity (r = -0.543), and between PGA-Activity and activity variables, with the highest correlation with erythema and white waxy (r=0.627, 0.589, respectively). PGA-Damage was associated with subcutaneous and dermal atrophy, and hyperpigmentation (r=0.693, 0.616, 0.590, respectively).

**Conclusions**

We demonstrate the feasibility of comparative effectiveness studies in jLS. Most subjects were rated as having major or moderate improvement irrespective of CTP. About a quarter of the subjects were considered to have an inadequate response. Larger studies are needed to assess relative treatment efficacy and factors associated with inadequate response.

**Acknowledgements**

The authors gratefully acknowledge funding for this study from an Arthritis Foundation Innovative Research Grant and CARRA, and funding for the CARRA Legacy Registry from NIAMS, Friends of CARRA, and Arthritis Foundation.

## O4 Biologic switching among JIA patients: a cohort study in the Childhood Arthritis and Rheumatology Research Alliance Registry

### Melissa L. Mannion^1^, Fenglong Xie^1^, Daniel B. Horton^2^, Sarah Ringold^3^, Colleen K. Correll^4^, Anne Dennos^5^, Timothy Beukelman^1^, for the CARRA Registry Investigators

#### ^1^University of Alabama at Birmingham, Birmingham, AL, USA; ^2^Rutgers University, New Brunswick, NJ, USA; ^3^Seattle Children’s Hospital, Seattle, WA, USA; ^4^University of Minnesota, Minneapolis, MN, USA; ^5^Duke Clinical Research Institute, Durham, NC, USA

##### **Correspondence:** Melissa L. Mannion

**Background**

Biologic medications have allowed a significant proportion of JIA patients to achieve inactive disease. However, some patients will have ongoing moderate to high disease activity or will not tolerate the medication. Current treatment recommendations suggest changing biologic medications when inactive or low disease activity is not attained, but the switching patterns and reasons for switching in clinical practice in North America are not currently known.

**Methods**

We used the Childhood Arthritis & Rheumatology Research Alliance (CARRA) Registry of clinical data from >55 pediatric rheumatology clinics in the United States and Canada. Individuals with JIA were included if they newly started a biologic medication after January 1, 2008 and had a minimum of 12 months of observable time following medication start. They were censored at the most recent visit. Individuals with systemic JIA were excluded. Subjects were labelled switchers if they had subsequent use of any other biologic medication with <6 months between discontinuation of the 1^st^ and start of the 2^nd^ biologic and were labelled delayed switchers if the time between biologics was >6 months. Subjects were labelled non-switchers if they had no subsequent biologic medication use in the data. We compared characteristics of switchers, delayed switchers, and non-switchers using descriptive statistics, chi-square for categorical variables, and Wilcoxon rank sum testing for continuous variables.

**Results**

There were 708 children with JIA who newly started a biologic medication in the medication log, of whom 146 (21%) were switchers and 45 (6%) were delayed switchers (Table 1).

The median time before switching was 434 days (interquartile range (IQR) 194 - 924) and the median time between the first and second biologic medications was 14 days (IQR 0 – 159). The majority of patients had started on etanercept (476, 67%). There was no difference in the presence of uveitis or inflammatory bowel disease between those who did and did not switch. Among the switchers, the most common reason for switch was inefficacy (77, 52%), followed by disease flare (19, 13%), and intolerance of medication or delivery method (16, 11%).

**Conclusion**

In a multicenter cohort of children with JIA who started a biologic, 20% of children switched biologic medication after a median of 14 months. Etanercept was the most common first biologic medication. Additional studies are needed to evaluate the clinical predictors of switching, the outcomes following biologic switching, to identify the optimal timing of switching and the preferred second-line agent.

**Table 1:** **(abstract O4).** Characteristics of biologic initiators in the CARRA Registry

	All biologic initiators N= 708	Non switchers n=517	Switchers n=146	Delayed switchers n=45	Comparison of non-switchers vs switchers P value
Age (years) median, range, IQR	10 (IQR 6, 13)	10 (6, 13)	10.5 (8, 14)	8 (4, 11)	ns
Sex (F) n, %	547 (77%)	388 (75%)	119 (82%)	40 (89%)	<0.05
Race: n, %					
White	582 (82%)	427 (83%)	117 (80%)	38 (84%)	ns
Black	27 (4%)	19 (4%)	5 (3%)	3 (7%)	ns
Asian	29 (4%)	17 (3%)	9 (6%)	3 (7%)	ns
Hispanic, Latino	68 (10%)	55 (10%)	11 (8%)	2 (4%)	ns
Other	13 (2%)	7 (1%)	5 (3%)	1 (2%)	ns
JIA subtype: n, %					ns
RF+ poly	90 (13%)	69 (13%)	16 (11%)	5 (11%)	
RF- poly	376 (53%)	278 (54%)	71 (49%)	27 (60%)	
Persistent Oligo	66 (10%)	51 (10%)	11 (8%)	4 (9%)	
Extended oligo	44 (6%)	32 (6%)	10 (7%)	2 (4%)	
ERA	72 (10%)	49 (10%)	21 (14%)	2 (4%)	
psoriatic	44 (6%)	27 (5%)	14 (10%)	3 (7%)	
undifferentiated	15 (2%)	10 (2%)	3 (2%)	2 (4%)	
Uveitis n, %	48 (7%)	35 (7%)	9 (6%)	4 (9%)	ns
IBD n, %	18 (3%)	14 (3%)	3 (2%)	1 (2%)	ns
Time from diagnosis to start biologic (days) median, range, IQR	217 (66-1126)	218 (70, 1113)	209 (54, 1211)	217 (75, 927)	ns
Time from disease onset to diagnosis (days, median, IQR)	119 (48-298)	119 (47, 305)	118 (44, 278)	135 (52, 232)	ns
MTX use (all) n, %	554 (78%)	394 (76%)	119 (82%)	41 (91%)	<0.05
PO MTX n, %	249 (35%)	186 (36%)	45 (31%)	18 (40%)	ns
SQ MTX n, %	389 (55%)	272 (53%)	87 (60%)	30 (67%)	ns
sulfasalazine use n, %	36 (5%)	25 (5%)	8 (5%)	3 (7%)	ns
Leflunomide use n, %	16 (2%)	12 (2%)	3 (2%)	1 (2%)	ns
Calendar year of biologic start: median, IQR	2014 (2012-2015)	2014 (2013, 2015)	2014 (2012, 2015)	2012 (2010, 2014)	<0.05
Medication starting: n, %					ns
TNF (all)					
etanercept	476 (67%)	336 (65%)	103 (71%)	37(82%)	
adalimumab	152 (21%)	118 (23%)	29 (20%)	5 (11%)	
infliximab	36 (5%)	30 (6%)	3 (2%)	3 (7%)	
golimumab	3 (0.4%)	2 (0.4%)	1 (0.7%)	0	
certolizumab	3 (0.4%)	3 (0.6%)	0	0	
Non TNF (all)					
tocilizumab	14 (2%)	12 (2%)	2 (1%)	0	
abatacept	17 (2%)	11 (2%)	6 (4%)	0	
rituximab	3 (0.4%)	3 (0.6%)	0	0	
ustekinumab	0	0	0	0	
anakinra	3 (0.4%)	1 (0.2%)	2 (1%)	0	
canakinumab	1 (0.1%)	1 (0.2%)	0	0	

IQR, interquartile range; JIA, juvenile idiopathic arthritis; RF, rheumatoid factor; ERA, enthesitis related arthritis; IBD, inflammatory bowel disease; MTX, methotrexate; PO, oral; SQ, subcutaneous; TNF, tumor necrosis factor inhibitor.

**Ethics Approval**

The study was approved by University of Alabama's Institutional Review Board, protocol number X170112004.

## P1 Feasibility of Conducting Epigenetic Analysis in Pediatric Lupus B Cells

### Joyce S. Hui-Yuen^1,2^, James N. Jarvis^3^

#### ^1^Division of Pediatric Rheumatology, Cohen Children’s Medical Center, Lake Success NY; ^2^Department of Pediatrics, Hofstra-Northwell Health School of Medicine, Hempstead NY; ^3^Genetics, Genomics, and Bioinformatics Program, University at Buffalo, Buffalo NY

**Background**

Bennett et al identified the presence of interferon gene expression signatures in peripheral blood of children with systemic lupus erythematosus (pSLE). Here, we aim to identify how B cells contribute to those signatures. We hypothesize that disordered transcription in pSLE will be prominent in B cells and attributed to disease-specific epigenetic alterations. Thus we will not only identify important disease mechanisms in SLE, we will shed light on the genetics of SLE. Our previously published work demonstrates that most genetic risk for SLE is located within non-coding regions of the genome, where epigenetic modifications of DNA and histone proteins regulate and coordinate transcription.

**Methods**

Our specific aim is to assess regions of open chromatin in untreated pSLE and compare findings with healthy children. We propose to use assays of transposase-accessible chromatin with sequencing (ATACseq) to broadly survey open regions of chromatin and clarify the functional epigenome. In this pilot study, we propose to determine feasibility of performing this assay and developing methods for data analysis using 5 pediatric lupus patients and 5 healthy children.

**Anticipated Results and Conclusion**

Our long-term goal is to gain a mechanistic understanding of the aberrant transcriptional signatures in untreated SLE. The data generated by this pilot study will provide the basis for a rigorous power analysis, and firmly establish the working relationship between the Buffalo and Cohen Children’s Medical Center groups, both of which will be essential for a competitive application to NIH. Our preliminary findings from this pilot study will also allow us to begin the exciting process of linking the genetics and epigenetics of pSLE to the well-established transcriptional aberration.

**Ethics Approval**

The study was approved by the Northwell Health Institutional Review Board, approval number HS 07-021.

**Funding**

This study is supported by a CARRA-AF Small Grant for 2018.

## P2 Kappa-Deleting Recombination Excision Circles (KREC) in B Cells: an Aid in Predicting Juvenile Dermatomyositis Response to Rituximab

### Amer Khojah^1,2^, Victoria Hans^3^, Michael Miller^1,4^, Marisa Klein-Gitelman^1,4^, Megan Curran^1,4^, Ramsay Fuleihan^2,4^, Gabrielle Morgan^3^, Chiang-Ching-Huang^5^, Lauren M. Pachman^1,3,4^

#### ^1^Division of Rheumatology, Ann & Robert H. Lurie Children's Hospital of Chicago, Chicago, IL, USA; ^2^Division of Allergy & Immunology, Ann & Robert H. Lurie Children's Hospital of Chicago, Chicago, IL, USA; ^3^Cure JM Center of Excellence, Stanley Manne Research Center, Chicago, IL, USA; ^4^Northwestern University Feinberg School of Medicine, Chicago, IL, USA; ^5^Joseph J. Zilber School of Public Health, University of Wisconsin, Milwaukee, WI, USA

##### **Correspondence:** Amer Khojah

**Background**

Rituximab is used for the treatment of juvenile dermatomyositis (JDM) with variable success. Some of this variability is presumed to be related to the effectiveness of tissue B cells depletion. Forming new B cells requires B cell receptor recombination, which leads to the formation of KREC, a circular DNA that does not replicate as B cells divide, so the KREC remains only in one cell and not the progeny. The recombination site on chromosomal DNA after the excision of KREC is known as the joining code (JC), found in all B cell progeny. The ratio of JC to KREC determined by qRT-PCR estimates the number of B cell divisions that have occurred in the patient’s B cell population. We hypothesized that rituximab treated JDM who have complete or near complete B cell depletion will have a low JC to KRECs ratio which is associated with a more effective response to rituximab.

**Methods**

This is an IRB approved, retrospective study conducted at The Cure JM Center of Excellence, Lurie Children’s Hospital. We included all 10 JDM patients (mean age of 12.5 years, 6 females, 7 Caucasians, 7 subjects with P155/140+ autoantibody) who had received rituximab therapy and had serial PBMC stored (-80^o^C) in our repository. DNA was isolated stored PBMCs; qRT-PCR measured JC and KREC. We defined oligoclonal B cell expansion as JC:KRECs ratio of 8 or more before rituximab therapy. A JC:KRECs ratio of 2 or less on the first detectable sample post-rituximab was considered as an evidence of good B cell depletion. We defined a good response to therapy as improvement of the Disease Activity Score (DAS) by at least 2 points in two consecutive visits. This project was funded by Children’s Arthritis and Rheumatology Research Alliance (CARRA).

**Results**

JC and KREC were detectable in all samples that had B cell count of 10cell/mm^3^ or more. 6 out 10 JDM patients had evidence of oligoclonal B cell expansion. Of the 6 subjects with oligoclonal B cell expansion, three had good B cell depletion (JC:KREC ratio of 2 or less) and responded well to rituximab therapy (Fig1). On the other hand, 2 out 3 subjects with poor B cells depletion had a lack of response to rituximab (Fig2). The majority of the subjects (3 out of 4) without oligoclonal B cell expansion had a poor response to rituximab therapy (Fig 3).

**Conclusions**

In this study, we showed that qRT- PCR of JC and KREC can replace B cell count by flow cytometry to monitor B cell reconstitution. Furthermore, this method can differentiate between the B cells that come from the bone marrow vs peripheral B cell expansion. Subjects with oligoclonal B cell expansion have a more favorable response to rituximab. If this finding is confirmed in a larger patient cohort, it may serve as a criterion for continuing rituximab therapy, thus improving the patient’s outcome.

**Fig. 1:**
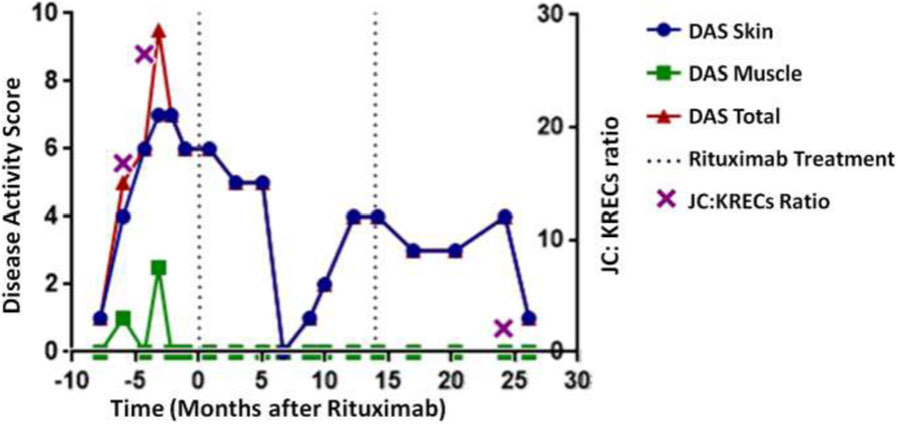
**(abstract P2).** JDM subject responded well to rituximab therapy who had evidence of oligoclonal B cell expansion and good B cell depletion based on the JC: KRECs ratio

**Fig. 2:**
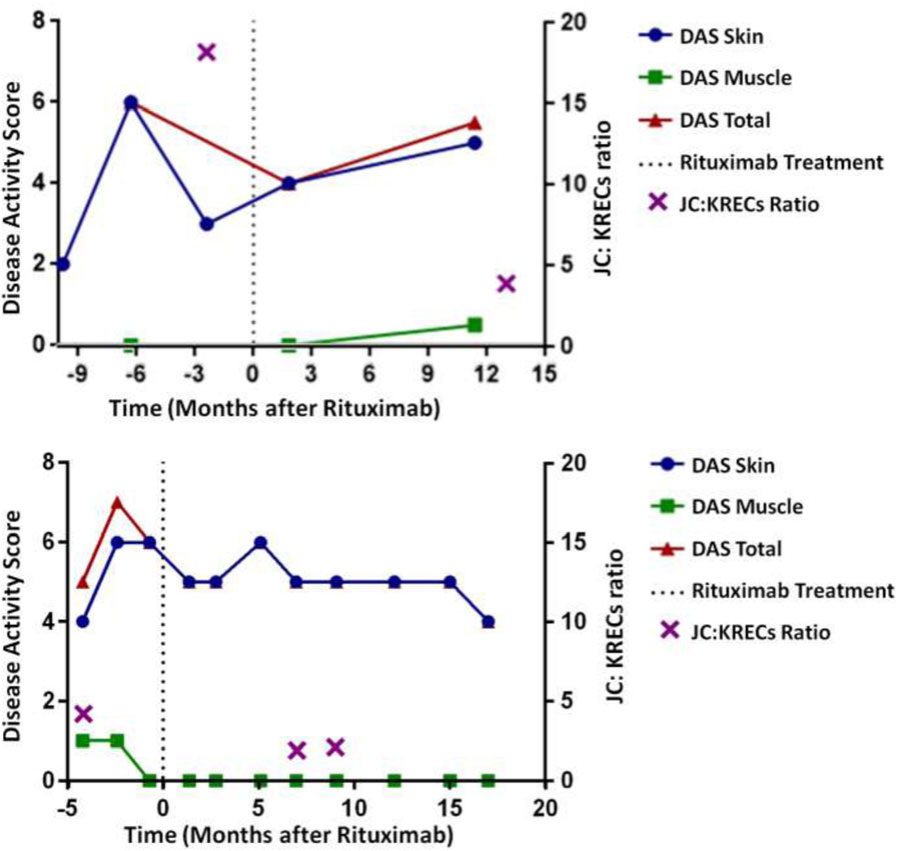
**(abstract P2).** JDM subject who had poor B cell depletion and lack of response to rituximab therapy

## P3 Nasopharyngeal Pneumococcus Colonization in patients with Childhood Onset Systemic Lupus Erythematosus (cSLE)

### Fatima Barbar-Smiley^1,2^, Stacy Ardoin^1,2^, Vidya Sivaraman^1,2^, Cagri Yildirim Toruner^1,2^,Chack Yung Yu^1,3^, Octavio Ramilo^1,3^

#### ^1^Nationwide Children's Hospital, Columbus, OH, USA; ^2^The Ohio State University College of Medicine, Columbus, OH, USA; ^3^The Research Institute at Nationwide Children's Hospital, Columbus, OH, USA

##### **Correspondence:** Fatima Barbar-Smiley

**Background**

Pneumococcal infections are significant causes of morbidity and mortality in both the general and immunocompromised populations. *Streptococcus pneumoniae* may lead to severe infections. Lupus patients are five times more likely to have the invasive pneumococcal disease than healthy controls. The CDC recommends administering pneumococcal vaccination for children with lupus who are immunosuppressed. Studies have shown an association between nasopharyngeal (NP) pneumococcal colonization and increased risk of infection with this pathogenic bacterium. Vaccine effect on pneumococcal NP colonization (VE-col) is a proper surrogate marker for vaccine effect on invasive pneumococcal disease. To date, there are no data on the rate nasopharyngeal pneumococcal colonization in children diagnosed with cSLE and how it correlates with the incidence of invasive disease. In this study, we assess rate and density of pneumococcal nasopharyngeal colonization following receipt of recommended pneumococcal vaccination in cSLE patients compared with age-matched healthy controls.

**Methods**

We will perform a prospective case-control study involving cSLE versus healthy controls at a single center, large free-standing children’s hospital. We will collect one NP swab and analyze for quantitative pneumococcal PCR. Positive samples will be saved to study specific serotypes in the future. We will collect the following information from the medical records: demographics, medication list, immunization history, SLE disease activity index, laboratory results. Rates of positive NP swabs will be compared between patients with cSLE versus healthy controls using chi-square or Fisher’s exact tests. We will examine the density of colonization by two-sample t-tests or Wilcoxon rank sum and chi-square tests for trend. We will compare clinical and patient characteristics by cSLE status and by NP positivity using chi-square or Fishers’ exact tests for continuous variables and two-sample t-tests or Wilcoxon rank sum tests for continuous variables. A multivariable logistic regression model will be assessed to determine whether cSLE status is associated with higher likelihood of positive NP swabs while accounting for possible confounders.

**Results/Conclusion**

We anticipate that patients with cSLE will have higher rate and density of positive pneumococcal NP swabs following receipt of the recommended pneumococcal vaccinations as compared with healthy controls. Results will help us better understand indirect markers for immune response to pneumococcal vaccination and to explore factors that may impact the response to pneumococcal vaccine in childhood lupus. Following this study, we plan to carry in-depth mechanistic studies for serum vaccine response and genetics that will help with providing precision medicine approach to vaccinations in cSLE and other autoimmune diseases.

**Ethics Approval**

Study was approved by the local Institutional Review Board (IRB) IRB17-00018.

**Funding**

This research study is supported by Childhood Arthritis and Rheumatology research Alliance- Arthritis Foundation (CARRA-AF) small grant award.

## P4 Stopping medicines for inactive juvenile idiopathic arthritis: what do patients and families consider?

### Daniel B. Horton^1,2^, Jomaira Salas^3^, Aleksandra Wec^4^, Tim Beukelman^5^, Alexis D. Boneparth^6^, Jaime Guzman^7^, Ky Haverkamp^8^, Melanie Kohlheim^9^, Melissa L. Mannion^5^, Lakshmi N. Moorthy^1^, Elizabeth Stringer^10^, Lori Tucker^7^, Sarah Ringold^11^, Marsha Rosenthal^2^

#### ^1^Rutgers Robert Wood Johnson Medical School, New Brunswick, NJ, USA; ^2^Rutgers Institute for Health, Health Care Policy and Aging Research, New Brunswick, NJ, USA; ^3^Department of Sociology, Rutgers, the State University, New Brunswick, NJ, USA; ^4^Mathematica Policy Research, Princeton, NJ, USA; ^5^University of Alabama at Birmingham, Birmingham, AL, USA; ^6^Columbia University Medical Center, New York, NY, USA; ^7^British Columbia Children’s Hospital, University of British Columbia, Vancouver, BC, Canada; ^8^University of Washington School of Medicine, Seattle, WA, USA; ^9^Pediatric Rheumatology Care and Outcomes Improvement Network, Cincinnati, OH, USA; ^10^IWK Health Centre, Dalhousie University, Halifax, NS, Canada; ^11^Seattle Children's Hospital, Seattle, WA, USA

**Background**

Prior research has examined factors important to clinicians in deciding whether to withdraw therapy for inactive JIA, but few studies have investigated what is important to patients with JIA and their families. Recently, we interviewed patients and their caregivers about stopping JIA medications. One of the major themes to emerge was the perceived trade-off between risks and fears of medicines and risks and fears of the disease itself. This trade-off was influenced strongly by negative prior experiences and complications of JIA or its treatment as well as perceived threats of long-term damage. We administered an anonymous online survey to learn how widespread these beliefs and actions are.

**Methods**

We recruited participants via social media, email, and paper advertisements in pediatric rheumatology clinics in the US and Canada, in collaboration with PARTNERS (Patient, Advocates, and Rheumatology Teams NEtwork for Research and Service). The online survey was administered via REDCap hosted at Rutgers University and was available in English and Spanish. The format and questions of the survey were based on the findings from semi-structured interviews exploring decision-making around withdrawing JIA treatment. Questions were refined with additional input from patients and parents who pilot tested the survey. Participant enrollment will conclude in February 2018.

**Results**

To date, 130 participants have completed the online survey, including 108 parents, 17 young adults, and 5 adolescents. Participants reported living in the USA (103, 82%), Canada (16, 12%), or other countries (8, 6%). Participants were asked to rate and rank a number of disease- and treatment-related factors that may influence decisions to stop JIA treatment, such as JIA-related damage, duration of inactive disease, concerns about flare, concerns about drug side effects or long-term effects, and cost or inconvenience of medicines. Other questions were designed to judge how reluctant or eager individuals were to stop their (or their children's) medicines, particularly in cases of disagreement with the treating clinician. Background questions asked for demographic information and their (or their children’s) history of JIA and medication use.

**Conclusion**

This online survey will provide important new information about the factors of greatest importance for patients with JIA and their families in making decisions about treatment withdrawal. It will help us better understand how people balance the trade-off between risks and fears of medicines and risks and fears of having JIA. Greater awareness of this trade-off and related influential factors may help improve shared decision-making around withdrawing treatment for inactive JIA.

**Ethics Approval**

The study was approved by the Rutgers University Institutional Review Board, protocol number Pro20160000193.

**Acknowledgements**

Funding for this work came from a CARRA-AF Small Grant.

## P5 Clinical Disease Manifestations associated with TNF inhibitor failure in Juvenile Spondyloarthritis

### Melissa Oliver^1^, Pamela F. Weiss^2^, Robert A. Colbert^3^ Hemalatha Srinivasalu^4^, for the CARRA spondyloarthropathy subcommittee

#### ^1^Indiana University, Indianapolis, IN, USA; ^2^Children’s Hospital of Philadelphia, Philadelphia, PA, USA; ^3^National Institute of Arthritis and Musculoskeletal and Skin Diseases, NIH, Bethesda, MD, USA; ^4^George Washington University School of Medicine, Washington, DC

##### **Correspondence:** Melissa Oliver

**Background**

Until recently, tumor necrosis factor inhibitors (TNFi) were the only class of biologics with demonstrable effectiveness in pediatric and adult spondyloarthritis, and thus they represent the first-line for biologic therapy in children with juvenile spondyloarthritis (JSpA). Although most patients respond well to TNFi, not all do, failing either secondary to lack of effectiveness or adverse events. Children who do not respond to initial TNFi are either switched to a different TNFi or treated with second-line biologics. We aim to quantify and describe the population of children who fail TNFi.

**Methods**

This is a multi-center retrospective cohort study of JSpA patients who have failed TNFi therapy, specifically, etanercept, adalimumab, infliximab, golimumab or certolizumab pegol. A survey will be sent to sites to collect data on JSpA patients who have failed TNFi therapy. Standardized definitions for primary and secondary treatment failure for the JSpA population based on prior literature and survey results will be outlined. Descriptive statistics will be used to summarize the survey results and clinical characteristics of JSpA patients who failed TNFi. Correlation analysis and uni- and multi-variable models will be used to assess the association of TNFi failure with clinical and laboratory attributes.

**Results**

A systematic literature review of treatment failure in the adult and pediatric inflammatory arthritis populations was performed. Based on this review, working definitions for treatment failure in JSpA population have been generated, which will be reviewed and discussed with the JSpA workgroup to develop a final standardized definition. In order to understand the various reasons for switching TNFi, an initial data generating questionnaire was developed to collect clinical and laboratory attributes of children who meet our definition of TNFi failure.

**Conclusions**

This study will educate physicians on specific disease characteristics that may be associated with TNFi failure, thereby aiding management decisions for patients with JSpA. The ability to identify patients who are more likely to fail TNFi therapy will be an important step toward tailoring patient-specific treatments. Knowledge about indicators of TNFi failure may help in targeting newer alternatives to TNFi in this particular subset of patients.

**Ethics Approval**

IRB has been accepted at Indiana University SOM.

## P6 Identifying trajectories of disease activity states in Juvenile Idiopathic Arthritis (JIA) early after treatment: shortening time to decision to change treatment

### Lily Lim^1^, Sarah Ringold^2^, Eleanor Pullenayegum^3^, Earl Silverman^4^, Fabrizio de Benedetti^5^, Laura Schanberg^6^, Yukiko Kimura^7^

#### ^1^Children’s Hospital Research Institute of Manitoba, University of Manitoba; ^2^Seattle Children’s Hospital, Seattle, WA; ^3^Child Health Evaluative Sciences, SickKids Research Institute, SickKids, Toronto, Canada; ^4^SickKids, Toronto, Canada; ^5^IRCCS Ospedale Pediatrico Bambino GesÙ; ^6^Duke Clinical Research Institute, Duke University Medical Center, Durham, NC; ^7^Hackensack University Medical Center, Hackensack, NJ

**Background**

The treatment of juvenile idiopathic arthritis (JIA) has been revolutionized by introduction of biological therapies resulting in improved disease control and decreased joint damage. However, there is heterogeneity in patients’ response times to biologics. As there are currently no data to identify patients with clinically meaningful differences in response times early, providers must wait for response and expose these children to ongoing active disease and potential joint damage. Current trial designs commonly lead in with 16 weeks of trial medication. We hypothesize that we will be able to identify individuals who will not respond, earlier than 16 weeks by studying their treatment response trajectories. This study aims to measure the probabilities of attaining clinically significant therapeutic responses as measured by the ACR Pedi response states and clinically inactive disease (CID) or juvenile arthritis disease activity state (JADAS) in trial participants before week 16. We will identify the non-responders using baseline clinical phenotype, biomarkers and demographic predictors.

**Methods**

This study uses data from prior completed trials including the Trial of Early Aggressive Therapy (TREAT), CHERISH (tocilizumab), etanercept and abatacept trials. All patients have polyarticular course JIA. The outcomes studied are the ACR Pedi 50,70,90, CID states or the JADAS low, moderate, high and inactive disease activity states. We will build two Markov multistate models (MSM) with interval censoring to determine the probabilities of attaining each state at each visit of trial assessment and transitioning between states. We will test baseline (study entry) clinical and laboratory factors for prediction of state attainment and transition. Concomitant prednisone use will be tested as a time-varying prognostic factor in the model throughout the 16 weeks.

**Results**

We have finished the negotiations of data sharing agreements (DSA) in 3 of the 4 studies. The final DSA negotiations should be concluding soon. The data transfer from one of the drug company to the analysis site is complete. The data transfer from another 2 of the trials is being arranged. Preliminary analysis will be starting in the spring of 2018.

**Conclusions**

We expect that by identifying non-responders of biological therapy earlier, the results from our study will inform future trial designs, allowing for shorter trials. This will translate into cost savings for pharmaceutical companies. The information may also be used in clinical practice to allow pediatric rheumatologists to identify non-responders early and to change their therapy accordingly, so as to avoid irreversible joint damage.

**Funding**

This study is funded via a CARRA small grant 2017.

## P7 Optimizing the electronic health record to capture Childhood Arthritis and Rheumatology Research Alliance (CARRA) Data

### Alysha J. Taxter^1^, Julisa M. Patel^1^, Ajay Dharod^2^

#### ^1^Brenner Children’s Hospital, Section of Pediatric Rheumatology, Winston-Salem, NC, USA; ^2^Wake Forest Baptist Hospital, Department of Internal Medicine, Winston-Salem, NC, USA

##### **Correspondence:** Alysha J. Taxter

**Background**

Technology has revolutionized how people gather information and communicate with medical providers. Patients desire to play an active role in their care and to participate in research. However, limited integration of technology into clinical care and research can impede their participation. Such information can easily be obtained by collecting patient-reported outcomes (PROs) on tablet devices which seamlessly integrate into the electronic health record (EHR). The goal of this project is to implement and evaluate efforts to electronically capture PROs and clinical data, and evaluate patient interest in participating in clinical research projects.

**Methods**

This is a proof of concept study in an outpatient tertiary rheumatology clinic. We delivered PRO questionnaires in English or Spanish through an electronic patient portal. The PRO questionnaire included questions about current symptoms, pain, fatigue, disease activity, function, and interest in research. We built clinic note templates in our EHR to capture pertinent CARRA registry data, including physician global disease activity assessment and active joint count. Providers can trend longitudinal data during a clinic visit using flowsheet functions which can also be shared with patients. This discrete data can also be easily abstracted using EHR reporting functions. Data capture was evaluated for validity and missingness. Clinic processes needed to accurately and timely obtain this data were evaluated with staff surveys.

**Results:**

There were 617 clinic visits from March to December 2017, with 615 (99%) PRO questionnaires completed prior to or at the clinic visit. PRO questionnaires were completed by the patient, parent, and clinic staff 39%, 48%, and 14% of the time, respectively. Of the 472 patients answering the question on interest in information on future research opportunities, 327 (70%) were interested in being contacted. We were able to utilize EHR reporting functions to abstract all patient and physician-reported variables. Providers reported this patient questionnaire and longitudinal flowsheet data saved an approximate 5 minutes per visit and allowed them to focus on pertinent positive responses. Staff surveys reported that barriers to administering questionnaires are activation of parent-proxy access and password recovery workflow.

**Conclusion:**

We successfully developed infrastructure to capture and report on both patient- and provider-entered data. Providers reported increased efficiency during visits. The majority of patients within our clinic are interested in participating in research. Clinic processes will need to be modified and staff education provided to improve access and password recovery workflow. We plan to build on this infrastructure to ultimately streamline data collection and reporting to the CARRA registry.

**Ethics Approval**

The study was approved by Wake Forest Institutional Review Board, approval number IRB00043463.

**Acknowledgements**

This work was supported by the CARRA Small Grant Application. The authors wish to acknowledge CARRA, and the ongoing Arthritis Foundation financial support of CARRA.

## P8 Utility of the pain symptom assessment questionnaire (PSAQ) to assess fibromyalgia in JIA patients

### Melissa Tesher^1^, Thomas Brent Graham^2^, Tracy V. Ting^3^, Jennifer Weiss^4^

#### ^1^University of Chicago Medical Center, Chicago, IL USA; ^2^Vanderbilt University Medical Center, Nashville TN USA; ^3^Cincinnati Children’s Hospital Medical Center, Cincinnati OH USA; ^4^Hackensack University Medical Center, Hackensack NJ USA

##### **Correspondence:** Melissa Tesher

**Background**

Pain in children with JIA has a significant impact on patient quality of life. Persistence of significant patient-reported pain despite normal objective measures of disease activity poses a quandary for the pediatric rheumatologist. The Pain Symptom Assessment Questionnaire (PSAQ) is a modified version of the 2010 ACR fibromyalgia criteria, and has demonstrated good specificity and sensitivity for diagnosis of juvenile primary fibromyalgia syndrome in adolescents. With this investigation, we aim to: 1. Evaluate the utility of the PSAQ in identifying JIA patients with Juvenile Fibromyalgia (JFM), in comparison to the Yunus and Masi criteria for JFM; and 2. Identify differences amongst JIA patients with and without JFM including potential variables such as demographic data, disease characteristics, functional disability, and physician and patient/parent global assessments.

**Methods**

Children and adolescents ages 11-17, with a known diagnosis of any subtype of JIA, are eligible for the study. We are collecting the following data from each subject:

1. Demographics: age, ethnicity, age of diagnosis/disease duration; disease subtype

2. Physician global and patient/parent global assessment

3. Active joint count, determined by physician

4. Pain VAS score (pain in the past week)

5. PSAQ (Pain Symptom Assessment Questionnaire)

6. FDI (functional disability index)

7. Tender points manual exam (performed in a subset of subjects)

**Results**

Three sites are actively enrolling subjects, with a fourth in progress (target N=160). We will use descriptive statistics to report the number of patients with JIA overall who meet criteria for JFM using the PSAQ. In addition we plan to investigate whether there is a significant relationship between: 1. JIA subtype and incidence of JFM by PSAQ; 2. Demographic and other variables (sex, active joint count, physician global assessment) and the incidence of JFM; 3. Higher score on PSAQ (more pain/symptoms) and disparity between physician and patient/parent global assessments; 4. Higher scores on the PSAQ and greater functional disability with higher pain VAS. For the subset of patients for whom the tender points exam is performed, we will assess sensitivity and specificity of the PSAQ for diagnosis of JFM, using the criteria of Yunus and Masi as the gold standard.

**Conclusions**

The study design has thus far demonstrated feasibility. We anticipate completion of data collection by summer 2018. Findings from this study may provide pediatric rheumatologists with a simple tool to evaluate JIA patients with secondary chronic widespread pain conditions whom would benefit from specific pain focused treatments.

**Ethics Approval**

This study was approved by the Institutional Review Board at the University of Chicago Medical Center, Vanderbilt University Medical Center, and Cincinnati Children’s Hospital Medical Center. IRB approval is pending at Hackensack University Medical Center.

**Funding**

This work is supported by a CARRA-AF Small Grant.

## P9 A pilot study: can musculoskeletal ultrasound (MSUS) be utilized as an objective measure of disease activity in a consensus treatment plan for patients newly diagnosed with Polyarticular Juvenile Idiopathic Arthritis (JIA)?

### Heather Benham^1^, Leandra Woolnough^1,2^, Yassine Kanaan^1^, David Wilkes^1^, Tracey Wright^1,2^ for the CARRA Investigators

#### ^1^Texas Scottish Rite Hospital for Children, Dallas, TX, USA; ^2^Department of Pediatrics, UT Southwestern Medical Center, Dallas, TX, USA

**Background**

In adult rheumatoid arthritis (RA), longitudinal studies have demonstrated correlations between clinical measures of disease activity, including the DAS-28, ESR and CRP, and objective measures of joint inflammation by musculoskeletal ultrasound (MSUS).^1,2^ Hyperemia, or increased blood flow detected by Power Doppler signal, is a key indicator of disease activity in adults with RA. The Childhood Arthritis and Rheumatology Research Alliance (CARRA) is conducting a Consensus Treatment Plan (CTP) utilizing a “treat to target” approach in newly diagnosed Polyarticular JIA patients, STOP-JIA.

The primary objective of this study is to examine the correlations between two MSUS measures of disease activity and clinical measures of disease activity including clinical active joint count, CHAQ, cJADAS-10 and ESR in patients with Polyarticular JIA followed longitudinally through treatment.

**Methods**

STOP-JIA patients were followed longitudinally with MSUS and clinical measures of disease activity including clinical active joint count, CHAQ, ESR, and cJADAS-10. MSUS assessments included scans of bilateral elbows, wrists, 2nd MCPs, knees, and ankles by grey-scale and Power Doppler. Semi-quantitative grading scales were used and pathologic lesions were measured across the greatest dimension (mm). Ultrasound Active Joint Count (USAJC) was calculated in two ways (Table 1).

**Results**

Six newly diagnosed polyarticular JIA subjects were included in this longitudinal pilot study (Table 2). Mean disease duration at baseline visit was 27 weeks (range 11-52.8). Five patients had potential erosive changes on baseline ultrasound scans, most commonly identified in the ankles, wrists and 2^nd^ MCPs. Improvements in USAJC#1 and USAJC#2 were positively associated with improvements in cJADAS (r = 0.55, p = 0.02; r = 0.45, p = 0.045). Reductions in pathologic lesion size in the wrists (both mid-carpal and radio-carpal recesses) correlated strongly with improvements in cJADAS. (Table 3). Improvement in USAJC#1, but not USAJC#2, was strongly correlated with reduction in ESR (r = 0.71, p = 0.001) and moderately correlated with reduction in CHAQ scores (r = .51, p = 0.03).

**Conclusions**

Objective measures of joint activity by MSUS can feasibly be incorporated into a longitudinal “treat-to-target” study in polyarticular JIA. In this small pilot sample, active joint count by MSUS correlated with measures of clinical disease activity. While hyperemia on MSUS is a primary indicator of inflammation in RA, the significance in JIA is less clear. Future studies will examine these correlations more extensively with a larger cohort and further delineate the role for MSUS in a treat-to-target management strategy in JIA. This study was supported with CARRA-AF Funding.

**Table 1:** **(abstract P9).** Two Definitions for Ultrasound Active Joint Count

USAJC #1	Number of joints with evidence for any component of MSUS pathology (Synovial thickening, Joint Effusion or Hyperemia) > 1+ in any recess.
USAJC #2	Number of joints with evidence for Synovial Thickening (> 1+) with Hyperemia (> 1+) with or without Joint Effusion.

**Table 2:** **(abstract P9).** Baseline Characteristics of Polyarticular JIA Cohort

Mean Age, yrs (range)	9.75 (3.5-17)
Gender (% female)	5 (83%)
Race (% white)	5 (83%)
Ethnicity (% Hispanic)	3 (50%)
Mean Disease Duration, wks (range)	27 (11-52.8)
ANA (% positive)	6 (100%)
RF (% positive)	2 (33%)
CCP (% positive)	3 (50%)
Mean cJADAS (range)	16 (11-22)
Mean ESR (range)	41.5 (14-80)
Mean MD Global (range)	3 (2-4)
Mean Pt/Parent Global (range)	4.5 (0-8)
Mean CHAQ (range)	1.48 (0.5-2.5)
Mean CAJC (range)	11.17 (5-18)
Mean USAJC#1 (range)	6.00 (5-8)
Mean USAJC#2 (range)	1.33 (0-4)

**Table 3:** **(abstract P9).** Correlations between changes in the Wrist Pathologic Lesion size and cJADAS

TAP-RW-MC	0.660	0.003
TAP-LW-MC	0.745	0.000
TAP-RW-RC	0.789	0.000
TAP-LW-RC	0.672	0.002

(TAP-RW-MC = Total area of Pathology-Right Wrist- Mid-Carpal Recess; TAP-LW-MC = Total area of Pathology-Left Wrist- Mid-Carpal Recess; TAP-RW-RC = Total area of Pathology-Right Wrist- Radio-Carpal Recess; TAP-LW-RC = Total area of Pathology-Left Wrist- Radio-Carpal Recess).

**References**

1. Backhus TM et al. The US7 score is sensitive to change in a large cohort of patients with rheumatoid arthritis over 12 months of therapy. Annals of Rheumatic Diseases 2013;72:1163-1169.

2. Harman H et al. Improvement of large-joint ultrasonographic synovitis is delayed in patients with newly diagnosed rheumatoid arthritis: results of a 12-month clinical and ultrasonographic follow-up of a local cohort. Clinical Rheumatology 2015;34(8):1367-74.

## P10 Musculoskeletal ultrasound study in childhood arthritis a limited examination

### Patricia Vega-Fernandez^1^, Tracy V. Ting^2^, Edward J. Oberle^3^, Johannes Roth^4^, for the CARRA Investigators

#### ^1^Emory University, Atlanta, GA, USA; ^2^Cincinnati Children's Hospital Medical Center, Cincinnati, OH, USA; ^3^Nationwide Children’s Hospital, Columbus, OH, USA; ^4^University of Ottawa, Ontario, Canada

**Background**

Juvenile Idiopathic Arthritis (JIA) is the most common rheumatologic disease of childhood. Most of the core set of assessment measurements are subjective by nature. A validated, accessible tool that can evaluate disease response is lacking. Musculoskeletal Ultrasound (MSUS) is a non-invasive, efficient, well accepted imaging tool capable of being used at the bedside by a trained ultrasonographer for the assessment of inflammatory arthritis. MSUS is known to have better sensitivity and reliability to detect synovitis than clinical examination. The aims of this study are 1. Determine the minimum number of joints needed to assess MSUS-evidenced disease activity in JIA; 2. Evaluate feasibility and acceptability from both patient/parent and clinician experience of a limited MSUS.

**Methods**

Newly diagnosed JIA patients presenting with active joint count >4 without recent intraarticular corticosteroid injection in the past month, able to perform first visit within 4 week of starting a Disease-modifying Antirheumatic Drug (DMARD) are eligible for this study. A total of 30 pts will be enrolled for this pilot study. In addition to general demographic and clinical data, a comprehensive clinical physical examination and a 44 joint MSUS examination by an American College of Rheumatology Musculoskeletal Ultrasound (ACR RhMSUS) certified pediatric rheumatologist will be performed at baseline and at 3 months. Both gray-scale B mode and power Doppler images will be obtained for each view. Determination of a limited joint examination will be made by a data reduction process to detect at least 90% synovitis within the comprehensive exam.

**Results**

For a 44 joints MSUS examination a total of 247 views per patient were identified. To assure standardization during the process of imaging collection and scoring the authors have developed an Image Acquisition Manual and an Image Scoring Manual. A calibration exercise addressing imaging acquisition, scoring, and feasibility is underway. Preliminary results revealed time to scan is close to 120-150 min/per patient per session, therefore, based on available literature, authors are discussing possible modifications to the included views. Concurrently, a practical exercise for assessment of interrater reliability is ongoing. Authors have identified the need of a database that support storage and visualization of collected DICOM images that meets IRB standards and allows multisite scoring process; this database is being developed.

**Conclusions**

A limited MSUS examination will help determine the role of MSUS as a diagnostic and prognostic instrument in pediatrics. It will improve clinical assessment of disease activity in JIA and strengthen medical decision making.

**Ethics Approval**

This study was approved by the Institutional Review Board at Emory University, Cincinnati Children’s Hospital Medical Center, and Nationwide Children’s Hospital. IRB approval is pending at the University of Ottawa.

**Funding**

This work is supported by a CARRA-AF Small Grant.

## P11 Subclinical synovitis in oligoarticular JIA: a risk factor for Polyarticular Disease?

### Deirdre De Ranieri^1^, Johannes Roth^2^, Edward Oberle^3^

#### ^1^University of Chicago Medicine, Comer Children’s Hospital; ^2^University of Ottawa, Ottawa, Canada; ^3^Nationwide Children's Hospital, Columbus OH

**Background**

Oligoarticular JIA is associated with a better prognosis than the other subtypes, with a higher percentage of patients achieving remission off medication (1). Polyarticular JIA, on the other hand, is associated with poorer functional outcome (2). Musculoskeletal US is being used increasingly more in the field of rheumatology to evaluate joints and tendons. In RA, it has been shown that that patients in clinical remission can have evidence of subclinical disease on ultrasound, and this finding predicted disease progression in these patients (3). We know that patients with JIA are often re-classified later in their disease course, i.e. from an oligoarticular subtype to a more extended or polyarticular subtype (4), and these subtypes are associated with greater disability and decreased quality of life for children (5). Furthermore, involvement of certain “high risk” joints in JIA (i.e. ankles and wrists) early in the disease course increases the likelihood that a patient will progress to polyarticular disease (6). Determining if these patients are at risk for disease progression would be valuable in terms of initiating more aggressive therapy earlier, as the likelihood of achieving inactive disease is higher the earlier treatment is initiated (7). Indications for biologic medications, such as TNF-inhibitors, are currently restricted to patients with “Polyarticular” JIA. This is a pilot study to evaluate the presence and significance of subclinical synovitis in a cohort of patients with newly diagnosed Oligarticular JIA.

**Methods**

We plan to enroll 40 patients, M and F, ages 2-16 yrs with recently (<12mos) diagnosed Oligoarticular JIA. Bilateral elbow, ankle, wrist and knee joints will be assessed clinically and sonographically at time point 0,3,6,9,12 mos for signs of synovitis. US evaluation will be according to OMERACT guidelines, using a GE Logiq e portable US machine. Physical exam will occur prior to the US. Baseline information such as age, sex, ANA status, +/-uveitis, medications will be obtained. Outcome measures screened at each visit include JADAS, Patient and Physician Global Assessments, ESR and CRP, Joint count (both by clinical and sonographic assessment), PQRL, and patient’s perceived joint count.

**Statistics**

With 40 patients, we will be able to estimate proportions (e.g., the proportion of children who develop clinical disease within a year, the proportion of children with at least one joint having a 1+ on ultrasound at baseline) with sufficient precision so that the half-width of the 95% CI would be no larger than +/-0.16. We will have 80% power to detect a correlation of 0.42-0.43 between change in CRP and US changes over time, for example. To evaluate the relationship between baseline US findings and clinical disease development, assuming that 50% of children will have at least one joint having an effusion on ultrasound at baseline and overall about 30% of children will develop clinical disease within the following year, we’d have 80% power to detect a difference between those with an effusion vs. those without of 10% vs. 49% in the percentage of those developing clinical disease.

**Results and Conclusions**

To be determined.

**Funding**

The authors wish to acknowledge CARRA, and the ongoing Arthritis Foundation financial support of CARRA.

**References**

1. Nordal E, Zak M, Aalto K, Berntson L, Fasth A, Herlin T, et al. Ongoing disease activity and changing categories in a long-term nordic cohort study of juvenile idiopathic arthritis. Arthritis and rheumatism. 2011 Sep;63(9):2809-18. PubMed PMID: 21560116.

2. Packham JC, Hall MA, Pimm TJ. Long-term follow-up of 246 adults with juvenile idiopathic arthritis: predictive factors for mood and pain. Rheumatology. 2002 Dec;41(12):1444-9. PubMed PMID: 12468828.

3. Saleem B, Brown AK, Quinn M, Karim Z, Hensor EM, Conaghan P, et al. Can flare be predicted in DMARD treated RA patients in remission, and is it important? A cohort study. Annals of the rheumatic diseases. 2012 Aug;71(8):1316-21. PubMed PMID: 22294638.

4. Al-Matar MJ, Petty RE, Tucker LB, Malleson PN, Schroeder ML, Cabral DA. The early pattern of joint involvement predicts disease progression in children with oligoarticular (pauciarticular) juvenile rheumatoid arthritis. Arthritis and rheumatism. 2002 Oct;46(10):2708-15. PubMed PMID: 12384930.

5. Ringold S, Wallace CA, Rivara FP. Health-related quality of life, physical function, fatigue, and disease activity in children with established polyarticular juvenile idiopathic arthritis. The Journal of rheumatology. 2009 Jun;36(6):1330-6. PubMed PMID: 19411394.

6. Hemke R, Nusman CM, van der Heijde DM, Doria AS, Kuijpers TW, Maas M, et al. Frequency of joint involvement in juvenile idiopathic arthritis during a 5-year follow-up of newly diagnosed patients: implications for MR imaging as outcome measure. Rheumatology international. 2015 Feb;35(2):351-7. PubMed PMID: 25119829.

7. Wallace CA, Ringold S, Bohnsack J, Spalding SJ, Brunner HI, Milojevic D, et al. Extension study of participants from the trial of early aggressive therapy in juvenile idiopathic arthritis. The Journal of rheumatology. 2014 Dec;41(12):2459-65. PubMed PMID: 25179849.

8. Roth J, Ravagnani V, Backhaus M, Balint P, Bruns A, Bruyn G, Collado P, de la Cruz L, Guillame-Czitron S, Herlin T, Hernandez C, Iagnocco A, Jousse-Joulin S, Lanni S, Lilleby V, Malattia C, Magni-Manzoni S, Modesto C, Narrodi A, Nieto JC, Ohrndorf S, Rossi L, Selvaag AM, Swen N, Ting T, Tzaribachev N, Vega-Fernandez P, Vojinovic J, Windshall D, D’Agostino MA, Naredo E for the Omeract Ultrasound Group. Preliminary Definitions for the Sonographic Features of Synovitis in Children. Arthritis Care & Research. 2016 Oct; PMID: 27748074 [epub ahead of print].

## P12 Longitudinal Musculoskeletal Ultrasound in Juvenile Idiopathic Arthritis

### Leandra U. Woolnough^1,2^, Heather Benham^2^, Yassine Kanaan^2^, David Wilkes^2^, Tracey Wright^1,2^, Marilynn Punaro^1,2^

#### ^1^UT Southwestern Medical Center, Dallas TX, USA; ^2^Texas Scottish Rite Hospital for Children, Dallas, TX, USA

**Background**

Musculoskeletal (MSK) ultrasound (US) is a non-invasive technique that detects joint inflammation. Inflammation detected by ultrasound has been shown to predict disease course in adults with rheumatoid arthritis [1]. Preliminary definitions for MSK US in healthy joints and synovitis have been established for children [2, 3]. Currently, there is no unified approach for identifying early or subclinical joint inflammation in Juvenile Idiopathic Arthritis (JIA). The objective of this study is to determine the diagnostic value of MSK US in JIA.

**Methods**

Aim 1 will distinguish features of MSK US in JIA patients from normal findings in healthy children. We will perform clinical examinations as well as MSK US assessments in JIA and age-gender matched controls (Table 1). We will compare MSK US findings JIA patients and controls using a semi-quantitative scale[4], depth of pathology (mm), and volume (mm^3^). Aim 2 will discern the features of active disease from inactive disease by following newly diagnosed JIA patients prospectively over a 1-year period with serial MSK US and clinical assessments. Utilizing a linear mixed models approach, we will determine if a MSK US joint severity score of inflammation distinguishes JIA patients with persistent clinical disease activity from those with clinically inactive disease [5].

**Results**

Over the past year we have successfully enrolled 18 newly diagnosed JIA patients in a prospective observational study using MSK US to assess selected joints at four month intervals. Our preliminary data indicates that 35% of joints in newly diagnosed JIA reveal inflammation by MSK US not detected by clinical exam. The clinical significance of this discrepancy is unknown.

**Conclusions**

This will be the first study of its kind in patients with JIA. This project has the potential to improve the diagnosis and management of JIA patients by identifying objective measures of disease activity supporting early aggressive treatment to decrease long-term disability.

**Ethics Approval**

This study received funding support from the Childhood Arthritis and Rheumatology Research Alliance (CARRA) and the Arthritis Foundation.

**References**

1. Filer A, de Pablo P, Allen G, Nightingale P, Jordan A, Jobanputra P, Bowman S, Buckley CD, Raza K: Utility of ultrasound joint counts in the prediction of rheumatoid arthritis in patients with very early synovitis. *Annals of the rheumatic diseases* 2011, 70(3):500-507.

2. Roth J, Jousse-Joulin S, Magni-Manzoni S, Rodriguez A, Tzaribachev N, Iagnocco A, Naredo E, D'Agostino MA, Collado P: Definitions for the sonographic features of joints in healthy children. *Arthritis care & research* 2015, 67(1):136-142.

3. Roth J, Ravagnani V, Backhaus M, Balint P, Bruns A, Bruyn GA, Collado P, De la Cruz L, Guillaume- Czitrom S, Herlin T *et al*: Preliminary definitions for the sonographic features of synovitis in children. *Arthritis care & research* 2016.

4. Szkudlarek M, Court-Payen M, Jacobsen S, Klarlund M, Thomsen HS, Ostergaard M: Interobserver agreement in ultrasonography of the finger and toe joints in rheumatoid arthritis. *Arthritis and rheumatism* 2003, 48(4):955-962.

5. Wallace CA, Huang B, Bandeira M, Ravelli A, Giannini EH: Patterns of clinical remission in select categories of juvenile idiopathic arthritis. *Arthritis and rheumatism* 2005, 52(11):3554-3562.

**Table 1:** **(abstract P12).** Anatomical Recesses Evaluated by MSK US

Dorsal Wrist	Midcarpal recess Radiocarpal recess
Knee	Suprapatellar recess Medial recess Lateral recess

## P13 Improving care for children with autoinflammatory diseases: CARRA-sponsored network building

### Marinka Twilt^1^, Eyal Muscal^2^, Ana Sepulveda^1^, Rae Yeung^3^, Susanne Benseler^1^

#### ^1^Alberta Children's Hospital, University of Calgary, Calgary, Alberta, Canada; ^2^Baylor College of Medicine, Houston, Texas, USA; ^3^Sickkids, University of Toronto, Toronto, Ontario, Canada

**Background**

Over the last 25 years, our expanding knowledge about childhood diseases presenting with non-infectious, ‘sterile’ inflammatory fever attacks has dramatically increased and significantly changed our approach to treatment. In 1999, the concept of "autoinflammation" was proposed by Dan Kastner and colleagues as the molecular mechanisms other than autoimmunity as cause for known periodic fever syndromes such as FMF and TRAPS. Nowadays, autoinflammatory conditions are described as a genetically heterogeneous group of rheumatologic diseases that are caused by single gene mutations and driven by abnormal activation of the innate immune system. Innate immunity research and discoveries have led to identification of several disease causing genes associated with an excessive cytokine release. These diseases were associated with high mortality and morbidity, however recognition has increased and early targeted treatment can prevent organ damage and increase outcome and quality of life. In most cases, life-long control of ongoing inflammation is necessary in order to improve, stabilize and minimize clinical symptoms and prevent development of damage. To date, however, all of the studies have been focused on well-described disease entities with a genetic underlying mechanism. In addition, many of the studies are based on case-reports or case-studies, which may have led to limitations in identifying the precise molecular pathway.

**Methods**

CARRA small grant support (2017) has funded infra-structure for the improvement of care for children with autoinflammatory diseases (AID) through development of a CARRA autoinflammatory registry and develop CTPs for the treatment of CAPS in the autoinflammatory registry. Funds were provided for the development of the autoinflammatory network and the CTPs for CAPS. CARRA funds have supported a portion of a research assistant at the Alberta Children’s Hospital since September 2017. Research assistant responsibility included: assistance in development of autoinflammatory registry data fields, set-up meetings, and literature search and systematic review for CAPS CTP.

**Results**

The CARRA autoinflammatory subcommittee has reached out to the CARRA PFAPA and CARRA CRMO group to develop one autoinflammatory registry including all diseases. A first iteration of data-elements has taken place and another is planned for the 2018 CARRA meeting. The aim is to reach consensus on the mandatory data elements during the 2018 CARRA meeting. The CTP project is currently in progress. A search has been performed and 432 papers were identified. Title and abstract selection have been performed and we are currently in the process of full-text analysis. The results are expected to be available during the 2018 CARRA meeting to be discussed in the autoinflammatory network.

**Conclusion**

Due to the CARRA small grant the CARRA autoinflammatory subcommittee was able to establish an autoinflammatory network including the CARRA working groups that deal with AID. The network has been able to pursue the establishment of an autoinflammatory registry and the systematic review to establish a CTP for CAPS. Continued collaboration with all partners is necessary to improve the care of children with AID.

**Funding**

CARRA small grants/Arthritis Foundation.

## P14 Continuous temperature monitoring to improve diagnosis and treatment of PFAPA

### Jonathan S. Hausmann^1,2^, Kalpana Manthiram^3^, Edwin Anderson^1^, Sivia Lapidus^4^, Fatma Dedeoglu^1^, for the CARRA PFAPA Subcommittee

#### ^1^Boston Children's Hospital, Boston, MA, USA; ^2^Beth Israel Deaconess Medical Center, Boston, MA, USA; ^3^National Institutes of Health, Bethesda, MD, USA; ^4^Goryeb Children's Hospital, Morristown, NJ, USA

##### **Correspondence:** Jonathan S. Hausmann

**Background**

PFAPA (periodic fevers, aphthous stomatitis, pharyngitis, and adenitis) is the most common autoinflammatory disease in children. It is characterized by fevers lasting 3-5 days and recurring “like clockwork” every 3-6 weeks, with the presence of the features of the disease name. Between episodes, children are well and demonstrate normal growth and development.

One of the challenges of diagnosing PFAPA is distinguishing fever episodes from those of recurrent viral illnesses. As a result, most children with PFAPA have delays in diagnosis on the order of months to years (Hofer et al. 2014).

Our study seeks to leverage continuous temperature monitoring to determine fever patterns in patients with PFAPA, with the hopes of improving diagnosis and facilitating treatment.

**Methods**

Children with PFAPA, as determined by their CARRA-affiliated rheumatologist, will be eligible to enroll. The PFAPA Consensus Treatment Plans (CTP) diagnostic criteria will be used. Families will be provided with an informational brochure about our study, including links to our study website: PFAPAstudy.com. Families interested in participating will contact the study team at Boston Children’s Hospital and will be consented over the phone.

Families will be provided with an iThermonitor, an FDA-approved device that records continuous body temperature and syncs to a smartphone. Children will be asked to wear the iThermonitor for a few days before, during, and after a PFAPA flare. Temperature data will be sent automatically and securely to an online dashboard accessible to study investigators.

Families will complete an online health survey and a fever diary based on the Autoinflammatory Disease Activity Index (Piram et al., 2014), in which they will record symptoms and timing of medication administration.

**Results**

Recruitment of participants is pending IRB approval.

**Conclusions**

We believe that these fever patterns are unique to the pathophysiology of PFAPA and may help to provide rapid and accurate diagnoses of children with this syndrome. Also, use of continuous temperature monitoring could improve earlier recognition of febrile episodes, facilitate prompt administration of steroids, and result in increased efficacy of treatment. Finally, continuous temperature monitoring may provide objective data about the relative efficacy of different treatment options for PFAPA, which could be useful when conducting comparative effectiveness research studies such as in the PFAPA CTP.

**References**

Hofer M, et al. (2014). International periodic fever, aphthous stomatitis, pharyngitis, cervical adenitis syndrome cohort: description of distinct phenotypes in 301 patients. Rheumatology (Oxford) 53:1125–1129.

Piram, M., et al. (2014). Validation of the Auto-Inflammatory Diseases Activity Index (AIDAI) for hereditary recurrent fever syndromes. Annals of the Rheumatic Diseases, 73(12), 2168–2173.

**Ethics Approval**

The study was approved by the Institutional Review Board at Boston Children’s Hospital.

**Funding**

This study is funded through a CARRA-Arthritis Foundation Grant.

## P15 The impact of psychiatric comorbidity on health care utilization for youth with SLE

### Andrea Knight^1,2,3^, Alaina Davis^4^, Marisa Klein-Gitelman^5^, Jennifer Faerber^6^, Hannah Katcoff^6^, Zuleyha Cidav^1,7^, David S. Mandell^1,7^

#### ^1^Perelman School of Medicine, University of Pennsylvania, PA, USA; ^2^Center for Pediatric Clinical Effectiveness, The Children's Hospital of Philadelphia, PA, USA; ^3^PolicyLab, The Children's Hospital of Philadelphia, PA, USA; ^4^Division of Pediatric Rheumatology, Monroe Carell Junior Children's Hospital at Vanderbilt, TN, USA; ^5^Division of Pediatric Rheumatology, Ann & Robert H Lurie Children’s Hospital of Chicago, IL, USA; ^6^Health Analytics Unit, The Children's Hospital of Philadelphia, PA, USA; ^7^Center for Mental Health Policy and Services Research, University of Pennsylvania, PA USA

**Background**

Youth with systemic lupus erythematosus (SLE) have high health care utilization, which may be exacerbated by psychiatric comorbidity. We examined the impact of psychiatric diagnoses on utilization of medical services in youth with SLE.

**Methods**

We conducted a retrospective cohort study using administrative claims for 2000 to 2013 from Clinformatics^TM^ DataMart (OptumInsight, Eden Prairie, MN), a large US database of privately insured enrollees. We included youth ages 10-24 years with an incident diagnosis of SLE (≥3 International Classification of Diseases, Ninth Revision codes for SLE 710.0, each >30 days apart, with > 1 year of preceding continuous enrollment without a code for SLE). We categorized mutually exclusive groups of youth with SLE as those with: 1) no psychiatric diagnosis, 2) a psychiatric diagnosis in the 12 months preceding SLE diagnosis, and 3) an incident psychiatric diagnosis in the 12 months after SLE diagnosis. We calculated mean ambulatory, emergency and inpatient visits for medical (non-psychiatric) services in the year after SLE diagnosis, and used Poisson regression to compare the number of visits among the 3 groups, adjusting for demographic and disease variables.

**Results**

We identified 650 youth with an incident diagnosis of SLE. The mean age was 18.4 years (SD 3.7), 88% were females and 25% had nephritis. Depression was diagnosed in 117 (18%), anxiety in 78 (12%), and other psychiatric disorders in 176 (27%). Psychiatric diagnoses preceding SLE diagnosis were present in 122 (19%), incident after SLE diagnosis in 105 (16%), and absent for 423 (65%). In adjusted models, youth with incident psychiatric diagnoses had higher mean ambulatory visits in the year after SLE diagnosis compared to those with preceding (p<0.01) and without psychiatric diagnoses (p<0.001) (Figure 1). Youth with incident psychiatric diagnoses had more primary care and rheumatology visits than the other groups (Figure 2).

Emergency visits were higher for those with preceding (p<0.01) and incident psychiatric diagnoses (p<0.001), compared to those without (Figure 1). Differences in inpatient visits did not reach statistical significance.

**Conclusion**

Psychiatric comorbidity is prevalent in newly-diagnosed youth with SLE, and associated with higher utilization of medical services in primary care, rheumatology and acute care settings. Interventions to address existing and newly identified psychiatric comorbidity may decrease health care burden for youth with SLE.

**Fig. 1:**
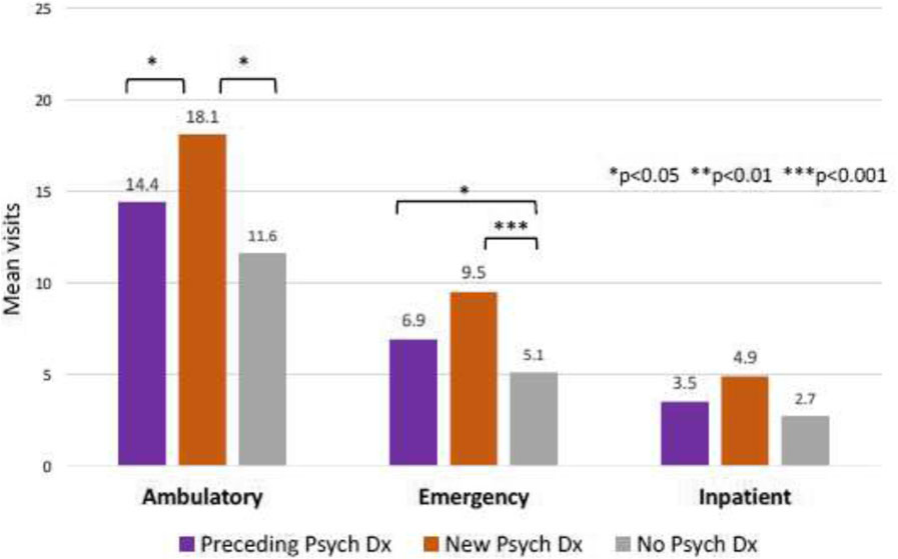
**(abstract P15).** Comparison of Annual Medical Visits by Psychiatric Status for Youth with new-onset SLE

**Fig. 2:**
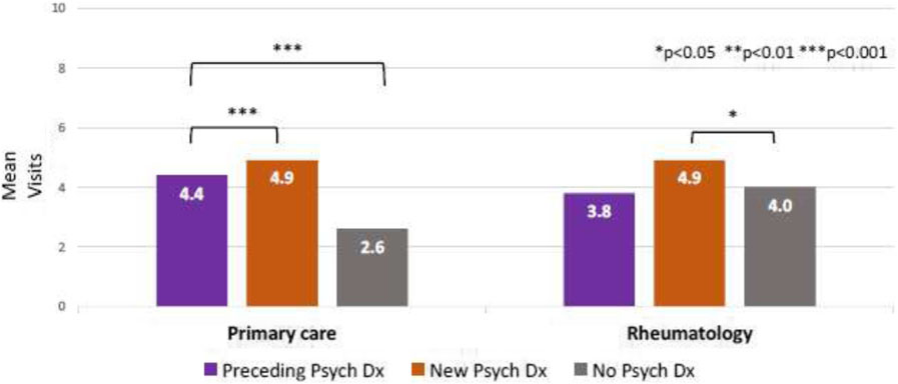
**(abstract P15).** Primary Care and Rheumatology Visits by psychiatric status for youth with new-onset SLE

## P16 Patient and parent acceptability of mental health screening in the rheumatology clinic for youth with Systemic Lupus Erythematosus

### Tamar Rubinstein^1^, Marija Dionizovik-Dimanovski^1^, Raphael Kraus^1^, Jordan T. Jones^2^, Julia Harris^2^, Martha Rodriguez^3^, Melissa Tesher^4^, Lauren Faust^5^, Beth Rutstein^5^, Chelsey Smith^2^, Rebecca Puplava^4^; Enrique Rojas^4^, Alaina Davis^6^, Sangeeta Sule^7^, Karen Onel^8^, Emily von Scheven^9^, Andrea Knight^5^, for the CARRA SLE Subcommittee

#### ^1^Children’s Hospital at Montefiore/Albert Einstein College of Medicine, Bronx, NY, USA; ^2^Children's Mercy Hospitals and Clinics, Kansas City, MO, USA; ^3^Riley Hospital for Children at Indiana University Health, Indianapolis, IN, USA; ^4^University of Chicago Medicine Comer Children's Hospital, Chicago, IL, USA; ^5^Children's Hospital of Philadelphia, Philadelphia, PA, USA; ^6^Vanderbilt University Medical Center, Nashville, TN, USA; ^7^Johns Hopkins Children's Center, Baltimore, MD, USA; ^8^Hospital for Special Surgery, New York, NY, USA; ^9^UCSF Benioff Children's Hospital, San Francisco, CA, USA

**Background/Purpose**

Despite high depression and anxiety prevalence in youth with systemic lupus erythematosus (SLE), standardized mental health screening is not routinely practiced by pediatric rheumatologists. Gaps in knowledge exist around family acceptance of mental health screening in subspecialty clinics. Our objectives were to screen youth with SLE for depression and anxiety in pediatric rheumatology clinics and assess patient and parent acceptability of screening in this clinical setting.

**Methods**

In a multi-center study of collaborating clinics from the Childhood Arthritis and Rheumatology Research Alliance, 12-21 year olds with SLE were consecutively screened with the Patient Health Questionaire-9 (PHQ-9) for depression and the Generalized Anxiety Disorder 7-item scale (GAD-7) for anxiety. Patient reported outcomes (PROs) were collected and follow up surveys were given to patients and/or parents.

**Results**

From the first 4 sites, 53 patients were screened and follow up surveys were completed for 74%. The median age was 16.2 years, 87% were female, and 75% were of minority race/ethnicity. Of those screened, 26% screened positive for depression, 19% for anxiety, and 15% endorsed suicidal ideation. Most patients reported being previously screened, 17% by their general pediatricians; 15% were newly identified as having symptoms of depression or anxiety. PHQ-9 scores highly correlated to PROs for fatigue; GAD-7 scores were highly correlated to fatigue and pain interference. In follow up surveys, 95% of patients/parents thought that emotional health was an important part of general health, 91% felt comfortable being screened by their rheumatologist, and 74% felt they should continue to be routinely screened by their rheumatologist (Figure 1).

**Conclusions**

While the importance of routine standardized screening for mental health is well recognized in general pediatric practices, the role of mental health screening in pediatric rheumatology clinics has not been established. The preliminary results of this study show that screens picked up new depression/ anxiety cases and a high prevalence of suicidal ideation. Depression and anxiety screens correlated with PROs, particularly for fatigue and pain. Most patients and parents surveyed felt mental health was an important aspect of general health and supported screening by their pediatric rheumatologist. This data support mental health screening in pediatric rheumatology clinics as an important and acceptable practice to patients and families across diverse demographics.

**Ethics Approval**

This study was approved by the Albert Einstein College of Medicine IRB, the University of Chicago Biological Sciences Division IRB, the Children’s Hospital of Philadelphia Research Institute IRB, and Children’s Mercy Hospital Pediatric IRB.

**Funding**

This study was funded by the CARRA-Arthritis Foundation Small Grant Award.

**Fig. 1:**
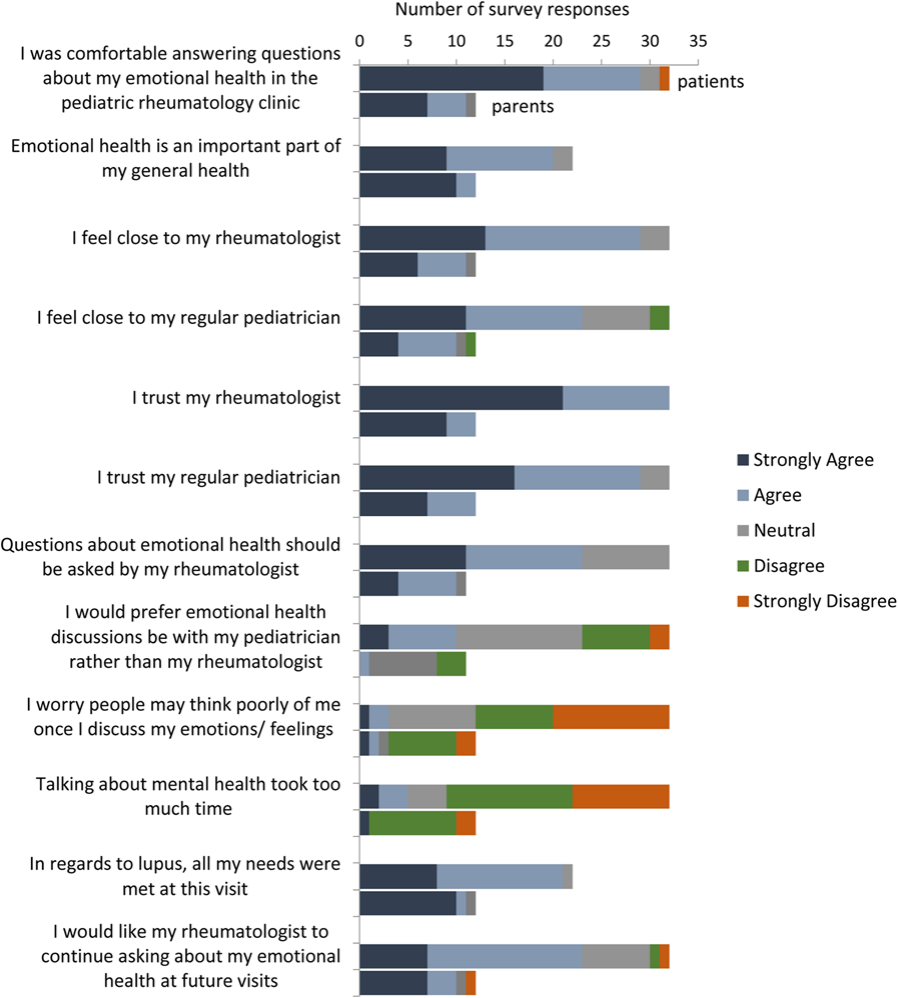
**(abstract P16).** Survey responses from patients with SLE and parents after mental health screenings in the rheumatology clinic

## P17 Maintenance therapy discontinuation of mycophenolate mofetil in pediatric proliferative lupus nephritis: a multi-centered retrospective cohort study

### Sherene Mason^1^, Marietta DeGuzman^2^, Melinda Carpenter^1^, Jerome C. Lane^3^, Samriti Dogra^1^, for the CARRA SLE subcommittee

#### ^1^Connecticut Children’s Medical Center, Hartford, CT, USA; ^2^Texas Children’s Hospital, Houston, TX, USA; ^3^Lurie Children’s Hospital, Chicago, IL, USA

##### **Correspondence:** Sherene Mason

**Background**

In childhood-onset systemic lupus erythematosus (cSLE) with proliferative lupus nephritis, mycophenolate mofetil (MMF) has been the most commonly used maintenance steroid sparing and immunomodulatory therapy. There is a great need for information on when lupus patients can safely discontinue major immunomodulatory therapy after a period of disease quiescence. Currently the “stopping point” for such treatment is unknown. On the background of the chronic and episodic course of SLE, which at times could be unpredictable, it is likely that significant numbers of cSLE patients may tolerate discontinuation of MMF without clinical and serologic flare. With hydroxychloroquine use, sustained disease remission after MMF discontinuation has been observed. To date, there have been no defined algorithms or guidelines as to the optimal duration of maintenance MMF therapy, both in the adult and childhood SLE population.

A team of pediatric rheumatologists and nephrologists was established to determine the feasibility of conducting a retrospective chart review across two large research consortiums, Childhood Arthritis and Rheumatology Research Alliance (CARRA) and the Midwest Pediatric Nephrology Consortium (MWPNC).

**Methods**

All members of 2 research organizations, CARRA and the 70 participating MWPNC medical centers, were sent a short survey through Survey Monkey. Respondents were queried on their specialty, institution and contact information, numbers of pediatric patients with proliferative lupus nephritis at their institution who have been discontinued off MMF due to prolonged remission (defined as 6 months of clinical remission), and interest in participating in a retrospective chart review on MMF discontinuation in pediatric lupus nephritis. Findings were used to design the study protocol which has now been approved by both CARRA and the MWPNC, with Connecticut Children’s Medical Center serving as the lead coordinating center.

**Results:**

There were 146 respondents to the survey, 24% of whom were identified as pediatric nephrologists and 76% as pediatric rheumatologists. Ninety-five respondents (65%) from 59 centers expressed an interest in participating in the retrospective chart review (Table 1). Over one-third of respondents (36%) reported treating 5-10 pediatric proliferative lupus nephritis patients with MMF (Figure 1). The median number of patients reported per center with proliferative lupus nephritis that have been discontinued off MMF due to prolonged remission was 2 (IQR = 0-4.5).

**Conclusions**

As of January 2018, startup packages were sent to 44 centers, some of which have already joined the study. Eleven centers have declined to participate, citing no research support, lack of patients meeting inclusion criteria, or for undisclosed reasons.

**Ethics Approval**

This study has been approved by the Connecticut Children’s Medical Center’s IRB.

**Table 1:** **(abstract P17).** Survey Results

Unique centers interested in participating	
USA	50
Canada	4
Outside USA/Canada	5

**Fig. 1:**
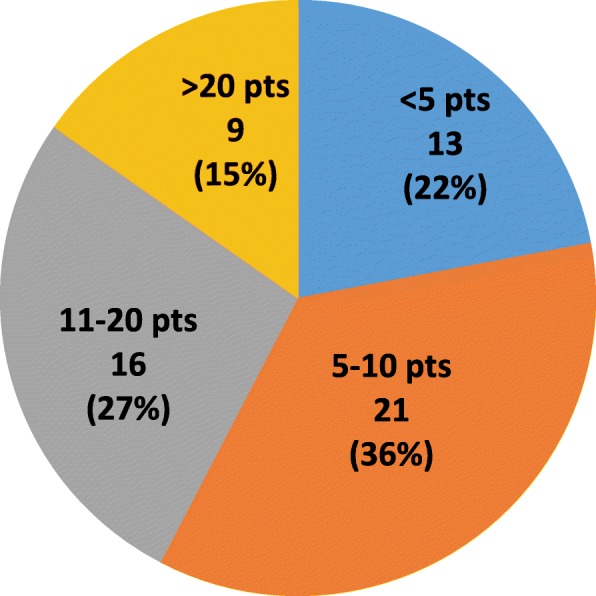
**(abstract P17).** Number of patients per center with proliferative lupus nephritis on MMF maintenance therapy

## P18 The relationship between changes in adipokine levels and disease activity in pubertal children with SLE

### Martha Rodriguez, Kathleen M. O’Neil

#### Riley Hospital for Children at Indiana University Health

**Background**

Systemic lupus erythematosus (SLE) is 3-4 fold more common in girls than boys. Female predominance increases after puberty (9:1). This study focuses on contributions individual hormones and adipokines provide to sexual dichotomy in SLE, the pubertal rise in disease activity, and by proxy, the increased incidence of lupus during puberty. We are using an existing database and serum bank to find associations between disease activity and both physical markers of pubertal progress and hormone/adipokine changes of sexual maturation.

**Methods**

We have a comprehensive database including serial history and examination data, Tanner stages, laboratory assessments and SLEDAI-2K disease activity scores, paired with sera (for hormone/adipokine measurement) to identify associations between disease activity and flares/improvements, and hormonal changes. We are extending our preliminary observations from the first 38 girls completing the study, examining the remaining 31 subjects (10 boys and 21 girls). The database/serum bank represents 59 girls and 10 boys with SLE enrolled in early puberty (PreP, girls 8-13 years old, boys 9-15) from 10 CARRA sites followed through puberty. 3-monthly assessments continued until 1 year post-menarche (girls) or post Tanner IV (boys) or study end. Subjects and parents completed CHAQ (Childhood Health Assessment Questionnaire) questionnaires and the treating MD completed SLICC Damage Measure questionnaires biannually. Serum hormone and adipokine concentrations are measured using commercial assays (EMD Millipore). Analysis includes first univariate, then multivariate associations using generalized equalizing equations (GEE) to correlate SLEDAI scores and hormonal/adipokine concentrations. A database from girls with SLE beginning after age 15 is the post-pubertal SLE controls (PostP).

**Results**

The average participation was 21 months**.** Of 55 girls with PreP lupus, 100% had ds-DNA antibody vs 70% in PostP cohort (p=0.001). PreP were twice as likely to have anemia (37 vs 18%, p=0.01) as PostP. PreP had more nephritis at onset (60 vs 40%, p<0.02). Serum FSH, LH and estradiol did not correlate temporally with flare. In 270 visits 48 represented mild-moderate flares (MMF, 17.8%) and 19 were severe flares (SvF, 7.04%); 24.8% were any flare. SLEDAI decreased >3 points in 23.3% of visits. 49% of visits had sufficient change in SLEDAI to consider changing treatment. Flares increased in Tanner stages 2 and 3 vs stage 1 (p=0.008). No SvF occurred in Tanner 4 or later. Flares correlated with rapid falls in visfatin (OR 0.072, p=0.002) and high adiponectin (OR 1.20, p=0.0046). Neither estradiol nor testosterone correlated with flare. Improvement correlated with testosterone (p=0.023) and followed falls in adiponectin (0.008) and rises in prolactin (p=0.003) and resistin (p=0.026).

**Conclusion**

Preliminary results suggest a high rate of SLE flare in PreP SLE during puberty. Adiponectin correlates positively with SLE flare and testosterone with improvement. This study should elucidate mechanisms of pubertal SLE activation and may identify pathways for intervention.

**Funding**

NIAMS R03AR52453, NIAID R56AI085258, LFA Pediatric Grant, CARRA 2018 grants.

## P19 Evaluation of the reliability and validity of the Cutaneous Lupus Erythematosus Disease Area and Severity Index (CLASI) in pediatrics

### C.J. Kushner^1,2^*, M. Tarazi^1,2^*, R. Gaffney^1,2^, R. Feng^1^, K. Ardalan^3^, H. Brandling-Bennett^4^, L. Castello-Soccio ^5^, J.C. Chang^6^, Y.E. Chiu^7^, S. Gmuca^6^, R.D. Hunt^8^, P. Kahn^9^, A.M. Knight^6^, J. Mehta^6^, D. Pearson^1,2^, J.R. Treat^5^, J. Wan^5^, A. Yeguez^1^, J.S.S. Concha^1,2^, B. Patel^1,2^, J. Okawa^1,2^, L.M. Arkin^10^, V.P. Werth^1,2^

#### ^1^Department of Dermatology, University of Pennsylvania, Philadelphia, PA; ^2^Corporal Michael J. Crescenz VAMC, Philadelphia, PA; ^3^Departments of Pediatrics and Medical Social Sciences, Northwestern University Feinberg School of Medicine; ^4^Department of Pediatrics, University of Washington School of Medicine, Seattle, WA; ^5^Pediatric Dermatology, Children’s Hospital of Philadelphia, Philadelphia, PA; ^6^Pediatric Rheumatology, Children’s Hospital of Philadelphia, Philadelphia, PA; ^7^Departments of Pediatrics and Dermatology, Medical College of Wisconsin, Milwaukee, WI; ^8^Department of Dermatology and Pediatrics, Texas Children’s Hospital, Baylor College of Medicine, Houston, TX; ^9^Division of Pediatric Rheumatology, New York University School of Medicine, New York, NY; ^10^Departments of Dermatology and Pediatrics, University of Wisconsin School of Medicine, Madison, WI

*Authors contributed equally

Cutaneous lupus erythematosus (CLE) refers to skin manifestations of the autoimmune disease lupus erythematosus (LE). Skin involvement is one of the most common presenting signs of systemic lupus erythematosus (SLE) and its evaluation can be critical in making a diagnosis and monitoring disease progression. Patients may also present with isolated CLE without systemic disease. The visible lesions of CLE can be disfiguring and distressing to patients. While CLE has been extensively researched in the adult population, few studies exist in the pediatric population. The development of a validated disease severity tool is crucial for monitoring the natural history of skin involvement in lupus registry studies. There is also a great need for new therapeutic agents, and demonstrating the efficacy of these agents will require clinical trials with reliable outcome measures. The CLASI is a reliable outcome measure for CLE in the adult population, where it is commonly used in clinical trials for SLE. However, no study has validated this assessment tool in children, potentially limiting the conduct of clinical trials in pediatric SLE.

The study will include at least five pediatric dermatologists and five rheumatologists to independently evaluate patients with pediatric cutaneous lupus. The study will take place at the autoimmune disease clinic of the University of Pennsylvania, on March 3^rd^, 2018. The physicians will be given a training session on the assessment of cutaneous lupus using 2 measurement tools: the CLASI and the Physician Global Assessment (PGA), which allow grading of skin activity and skin damage, as well as a score for the overall findings. One cohort of physicians will apply the PGA before the CLASI and the other cohort will apply the CLASI before the PGA. Inter-rater reliability within each physician group will be determined by intraclass correlation coefficient (ICC), type ICC and its confidence interval.

While many pediatric dermatologists and rheumatologists have shown strong interest in participation, there have been difficulties recruiting pediatric CLE patients with currently active disease who are willing to travel to Philadelphia. However we have reached out to over ten institutions and all physicians are actively searching for interested patients and we have identified several patients who will participate.

The primary benefit of this study is the validation of a standardized instrument that can be applied to pediatric cutaneous lupus to facilitate epidemiologic studies and provide a critical tool for clinical trials. Secondary benefits include more standardized documentation of skin disease activity and damage between specialties to help facilitate clinical practice and interdisciplinary collaboration.

**Ethics Approval**

This study has been approved by the University of Pennsylvania IRB.

**Funding**

This project was supported by a CARRA-AF grant.

## P20 Enhancing care for children with Autoimmune Encephalitis (AE): CARRA-sponsored multi-disciplinary network building

### Eyal Muscal^1^, Heather Van Mater^2^, Elizabeth Wells^3^, Marinka Twilt^4^, Dominic Co^5^, Susanne Benseler^2,5^, for the CARRA inflammatory brain disease subcommittee

#### ^1^Baylor College of Medicine, Houston, TX, USA; ^2^Duke University School of Medicine, Durham, NC, USA; ^3^Children's National Medical Center, Washington, D.C., USA; ^4^University Of Calgary Cumming School of Medicine, Calgary, Alberta, Canada; ^5^University of Wisconsin School of Medicine and Public Health, Madison, WI, USA

**Background**

Autoimmune encephalitis (AE) encompasses a spectrum of immune-mediated brain disorders that cause severe neuropsychiatric manifestations due to autoantibody-driven disruption of normal synaptic communication. AE leads to protracted hospital stays and complex neurocognitive rehabilitation needs and may pose significant psychosocial strains on families and care givers. Optimal care of children with AE requires a multidisciplinary team concept that often includes neurology, rheumatology, psychiatry, rehabilitation, critical care and nursing support. AE treatment protocols have yet to be validated and there is still immense variability in the timing of diagnosis and utilization of immunosuppressive regimens. Under the auspices of the CARRA SVARD sub-committee, the inflammatory brain disease work group is pursuing AE care projects. The work group strives to optimize networking between different pediatric specialties. We describe the work group’s progress and its goals for the upcoming year.

**Methods**

CARRA small grant support (2016 and 2017) has funded infra-structure support for the inflammatory brain disease work group. Travel funds were provided to assist pediatric neurologists in attending recent rheumatology meetings. Pediatric neurologists attended the 2016 CARRA meeting (Toronto) and 2016 ACR meeting (shadow AE meeting hosted by Children’s National Hospital, Pediatric Neurology, Washington, D.C.). CARRA funds have supported a portion of a research coordinator at Texas Children’s Hospital, Houston, TX since June 2017. Research coordinator responsibilities have included: scheduling and recording monthly/bi-monthly group conference calls, and designing/maintaining an AE confluence page (shared site) in the CARRA members’ only site.

**Results**

The inflammatory brain disease group has completed scheduled conference calls and has co-leaders for diagnostic work up, CTP design and outcome measures’ projects. The group has brought in pediatric neurology “champions” to assist with all 3 projects. Additionally, a larger group of pediatric neurologists with exposure to neuro-immunology and AE care are being recruited to complete future CTP and outcome measure surveys. Work group deliverables for 2018 include: a draft of an NMDAR CTP, a multi-specialty vetted diagnostic algorithm, a proposed battery of validated outcome measures to be used in conjunction with CTPs, and an initial proposal for a family-engagement project.

**Conclusions**

Due to CARRA financial support since 2016 the inflammatory brain disease work group has been able to pursue AE care projects. Continued collaboration between pediatric neurologists and rheumatologists will hopefully improve the care of children with AE in North America.

## P21 Engaging patients and parents to improve mental health for youth with rheumatologic disease

### Oluwatunmise Fawole^1^, Michelle Vickery^1^, Lauren Faust^1^, Tamar Rubinstein^2^, Julia Harris^3^, Aimee Hersh^4^, Karen Onel^5^, Erica Lawson^6^, Emily von Scheven^6^, Kaveh Ardalan^7^, Esi Morgan^8^, Anne Paul^8^, Judith Barlin^9^, Paola Daly^9^, Mitali Dave^10^, Shannon Malloy^10^, Shari Hume^10^, Suzanne Schrandt^11^, Laura Marrow^11^, Angela Chapson^1^, Donna Napoli^1^, Michael Napoli^1^, Miranda Moyer^1^, Rachel Adamski^1^, Vincent Delgaizo^12^, Martha Rodriguez^13^, Andrea Knight^1^, for the CARRA Investigators^14^

#### ^1^The Children's Hospital of Philadelphia, Philadelphia, PA, United States; ^2^Children's Hospital at Montefiore, New York, NY, United States; ^3^University of Missouri-Kansas City, Children’s Mercy-Kansas City, Kansas City, MO, United States; ^4^University of Utah, Salt Lake City, UT, United States; ^5^Hospital for Special Surgery, Weill Cornell Medicine, New York, NY, United States; ^6^University of California San Francisco, San Francisco, CA, United States; ^7^Lurie Children’s Hospital of Chicago, Chicago, IL, United States; ^8^Cincinnati Children’s Hospital Medical Center, Cincinnati, OH, United States; ^9^Lupus Foundation of America, Washington, DC, United States; ^10^Cure JM Foundation, Encinitas, CA, United States; ^11^Arthritis Foundation, Saint Paul, MN, United States; ^12^Patient-Centered Outcomes Research Institute, Washington, DC, United States; ^13^Riley Children’s Hospital at Indiana, Indianapolis, IN, United States; ^14^Childhood Arthritis and Rheumatology Research Alliance, Milwaukee, WI, United States.

**Background**

Mental health conditions are common in youth with rheumatologic disease, yet intervention strategies for pediatric subspecialty patients is understudied. Patient-engaged research, involving patients and families on the research team, is a valuable technique for examining sensitive topics such as mental health. We used a patient-engaged approach to investigate mental health needs of youth with rheumatologic disease.

**Methods**

An online survey examined beliefs and experiences with mental health for patients with juvenile arthritis, juvenile dermatomyositis, or systemic lupus erythematous. Youth ages 14-24 years and parents of youth 8-24 years were eligible to participate. The survey was developed in collaboration with patient and parent advisors, the Childhood Arthritis & Rheumatology Research Alliance (CARRA), and the Patients, Advocates, and Rheumatology Teams Network for Research and Service (PARTNERS). Participants were recruited through the Arthritis Foundation, Lupus Foundation of America, and Cure JM Foundation. We compared youth and parent responses using regression models (adjusted for demographic and disease covariates) to examine the prevalence of mental health problems, and Likert ratings for the impact of disease aspects on mental health, and comfort level with potential mental health providers.

**Results**

352 respondents included 93 youth (26%) and 259 (74%) parents. Mental health problems were highly prevalent, with clinician-diagnosed anxiety reported by 45%, depression 32%, and adjustment disorders 26% (Figure 1); another 13%, 21% and 11% reported self- diagnosed symptoms of these disorders, respectively. Mean Likert scores indicated that disease aspects most impacting mental health (0=low, 4=high) were physical limitation at 2.7 (SD 1.1), taking medications at 2.6 (1.2), and dealing with disease flares at 2.5 (1.2). Adjusted models showed no difference between youth and parents for reported mental health problems or impacting factors. Youth were significantly less comfortable interacting with all potential mental health providers than parents, particularly social workers and school counselors (Figure 2); both groups felt most comfortable with rheumatologists and primary care providers.

**Conclusion**

Youth with rheumatologic disease have high rates of diagnosed and undiagnosed mental health problems, which are impacted by their disease. Mental health intervention strategies are needed, focusing on both primary care and subspecialty care settings to provide mental health education, screening and treatment for these youth.

**Ethics Approval**

The study was approved by the Children’s Hospital of Philadelphia IRB, Duke University Health System IRB, and Lupus Foundation of America IRB.

**Fig. 1:**
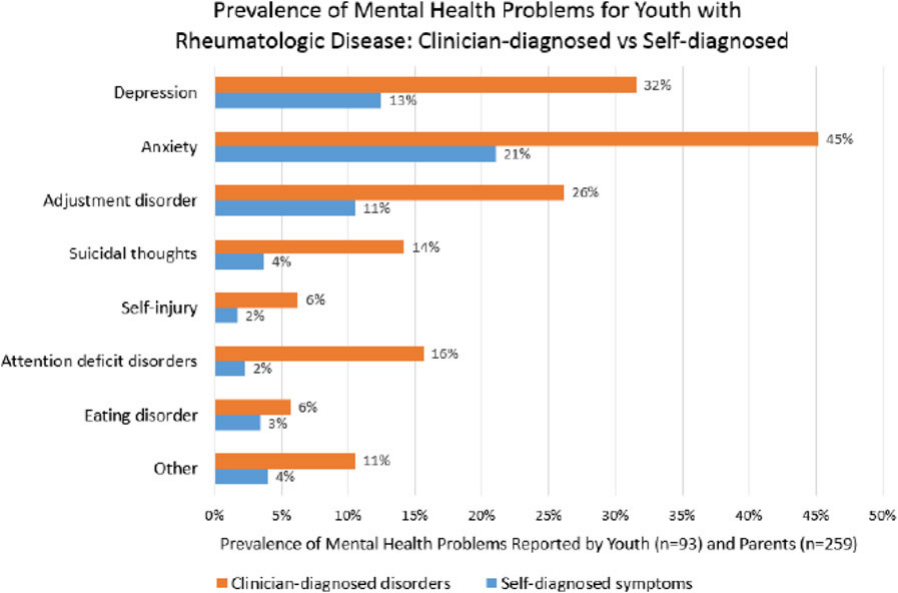
**(abstract P21).** Prevalence of Mental Health Problems for Youth with Rheumatologic Disease: Clinician-diagnosed vs Self-diagnosed

**Fig. 2:**
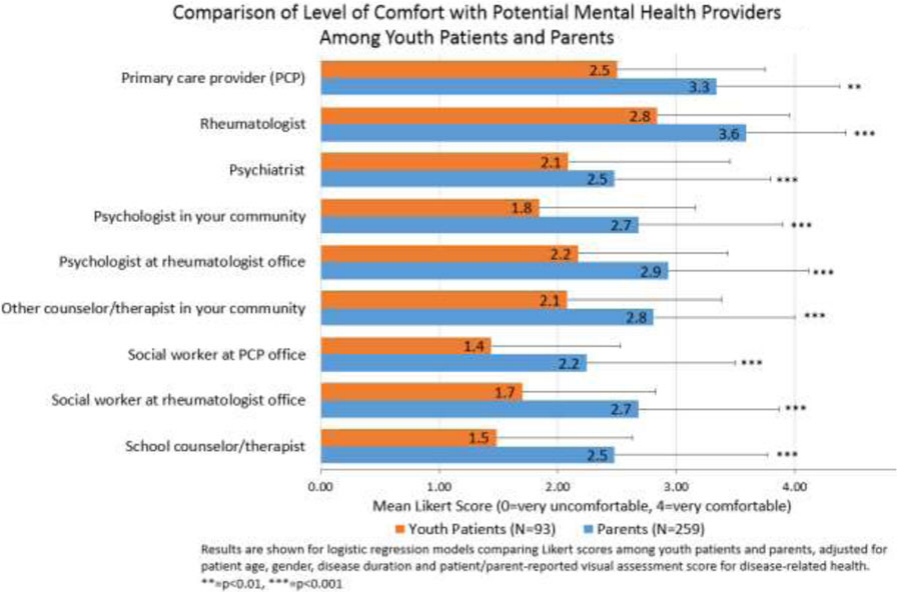
**(abstract P21).** Comparison of Level of Comfort with Potential Mental Health Providers Among Youth Patients and Parents

## P22 Preliminary analyses examining correlates of health self-management

### Anna Richmond^1^, Eyal Muscal^2^, Richard Karl Vehe^3^, Kathleen M. O’Neil^4^

#### ^1^Division of Pediatric Rheumatology, Monroe Carell Junior Children’s Hospital at Vanderbilt, Nashville, TN, USA; ^2^Dept. of Pediatrics, Texas Children’s Hospital, Baylor College of Medicine, Houston, TX, USA; ^3^Division of Rheumatology, Department of Pediatrics, University of Minnesota, Minneapolis, MN, USA; ^4^Rheumatology Division, Indiana University School of Medicine, Indianapolis, IN, USA

**Background**

Although outcomes among youth with lupus have improved significantly over the last 20 years, these advances have outpaced our system’s capacity to effectively support these youth as they transition to adulthood and adult healthcare. Whether measured as access to quality healthcare, educational, vocational or quality of life outcomes, too many young adults with SLE are faring poorly. But it need not be so.

The ultimate goal of this study was to examine factors explaining self-management skills and transition readiness for youth with jSLE in the context of the dynamic interaction of personal youth, family and healthcare system factors. For the purposes of this abstract, we report only preliminary results of surveys and linked EMR data of 27 patients with jSLE. We anticipate being able to present additional results from our sample which includes youth from four different institutions (~130 youth).

**Objective**

To examine relations between psychosocial variables and health self-management among youth with lupus.

**Methods**

Participants include 27 youth with jSLE from one pediatric rheumatology clinic. Participants completed several surveys and using RedCap. De-identified data from the electronic medical record (EMR) was linked with participants’ responses on the survey. Survey measures include the TRAQ with revised response categories in accord with social loafing theory. Bivariate correlation analysis (Pearsons r) were conducted. Once our sample has reached an adequate size (n=30) we will conduct regression analyses to examine determinants of health self- management among youth with lupus. This study was approved by the Institutional Review Board of Vanderbilt University and East Tennessee State University (IRB# 161674). All psychometric information for instruments utilized are provided in table 1. At the time of abstract submission, demographic information had not been linked to the de-identified database in RedCap.

**Results**

Our analyses demonstrated all scales to have good to excellent reliability. Significant correlations with moderate to strong effect sizes were observed for the associations between self- rated health and competence, and social loafing. Additionally, significant correlations with moderately strong effect sizes were demonstrated for the associations between social loafing and disease knowledge (StarX) and Task value. Further analyses will be conducted using multiple regression to predict independent health self-management.

**Conclusion**

These preliminary analyses show that for youth with jSLE competence was moderately related with poorer health; youth who reported poor health also reported a lack of competence in managing their health. Youth who reported poorer health also reported a lack of independent health self-management as indicated by the social loafing TRAQ scores. Lastly, youth with jSLE who reported more independent health self- management, as indicated by the social loafing TRAQ, also reported more disease knowledge and task value for managing their health.

**Table 1:** **(abstract P22).** Properties of Study Measures

Measures	
Health	
range	1-4
mean(sd)	2.33(.80)
SLEDAI	
range	0-12
mean(sd)	3.4(3.3)
Social Loafing TRAQ	
mean(sd)	3.3 (.47)
reliability	0.87
Task Value	
mean(sd)	4.4(.49)
reliability	0.81

**Table 2:** **(abstract P22).** Bivariate Correlations of study variables

Bivariate correlations						
	Health	SLEDAI	Competence	SLTRAQ	StarX	Task Value
Health (higher scores indicate poorer health)	—	0.12	-.56**	-.46*	-.32	-.43
SLEDAI	—	—	.15	.57	.09	.04
Competence				.26	.21	.40
SLTRAQ	—	—	—	—	.58*	.50*
StarX	—	—	—	—	—	.34
Task Value	—	—	—	—	—	—

* =p<.05; **=p<.01

**Funding**

The authors gratefully acknowledge funding for this project from CARRA and the Arthritis Foundation.

## P23 Epidermal nerve fiber density in juvenile fibromyalgia

### Alexis Boneparth^1^, Shan Chen^2^, Daniel B. Horton^2^, L. Nandini Moorthy^2^, Heather M. Downs^3^, Anne Louise Oaklander^3^

#### ^1^Columbia University Medical Center, New York, NY, USA; ^2^Rutgers Robert Wood Johnson Medical School, New Brunswick, NJ, USA; ^3^Massachusetts General Hospital, Boston, MA, USA

**Background**

Fibromyalgia (FM) is defined by the presence of idiopathic, chronic, widespread pain. The pathogenesis of FM is poorly understood. Adults with FM are known to have decreased epidermal nerve fiber density (ENFD), compared to healthy controls. Although the correlation between decreased ENFD and FM diagnosis is well described in adults, the meaning of this phenomenon is unknown, and the clinical significance of decreased ENFD in FM remains to be elucidated. The prevalence of decreased ENFD in juvenile fibromyalgia (JFM) is unknown. The aims of this study are to: 1) determine whether patients with JFM and their parents will consent to a skin biopsy for ENFD testing; 2) establish a preliminary estimate for the prevalence of decreased ENFD in patients with JFM.

**Methods**

Patients diagnosed with JFM, age 12-22 years old are eligible to participate. Goal enrollment is 15 subjects for skin biopsy to assess ENFD. Subjects diagnosed with JFM by their pediatric rheumatologist complete cross-sectional surveys, assessing 2010 ACR criteria for FM (modified for use in JFM), pain severity, functional disability, and autonomic symptoms severity. Patients meeting 2010 ACR criteria are eligible for collection of a 3mm punch skin biopsy for analysis of ENFD, with comparison of ENFD to existing age-, gender-, and race-based normative data.

**Results**

The first subject was enrolled December 2016. 16 patients with clinically diagnosed JFM have completed the surveys, and 13 met 2010 ACR criteria for JFM. Nine patients have consented for skin biopsy, with median age of 17 years. Five of nine (55%) patients with skin biopsy have ENFD <5^th^ percentile for age/gender/race. Interim analysis does not suggest a correlation between ENFD and measures of pain severity, functional disability, or autonomic symptom severity.

**Conclusion**

Interim results suggest that decreased ENFD is common in JFM. This study continues to enroll patients with goal enrollment of 15 subjects for skin biopsy. Further studies will be needed to assess the clinical significance of decreased ENFD in JFM.

**Ethics Approval**

This study has been approved by the Columbia University Medical Center IRB, protocol number AAAR3311.

**Funding**

This study is supported by the CARRA-Arthritis Foundation Grant Program. The authors wish to acknowledge the ongoing Arthritis Foundation financial support of CARRA.

## P24 Development of a systematic approach to identify factors influencing enrollment in pediatric rheumatology research

### Y. Ingrid Goh^1,2^, Vincent Del Gaizo^3^, Alexandra Sirois^4^, Jennifer E. Weiss^5^, Mary Ellen Riordan^5^, Eyal Muscal^6^, Chivon McMullen-Jackson^6^, Christi J. Inman^7^, Suzy Jones^7^, Jennifer Huntington^7^, Brian M. Feldman^1,2^, for the CARRA Investigators

#### ^1^Division of Rheumatology, The Hospital for Sick Children, Toronto, Ontario, Canada; ^2^Child Health Evaluative Sciences, SickKids Research Institute, Toronto, Ontario, Canada; ^3^Patients, Advocates, And Rheumatology Teams Network For Research And Service (PARTNERS), Atlanta, Georgia, USA; ^4^Rheumatology Family Advisory Council, The Hospital for Sick Children, Toronto, Ontario, Canada; ^5^Department of Pediatric Rheumatology, Hackensack Meridian Health, Joseph M. Sanzari Children’s Hospital, Hackensack, New Jersey, USA; ^6^Section of Immunology, Allergy, and Rheumatology, Baylor College of Medicine, Houston, Texas, USA; ^7^Division of Pediatric Rheumatology, University of Utah Hospitals and Clinics, Salt Lake City, Utah, USA

##### **Correspondence:** Y. Ingrid Goh

**Background**

Many variables affect patients’ and caregivers’ decisions to participate in pediatric clinical research studies. This decision is further complicated when families are asked to participate in research only moments after they have received a diagnosis. While some studies have examined factors influencing study participation, to our knowledge there have not been any studies examining the factors which influence patients’ and caregivers’ enrollment into pediatric rheumatic studies.

The objective was to develop a research project that would help research teams understand how to better engage patients with a newly diagnosed rheumatic condition and their caregivers to join pediatric rheumatology research.

**Methods**

We reviewed papers on the best practices of engaging patients in research. We also reviewed studies which examined factors influencing research participation in other pediatric medical conditions. The knowledge gained through the review was used to select the research team and design the research project.

**Results**

The literature clearly indicated that it was important to have patients and caregivers representation on the research team and have them actively involved in the development of the research project. We reached out to two individuals who immediately joined the study team. After reviewing the characteristics of the Childhood Arthritis and Rheumatology Research Alliance (CARRA) sites, we selected four study sites (The Hospital for Sick Children, Ontario, Canada; Hackensack Meridian Health, New Jersey, USA; University of Utah Hospitals and Clinics, Utah, USA; and Baylor College of Medicine Pediatric Immunology Allergy Rheumatology, Texas, USA.) which represents geographically and demographically diverse populations.

Studies investigating enrollment facilitators and barriers in pediatrics have been conducted in patients receiving oncology and diabetic medications. The methodology used in those studies were adapted to design the following research project.

Patients and caregivers will be recruited to participate in a one-time online focus group prior to their appointment. We will enroll a) new, undiagnosed patients, b) previously diagnosed patients, and c) patients who previously declined to participate in the CARRA registry or other research studies on the day of diagnosis. Individuals consenting to participate will take part in an online, semi-structured focus group session.

Survey questions will be developed from the themes identified during the focus groups to quantify the impact of these themes. This optional one-time anonymous survey will be distributed to all patients and their caregivers attending the rheumatology clinics over a one-month period.

**Conclusion**

Understanding factors which influence enrollment of newly diagnosed patients into studies has been identified as a priority by many CARRA work groups. We hope the results of this research project will help researchers and healthcare team members understand patients' and caregivers' perspectives so they are more mindful in their approach and more sensitive to patients’ and caregivers’ needs.

**Acknowledgements**

This project was recently awarded a CARRA Small Grant.

## P25 Planning grant: crowdsourcing and improving assessment of skin disease in pediatric rheumatology

### Susan Kim^1^, Dexter Hadley^1^, Yvonne Chiu^2^, Mitali Dave^3^, Philip Kahn^4^, Adam Huber^5^, for the CARRA JDM subcommittee

#### ^1^UCSF, San Francisco, CA, USA; ^2^Medical College of Wisconsin, Milwaukee, WI, USA; ^3^Cure JM, Encinitas, CA, USA; ^4^NYU, NY, NY, USA; ^5^Dalhousie University, Halifax, Canada

**Abstract**

Skin and muscle disease is the 'sine qua non' of Juvenile Dermatomyositis (JDM), and is often difficult to diagnose, monitor and treat. Persistent skin disease despite resolution of muscle disease is common, and skin inflammation is associated with worse long term outcomes in JDM.

Many factors limit our ability to monitor skin disease in JDM: we do not have universally accepted approaches to measure and monitor skin disease in JDM. In addition, there is a wide spectrum of skin disease in JDM, whose appearance may vary depending on many factors including ethnicity, complexion and duration of disease. Thus, there is a gap in the measuring and monitoring of skin disease in JDM.

This planning grant was awarded to allow us to develop an approach to “crowd source” patients and clinicians to capture the wide spectrum of skin disease in JDM, with a broad visual representation of skin disease through an *online* platform. We hope that this will allow the future development of an online platform to capture skin disease broadly, to help to better assess and monitor rashes in JDM to advance the quality of care in the future.

**Background**

The Problem: Skin Disease is important in Juvenile Dermatomyositis (JDM) and is often difficult to diagnose, monitor and treat. Persistent skin disease despite resolution of weakness is common in JDM and skin activity predicts worse outcomes. Early skin disease activity in JDM may predict cardiac dysfunction better than muscle disease, and persistently active skin disease months after diagnosis may predict continued active disease [1–4]. Ongoing skin disease reflects ongoing inflammation, which leads to long-term morbidity including lipodystrophy, calcinosis, contractures [3,4]. Furthermore, the rash of dermatomyositis is associated with pain, poor self-image and interferes with normal psychosocial functioning [5]. Thus treatment of JDM-related skin disease is important, since it predicts worse overall outcomes.

Unfortunately, we typically do not measure JDM skin disease objectively in clinical practice, and there are insufficient resources are available to help clinicians and researchers identify and monitor the variety of skin disease in JDM. Without objective regular measurement of disease activity, the degree of active disease, flares and remission are difficult to quantify. Also, currently available reference images of JDM rashes, found in textbooks, online and in manuscripts capture “classic” and severe rashes, typically in fair skinned children, but the range of skin diseases and severity, in a range of skin tones are not available [6].

Proposed solution: With the digitally connected world of today and “crowdsourcing”, we have an opportunity to bridge this gap.  “Crowdsourcing” will allow for rapid collection of images: Collecting photos with conventional approaches, by clinicians alone in the office setting is time consuming and impractical, since rashes may fluctuate with time of day, sun exposure, etc. This “crowdsourcing” approach will allow for timely collection of photos, which is now possible with the broad use of smart phones/cameras in the community. This work will also empower families to participate in the growth of knowledge in JDM.

Once we have a sufficient bank of images, we plan to develop an atlas for JDM will allow improved knowledge and more reliable identification of skin disease in JDM, in teaching, clinical and research settings. In addition, this work will allow us to link the atlas’ photos to currently available skin scoring measures, allowing for broad, effective use outcome measures for skin disease to improve care in JDM.

In summary, we plan to develop an approach to “crowd source” patients and clinicians to capture the wide spectrum of skin disease in JDM, with a broad visual representation of skin disease of skin disease through an on line platform. We hope that this will allow the future development of an online platform to capture skin disease broadly, to help to better assess and monitor rashes in JDM to advance the quality of care in the future.

**Methods**

Aim 1: *To develop an approach to “crowdsource” patients and clinicians to capture a broad visual representation of skin disease in Juvenile Dermatomyositis (JDM)*: This will be achieved with consultation with UCSF CTSI Community Engaged Research and Health Policy Program, Information security office, IT Customer Solutions Management, Legal Office and UCSF IRB regarding this planned project.

Aim 2: *To collaborate with stakeholders in the development of this project, which include clinicians (pediatric rheumatologists and dermatologists) and patients/families*. To form a patient-centered multidisciplinary task force for this project (which will include JDM families, rheumatologists, dermatologists) to develop procedures for obtaining and cataloguing the photos and disseminating the atlas, we plan collaboration with Rheumatologists and Dermatologists from CARRA and PeDRA, in addition to Cure JM parents and families.

**Results**

Aim 1: Meetings to date have helped to identify ethical issues related to broadly collecting photos of patients from families, to ensure that patients/families understand the potential risk of providing photos to identify ethical and logistic barriers related to broad collection of photos of patients from clinicians, while ensuring privacy and consent from patients/families.

We have identified a secure, HIPAA compliant location where this data can be collected and managed: images will be stored on a UCSF approved, HIPAA/HITECH compliant and HITRUST Amazon Web Services (AWS) server. IRB submission for this project was completed in January 2018.

We have a collaborative agreement with Dexter Hadley, MD PhD at UCSF, whose research is with big data, digital health and crowdsourcing to develop an APP to allow us to crowd source images for JDM.

Ongoing/future work: Once IRB is formally approved and APP is updated for our pediatric patients, a pilot phase of image collection is planned. Determining costs for launching this project beyond the pilot-planning phase is to be determined after finalization of the APP.

Aim 2: I have had input from clinician perspective from core group of clinicians within CARRA and PeDRA via phone conferences. Discussions have included: feasibility of this project among patients and clinicians, local/individual IRB concerns, developing easy steps/guidelines for taking photos for clinicians and families. I have engaged Cure JM members regarding this project, and they have agreed to future collaboration with this project, which may include dissemination of this project and research opportunity to Cure JM members.

Ongoing/future work: Face to face meetings at CARRA (April 2018) and PeDRA (October 2018), for introduction/review of this planned project with discussion of project for feedback from general CARRA and PeDRA members, which will include discussion of local/individual IRB concerns, discussion of ideal dissemination of information (E-book, printed book, on-line book, journal publication, etc.), refining steps/guidelines for taking photos for clinicians and families, discussion of procedures cataloguing the photos, future dissemination of data and other research projects that could dovetail with the development of this resource. In addition, we plan for to expand our collaboration with general Cure JM members at national Cure JM meeting (June 2018), for discussion and feedback from the patients/families about this project, including privacy concerns, discussing dissemination of information, research ideas, review of guidelines for taking photos.

**Conclusions**

Skin disease is an important component of disease activity in JDM that needs to be measured and treated. Our planning grant has allowed us to begin to lay the ground work to “crowd source” digital images of the broad and diverse range of rashes in JDM, which will allow for the future improved identification and monitoring of skin disease activity, in hopes of future improvement of overall outcomes in JDM in the future.

**References**

1. Schwartz T, Sanner H, Gjesdal O, Flatø B, Sjaastad I. In juvenile dermatomyositis, cardiac systolic dysfunction is present after long-term follow-up and is predicted by sustained early skin activity. Ann. Rheum. Dis. 2014;73:1805–10.

2. Stringer E, Singh-Grewal D, Feldman BM. Predicting the course of juvenile dermatomyositis: significance of early clinical and laboratory features. Arthritis Rheum. 2008;58:3585–92.

3. Mathiesen P, Hegaard H, Herlin T, Zak M, Pedersen F, Nielsen S. Long-term outcome in patients with juvenile dermatomyositis: a cross-sectional follow-up study. Scand. J. Rheumatol. 2012;41:50–8.

4. Christen-Zaech S, Seshadri R, Sundberg J, Paller AS, Pachman LM. Persistent association of nailfold capillaroscopy changes and skin involvement over thirty-six months with duration of untreated disease in patients with juvenile dermatomyositis. Arthritis Rheum. 2008;58:571–6.

5. Hundley JL, Carroll CL, Lang W, Snively B, Yosipovitch G, Feldman SR, et al. Cutaneous symptoms of dermatomyositis significantly impact patients’ quality of life. J. Am. Acad. Dermatol. 2006;54:217–20.

6. Dugan EM, Huber AM, Miller FW, Rider LG. Photoessay of the cutaneous manifestations of the idiopathic inflammatory myopathies. Dermatol. Online J. 2009;15:1.

## P26 Research priorities among parents and families of children with rheumatic disease

### Colleen K. Correll^1^, Mitali Dave^2^, Anne Paul^3^, Vincent Del Gaizo^4^, Esi M. Morgan^5^, on behalf of CARRA and PARTNERS

#### ^1^University of Minnesota Masonic Children’s Hospital, Minneapolis, MN, USA; ^2^Cure JM Foundation, Encinitas, CA, USA; ^3^Anderson Center for Health Systems Excellence, Cincinnati Children's Hospital Medical Center, Cincinnati, OH, USA; ^4^Parent Partner, Whitehouse Station, NJ, USA; ^5^Cincinnati Children's Hospital, Cincinnati, OH, USA

**Background**

The Patient-Centered Outcomes Research Institute (PCORI) aims to improve the quality and relevance of research by conducting research that is most important to patients and to engage patients at all levels of research. Through the PCORI-supported Patient Powered Research Network, PARTNERS (Patients, Advocates and Rheumatology Teams Network for Research and Service), we surveyed patients and families of children with juvenile myositis (JM), juvenile idiopathic arthritis (JIA), and childhood-onset systemic lupus erythematosus (cSLE) to identify what research topics are most important to them.

**Methods**

This research prioritization exercise was conducted through web-based surveys and focus groups. In Step 1, a link to a survey, which included open-ended questions to assess what concerns patients/families found most important, was emailed to members of Cure JM Foundation, Arthritis Foundation (AF), and Lupus Foundation of America (LFA) listservs and posted on their social media sites. Parents, patients ≥13 years old and family/friends were included. In Step 2, common themes were identified from the open-ended surveys. These themes were characterized further through focus groups for Cure JM and AF. In Step 3, final surveys were created based upon these themes and emailed to the respective listservs and posted to social media sites. Survey respondents were asked to rank the themes most important to them. Responses were weighted and the 7 most important themes were identified. This study was approved by Duke University institutional review board.

**Results**

In Step 1, there were 138 (77% parents), 57 (93% parents), and 47 (55% parents) respondents to the open-ended survey for Cure JM, AF, and LFA, respectively. Response rate could not be calculated because the number of potential respondents from social media sites is unknown. Open-ended responses were qualitatively reviewed and 23, 28, and 16 research themes were identified for Cure JM, AF, and LFA, respectively. In Step 2, these themes were further characterized during two small focus groups affiliated with Cure JM and AF and consisting of parents of children with JM and JIA. In Step 3, the final ranking survey asked respondents to rank the 7 most important priorities from these themes. There were 365, 44, and 32 respondents to this survey for Cure JM, AF, and LFA, respectively. The three research themes common across all disease groups was 1) medication side effects, 2) disease flare and 3) genetic/non-genetic etiology (Table 1). Results from the Cure JM data were presented at two international conferences to help frame the JM research agenda.

**Conclusions**

Patient centered research prioritization is increasingly recognized as a valuable tool in conducting high-quality research, yet there is a lack of publication describing patient/family preferences, especially in pediatrics. Here, we demonstrate a successful program from which we assessed patients/family research priorities in order to frame the agenda for the pediatric rheumatology research community.

**Table 1:** **(abstract P26).** Results from final ranking survey. Top 7 priorities from each group, ranked from highest to lowest priorities

AF	Cure JM*	LFA*
Genetic/Environmental Etiology	New Treatments	Genetic/Environmental Etiology
Personalized medicine	Flares (triggers, prevention, treatment)	Quality of life
Medication side effects	Medication side effects	Medication side effects
Growth and development	Standards to measure disease activity and/or remission	Pain management
Flares (triggers, prevention, treatment)	Genetic/Environmental Etiology	Long-term health/prognosis
New Treatments	JM complications ( i.e. rash, calcinosis, lipodystrophy)	Fatigue
Pain management	Risk of other autoimmune diseases	Flares (triggers, prevention, treatment)

*For Cure JM and LFA, cure was the highest ranking priority. Our aim for this study was to identify research priorities other than cure and thus we eliminated these from the final results

## P27 Higher Serum Complement C4 Levels and *C4B* Gene Copy Numbers Are Associated with Increased Risk for Hypertension and Significant Response to Prophylactic Atorvastatin for Atherosclerosis Prevention in Pediatric Systemic Lupus Erythematosus (pSLE)

### Evan M. Mulvihill^1^, Stacy P. Ardoin^1^, Susan D. Thompson^2^, Bi Zhou^1^, Gakit R. Yu^1^, Nora G. Singer^3^, Deborah M. Levy^4^, Hermine I. Brunner^2^, Yee Ling Wu^1^, Haikady N. Nagaraja^5^, Laura E. Schanberg^6^, Chack-Yung Yu^1^, for the APPLE Investigators

#### ^1^Nationwide Children’s Hospital, Columbus, OH, USA; ^2^Cincinnati Children’s Hospital Medical Center, Cincinnati, OH, USA; ^3^Case Western Reserve University, Cleveland, OH, USA; ^4^The Hospital for Sick Children, Toronto, Canada; ^5^The Ohio State University, Columbus, OH, USA; ^6^Duke University, Durham, NC, USA

##### **Correspondence:** Evan M. Mulvihill

**Objectives**

Patients with pSLE are at risk for poor cardiovascular outcomes. In 2009, the Atherosclerosis Prevention in Pediatric Lupus Erythematosus (APPLE) Trial demonstrated that prophylactic atorvastatin treatment did not significantly reduce progression of carotid intima media thickness (CIMT) in patients with pSLE. A secondary analysis, however, identified a subgroup of post-pubertal individuals with active inflammation at baseline who did significantly respond to atorvastatin. SLE patients are characterized by fluctuating levels of serum complement C4. Serum C4 levels in SLE patients are responsive to inflammation, consumption and variations of gene copy number (GCN) for total *C4*, *C4A* or *C4B*. We hypothesize that genetic diversity of complement C4 modulates the outcome of prophylactic atorvastatin therapy in atherosclerosis of pSLE.

**Patients and Methods**

Clinical data for the 3-year study of 221 pSLE patients, plus genomic DNA and plasma samples for 199 patients were provided by the APPLE repository. Among them, 48.2% were White, 25.5% were Black and 26.4% were from other racial backgrounds. GCNs for total *C4*, *C4A* and *C4B* were measured by TaqMan-based quantitative realtime PCR and Southern blot analyses using genomic DNA. Matched EDTA-plasma samples were used to elucidate C4A and C4B protein polymorphisms by immunofixation. Baseline complement protein levels, and total *C4*, *C4A* and *C4B* GCNs were analyzed with clinical presentations through Student’s t-test for continuous data, and χ^2^ analyses for categorical data. The effects of total *C4*, *C4A* and *C4B* GCN groups on the response to placebo or atorvastatin treatment and progression of CIMT were examined by logistic regression analyses.

**Results**

The GCNs for total *C4*, *C4A* and *C4B* varied between 2 and 6, 0 and 5, and 0 and 5 respectively. At baseline, C4 protein levels were strongly correlated with GCNs of total *C4* (p=1.8x10^-6^) and *C4B* (p=0.0002) and less so with *C4A* (p=0.022). One-third of the patients (N=71) had a past history of hypertension. Compared with normotensive patients (N=142), hypertensive subjects had significantly higher levels of serum C4 (18.5±8.6 mg/dL vs 13.5±6.8 mg/dL; p=4.7x10^-6^), lower mean GCN of *C4A* (1.82±.68 vs 2.07±.76, p=0.032) but higher mean GCN of *C4B* (2.00±.57 vs 1.78±.67, p=0.023). Individuals with at least two copies of *C4B* genes had 2.53 times the odds of having a history of hypertension (p=0.016), and higher diastolic blood pressure (p=0.015) compared to those with a *C4B* deficiency. Baseline CIMT measurements were similar between those two groups (*C4B*≥2 and *C4B*≤1). At the endpoint of the APPLE study, patients with at least two *C4B* copies who were in the atorvastatin treatment group demonstrated significantly less progression of mean-mean CIMT compared to those with the placebo group (p= 0.018).

**Conclusion**

pSLE patients with hypertension had significantly higher C4 protein levels and higher GCN of *C4B*. pSLE patients with ≥2 copies of *C4B* genes receiving atorvastatin treatment demonstrated significantly less progression of mean-mean CIMT than those treated with placebo. Prophylactic atorvastatin for atherosclerosis prevention was more effective for pSLE patients without *C4B* deficiency.

**Ethics Approval**

This study was performed under approved IRB Protocol (IRB14-00544).

## P28 Baseline characteristics and medication use in children with JIA: results from the Childhood Arthritis & Rheumatology Research Alliance Registry

### Sarah Ringold^1^, Anne C. Dennos^2^, Yukiko Kimura^3^, Laura E. Schanberg^4^, Timothy Beukelman^5^, and the CARRA Registry Investigators

#### ^1^Seattle Children's Hospital, Seattle, WA, USA; ^2^Duke Clinical Research Institute, Durham, NC, USA; ^3^Joseph M Sanzari Children's Hospital at Hackensack University Medical Center, Hackensack, NJ, USA; ^4^Duke University Medical Center, Durham, NC, USA; ^5^University of Alabama, Birmingham, AL, USA

**Background**

The Childhood Arthritis and Rheumatology Research Alliance (CARRA) Registry is a multicenter, prospective observational study collecting data from children with rheumatic diseases in order to characterize disease patterns, treatments, and outcomes. The current version of the Registry began enrolling children with JIA in July, 2015. This abstract describes baseline characteristics of children with JIA enrolled as of January, 2018.

Methods

Children were enrolled into the CARRA Registry by participating centers in the US and Canada. Children with all categories of JIA are eligible for enrollment at any time in their disease course. Both retrospective and prospective medication data are collected for each patient.

**Results**

3725 children were enrolled from 59 different centers. The median age at enrollment was 12 years (IQR 8-16 years). 71% were female, 73% had private health insurance, and 81% reported white race. Patients were enrolled at a median of 32 months after their diagnosis (IQR 6 – 74 months), with the majority (78%) enrolled > 12 months after symptom onset. Patients from each ILAR category have been enrolled. The most common categories were RF negative polyarthritis (35%) and persistent oligoarticular arthritis (27%). 73% of patients had used methotrexate at the time of enrollment or prior to enrollment. 65% had used a tumor necrosis factor α inhibitor at the time of enrollment or prior to enrollment. Biologics with other mechanisms of action were used less commonly: 9% interleukin-1 inhibitors, 7% tocilizumab, and 4% abatacept.

**Conclusion**

Biologic use was common among this large cohort of children with JIA. Longitudinal data generated from the long-term follow-up of Registry participants will provide additional important data on patterns of medication use, medication safety, and disease outcomes.

**Acknowledgements**

The authors wish to acknowledge CARRA, and the ongoing Arthritis Foundation financial support of CARRA.

## P29 The Childhood Arthritis and Rheumatology Research Alliance start time optimization of biologic therapy in polyarticular JIA study: interim report of baseline patient characteristics and treatment choices

### Sarah Ringold^1^, George Tomlinson^2^, Pamela F. Weiss^3^, Laura E. Schanberg^4^, Brian M. Feldman^5^, Mary Ellen Riordan^6^, Anne C. Dennos^7^, Vincent Del Gaizo^8^, Katherine L. Murphy^8^, Yukiko Kimura^*6^, and the CARRA Registry Investigators

#### ^1^Seattle Children's Hospital, Seattle, WA, USA; ^2^University of Toronto, Toronto, Canada; ^3^Children's Hospital of Philadelphia, Philadelphia, PA, USA; ^4^Duke University Medical Center, Durham, NC, USA; ^5^The Hospital for Sick Children, Toronto, Canada; ^6^Joseph M Sanzari Children's Hospital at Hackensack University Medical Center, Hackensack, NJ, USA; ^7^Duke Clinical Research Institute, Durham, NC, USA; ^8^CARRA Parent/Patient Partner, Whitehorse Station, NJ, USA

##### **Correspondence:** Sarah Ringold

**Background**

Despite the many available new and effective treatments for polyarticular JIA (P-JIA), there is significant variation in the timing of when biologic medications are started. Three consensus treatment plans (CTPs) reflecting the currently most commonly-used strategies for starting biologic treatment were developed by the Childhood Arthritis and Rheumatology Research Alliance (CARRA) using consensus methodology. The CARRA Start Time Optimization of Biologic Therapy in Polyarticular JIA (STOP-JIA) study aims to compare the effectiveness of the three CARRA P-JIA CTPs using a prospective, observational study design. This abstract describes interim baseline characteristics and CTP choices for the patients enrolled in STOP-JIA.

**Methods**

Untreated P-JIA patients were enrolled into the CARRA Registry. Providers and patients together chose one of the CTPs to follow: 1) Step-Up treatment (initial therapy with DMARD and biologic added after 3 months if needed); 2) Early Combination (initial therapy with both DMARD and biologic); and 3) Biologic First (initial treatment with biologic monotherapy). Providers had the option of prescribing glucocorticoids at baseline per their usual practice and were provided with tapering options. There is no randomization or blinding in this observational study.

**Results**

Three hundred and six patients were enrolled at 42 sites in the US and Canada between 1 Nov 15 and 11 Jan 18. Patient characteristics of participants with data entered (n=273) are summarized in Table 1. The most commonly chosen CTP was Step-Up (n=178; 64%). Early Combination CTP was the next most common choice (n=66; 24%). To date, 590 follow up visits have been entered and 89 patients have completed their 12-month visit. Of the patients who have completed their 3-month visit, 10 patients were reported to have changed CTP. There were 22 Serious Adverse Events (SAE) or Events of Special Interest (ESI) including 4 cases of new onset uveitis, 7 infections, and 4 episodes of hepatitis.

**Conclusion**

To date, patients have been enrolled into all 3 CTP choices, with the Step-Up CTP being the most common. Ongoing, prospective data collection from these patients will allow for a comparison of the effectiveness of the strategies.

**Table 1:** **(abstract P29).** Baseline Patient Characteristics

	Total Cohort (n= 273)	Step up (n= 178)	Early Combination (n=66)	Biologic First (n=29)
Female N (%)	196 (72)	132 (74)	44 (67)	20 (69)
White N (%)	192 (70)	130 (73)	44 (67)	18 (62)
Age in yrs – mean (range)	10 (1-18)	10 (1-18)	11 (1-17)	11 (1-18)
JIA Category N (%)				
Extended Oligoarticular	6 (2)	6 (3)	--	--
Polyarticular (RF-)	174 (64)	127 (71)	35 (53)	12 (41)
Polyarticular (RF+)	48 (18)	27 (15)	17 (26)	4 (14)
Psoriatic	14 (5)	6 (3)	4 (6)	4 (14)
Enthesitis-related	24 (9)	10 (6)	8 (12)	6 (21)
Undifferentiated	6 (2)	2 (1)	2 (3)	2 (7)
Number of Active joints - mean (range)	13 (5-50)	12 (5-49)	17 (5-50)	11 (5-41)
Physician Global Assessment of Disease Activity - mean (range)	6 (0-10)	5 (0-10)	6 (1-10)	6 (1-10)
Juvenile Arthritis Disease Activity Score - mean (range)	18 (5-29)	17 (5-29)	20 (6-29)	20 (14-28)
CHAQ Score - mean (range)	1 (0-3)	1 (0-3)	1 (0-3)	1 (0-3)
Oral steroids prescribed at baseline - N (%)	75 (27)	50 (28)	22 (33)	3 (10)

**Trial registration identifying number**

NCT02593006

**Ethics Approval**

Pro00054616 (Duke University)

**Acknowledgements**

The authors wish to acknowledge CARRA, and the ongoing Arthritis Foundation financial support of CARRA.

## P30 Factors related to sustained discontinuation of medications for well-controlled JIA in the Childhood Arthritis & Rheumatology Research Alliance Registry

### Daniel B. Horton^1,2^, Fenglong Xie^3^, Melissa L. Mannion^3^, Sarah Ringold^4^, Colleen K. Correll^5^, Anne Dennos^6^, Timothy Beukelman^3^, for the CARRA Registry Investigators

#### ^1^Rutgers Robert Wood Johnson Medical School, New Brunswick, NJ, USA; ^2^Rutgers Institute for Health, Health Care Policy and Aging Research, New Brunswick, NJ, USA; ^3^University of Alabama at Birmingham, Birmingham, AL, USA; ^4^Seattle Children's Hospital, Seattle, WA, USA; ^5^University of Minnesota, Minneapolis, MN, USA; ^6^Duke Clinical Research Institute, Durham, NC, USA

**Background**

Stopping medications is a priority for many patients with well-controlled JIA, but few factors predict favorable outcomes after discontinuation. We examined factors associated with sustained discontinuation off disease-modifying drugs in a large pediatric registry.

**Methods**

We conducted a case-control study using the Childhood Arthritis & Rheumatology Research Alliance Registry of clinical data from >55 pediatric rheumatology clinics in the United States and Canada. The study included children with JIA who started at least 1 conventional or biologic DMARD and had at least 2 years of available subsequent data. Reasons for drug discontinuation were obtained from the medication log. Cases with sustained discontinuation had at least 1 year of drug-free follow-up after discontinuation for well-controlled disease. Comparators included children who stopped DMARDs for at least 30 days and restarted within 1 year (control group 1) and those who did not discontinue all DMARDs for well-controlled disease for ≥30 days (control group 2). Children in each group may have stopped DMARDs for reasons besides well-controlled JIA. We excluded children with <1 year of follow-up off medicines without restarting DMARDs. Characteristics of cases and controls were compared using descriptive statistics. P-values were calculated by Wilcoxon rank sum testing for continuous variables and by chi-square or Fisher's exact test for categorical variables.

**Results**

There were 1338 children with JIA in the study who started DMARDs, of whom 545 (41%) stopped all drugs due to well-controlled disease after median 1.9 years of DMARD use (interquartile range [IQR] 1.1, 3.2). Of these, 222 (41%) had sustained discontinuation for ≥1 year (cases) and 323 (59%) restarted DMARDs within 1 year (control group 1) (Table 1). In control group 1 with unsustained discontinuation, only 24 children (10% of control group 1, or 2% of the total cohort) subsequently stopped all DMARDs for ≥1 year. Among all children in the study who started a DMARD, cases with sustained discontinuation were younger at diagnosis (P < 0.01), more likely to have systemic JIA and less likely to have RF+ polyarticular JIA (P = 0.03), less likely to have complications from uveitis (P < 0.01), and less likely to have used a biologic DMARD (<0.01). Compared with controls with unsustained discontinuation, cases were less likely to have complications from uveitis (P = 0.05) or have used biologics (<0.01). Time to diagnosis, history of uveitis, radiographic joint damage, and conventional DMARD use were not associated with sustained discontinuation in either comparison.

**Conclusion**

In a large multicenter cohort of children with JIA on DMARDs, only 1 in 5 children stopped DMARDs for well-controlled disease for at least 1 year. Lack of uveitis complications and exclusive use of conventional DMARDs were associated with sustained discontinuation.

**Table 1:** **(abstract P30).** Characteristics of study subjects with JIA who started at least 1 DMARD

Characteristic (N, % unless otherwise noted)	Cases: off DMARDs ≥12 months^1^ (N=222)	Control group 1: off DMARDs <12 months^1^ (N=323)	Control group 2: not off DMARDs >30 days (N=793)	P-value^2^ (cases vs. control groups 1 and 2)	P-value^2^ (cases vs. control group 1)
Demographic					
Age at diagnosis (years), median (IQR)	4.0 (2.0, 8.0)	4.0 (2.0, 8.0)	6.0 (2.0, 11.0)	<0.01	0.70
Time from symptom onset to diagnosis (days), median (IQR)	47 (29, 86)	58 (26, 92)	54 (29, 132)	0.49	0.61
Time from diagnosis to start of first DMARD (days), median (IQR)	37 (1, 201)	28 (1, 230)	34 (1, 350)	0.59	0.93
Time from start of DMARD to first DMARD discontinuation (years), median (IQR)	1.9 (1.2, 3.3)	1.9 (1.1, 3.1)	-	-	0.51
Time from start of first DMARD to end of follow-up (years), median (IQR)	6.0 (4.4, 8.8)	5.9 (3.9, 8.6)	4.2 (2.8, 6.7)	<0.01	0.26
Female sex	160 (72%)	253 (78%)	605 (76%)	0.13	0.09
White race	187 (84%)	272 (84%)	646 (82%)	0.47	0.99
Latino ethnicity	22 (13%)	22 (8%)	83 (12%)	0.83	0.46
Residence in US	217 (98%)	314 (97%)	770 (97%)	0.61	0.70
Clinical					
JIA category				0.03	0.14
Systemic	33 (15%)	30 (12%)	107 (14%)		
RF+ polyarticular	11 (5%)	19 (6%)	98 (12%)		
Other	178 (80%)	272 (85%)	588 (74%)		
≥5 total joints affected	193 (87%)	295 (91%)	719 (91%)	0.15	0.23
Radiographic evidence of joint damage	31 (14%)	56 (17%)	117 (15%)	0.55	0.28
ANA positive	96 (43%)	139 (43%)	334 (42%)	0.81	0.96
History of uveitis	19 (9%)	19 (6%)	80 (10%)	0.86	0.23
Uveitis complications	0	7 (2%)	22 (3%)	<0.01	0.05
Any conventional DMARD use	201 (91%)	287 (89%)	719 (91%)	0.86	0.53
Any biologic DMARD use	84 (38%)	174 (54%)	691 (87%)	<0.01	<0.01

*ANA* antinuclear antibody, *DMARD* disease-modifying antirheumatic drug, *IQR* interquartile range, *RF* rheumatoid factor

^1^Refers to discontinuation from all DMARDs for at least 30 days for well-controlled JIA

^2^P-values calculated by Wilcoxon rank sum testing for continuous variables and chi-square or Fisher's exact test for categorical variables

**Ethics Approval**

The study was approved by University of Alabama's Institutional Review Board, protocol number X170112004.

**Funding**

Funding for this work came from a CARRA-AF Publication Support Grant.

## P31 Characteristics of patients enrolled in the FiRst line options for systemic jia treatment (FROST) Consensus Treatment Plan study

### Timothy Beukelman^1^, Peter A Nigrovic^2^, George Tomlinson^3^, Vincent Del Gaizo, Marian Jelinek, Laura E. Schanberg^4^, Anne Dennos^4^, Mary Ellen Riordan^5^, Yukiko Kimura^5^, for the CARRA Registry FROST Investigators

#### ^1^University of Alabama at Birmingham, Birmingham, AL, USA; ^2^Brigham and Women’s Hospital, Harvard University, Boston, MA, USA; ^3^University of Toronto, Toronto, ON, Canada; ^4^Duke University, Durham, NC, USA; ^5^Hackensack University Medical Center, Hackensack, NJ, USA

**Background**

The optimal initial treatment for systemic juvenile idiopathic arthritis (sJIA) is unclear. Uncontrolled reports suggest that early treatment with biologic agents likely produces superior short-term clinical outcomes, but many patients may respond well to non-biologic therapies. To further study the initial treatment of sJIA, the Childhood Arthritis and Rheumatology Research Alliance (CARRA) developed Consensus Treatment Plans (CTPs) to formalize and standardize current treatment practices. Subsequently, 4 CTPs were developed: initial systemic glucocorticoid (GC); initial methotrexate (MTX) +/- GC; initial IL-1 inhibition (IL-1i) +/- GC; and initial IL-6 inhibition (IL-6i) +/- GC. The FiRst Line Options for Systemic JIA Treatment (FROST) is an observational study designed to assess the effectiveness and safety of each of the 4 CTPs and to the compare the CTPs containing initial biologic therapy (IL-1i and IL-6i) to those that do not contain initial biologic therapy (GC and MTX).

**Methods**

Patients with recent onset sJIA who are initiating therapy are considered for enrollment in FROST. In order to be eligible for FROST, participants must have fever for ≥2 weeks, arthritis for ≥ 10 days, and at least 1 of the following: evanescent rash, generalized lymphadenopathy, hepatomegaly, splenomegaly, or serositis. Treatment assignment is at the discretion of the treating physician and family. All data are collected in the CARRA Registry. To date, 43 CARRA Registry sites have been activated to enroll FROST patients. Biosamples are collected at baseline and at 6 months. Patient reported outcomes (PRO) of presence of fever and rash and pain scores are collected at home using mobile devices every 2 days during the first 2 weeks of the study. The primary study outcome is clinical inactive disease (Wallace ACR provisional definition) and cessation of glucocorticoid therapy at 9 months.

**Results**

Enrollment in the FROST study began in November 2016. As of January 2018, 21 patients have been enrolled at 13 sites, and their baseline characteristics are shown in the Table 1. Overall, 11 participants (52%) have completed every requested home PRO during the first 2 weeks of the study, and 15 (71%) have completed ≥50% of the requested home PRO. Study follow-up continues to accrue. Five patients (25%) have completed the 9-month study visit when the primary study outcome is assessed. Nine patients (43%) overall have completed the 6-month visit. Of the 20 participants, 10 (50%) had baseline biosamples collected.

**Conclusion**

Enrollment in the FROST study has begun. Participants enrolled thus far appear to be representative of the general population of patients with sJIA. Home PRO collection is feasible through the CARRA Registry. Additional time is needed to enroll and observe a sufficient number of patients to assess the comparative effectiveness of initial treatments for sJIA.

**Table 1:** **(abstract P31).** Characteristics of Participants in FROST (N=20)

Characteristic	Overall (N=20)
Age (mean (sd)	7.85 (5.33)
Female (%)	10 (50%)
White Race (%)	10 (52.6%)
Black Race (%)	3 (15.8%)
Other Race (%)	6 (31.6%)
Elapsed Days Since Onset of Symptoms (mean (sd))	47 (58)
Elapsed Days Since Diagnosis (mean (sd))	7 (7)
Physician Global Assessment (mean (sd))	6.00 (2.22)
Parent Global Assessment (mean (sd))	4.53 (3.43)
Number of Active Joints (mean (sd))	5.80 (5.59)
Morning Stiffness >15 minutes (60%)	12 (60%)
ESR (median (IQR))	58 (42-83)
C-RP (median (IQR))	10.6 (6.6-32.1)
Ferritin (median (IQR))	891 (284-1510)
Hemoglobin (median (IQR))	10.2 (9.5-11.3)
WBC (median (IQR))	14.9 (10.5-20.3)
Platelets (median (IQR))	368 (294-510)
AST (median (IQR))	34 (23-45)
ALT (median (IQR))	34 (16-51)
CHAQ (mean (sd))	1.00 (0.86)
Pain VAS (mean (sd))	3.5 (3.332)

## P32 Systemic juvenile idiopathic arthritis inactive disease and withdrawal of medications: a survey of the Childhood Arthritis and Rheumatology Research Alliance

### Kabita Nanda^1^, Susan Shenoi^1^, Ashley Cooper_2_, Bridget Edghill^3^, Miriah Gillispie^4^, Baruch Goldberg^5^, Olha Halyabar^6^, Daniel B. Horton^7^, Thomas G. Mason^8^, Tova Ronis^9^, Rayfel Schneider^10^, Grant Schulert^11^, Richard Vehe^12^, Karen Onel^13^, for the SJIA workgroup of the CARRA JIA subcommittee

#### ^1^Seattle Children’s Hospital, University of Washington School of Medicine, Seattle, WA, USA; ^2^Children’s Mercy-Kansas City, University of Missouri-Kansas City, Kansas City, MO, USA; ^3^Comminuty stakeholder CARRA member; ^4^Texas Children’s Hospital, Baylor College of Medicine, Houston, TX, USA; ^5^McGovern Medical School at UT Health, Houston, TX, USA; ^6^Boston Children’s Hospital, Harvard Medical School, Boston, MA, USA; ^7^Rutgers Robert Wood Johnson Medical School, New Brunswick, NJ, USA; ^8^Mayo Clinic, Rochester, MN, USA; ^9^Children’s National Health System, The George Washington School of Medicine, Washington, DC, USA; ^10^The Hospital for Sick Children, University of Toronto, Toronto, ON, Canada; ^11^Cincinnati Children’s Hospital, University of Cincinnati College of Medicine, Cincinnati, OH, USA; ^12^Masonic Children’s Hospital, University of Minnesota, Minneapolis, MN, USA ^13^ Hospital for Special Surgery, Weill Cornell Medical College, New York, NY, USA

##### **Correspondence:** Kabita Nanda

**Background**

There has been rapid development in new treatments for systemic juvenile idiopathic arthritis (SJIA) but there is no consensus on when and how to withdraw medications. There are validated criteria for clinical inactive disease (CID) in juvenile idiopathic arthritis (JIA); however, as SJIA is considered an autoinflammatory disease there may be additional criteria to consider. The SJIA CARRA workgroup sought to obtain opinions on CID in SJIA and how physicians approach withdrawal of medications.

**Methods**

An anonymous electronic survey using REDCap was sent to 100 randomly selected voting members of CARRA. The survey elicited physicians’ opinions on CID in SJIA and physicians’ current approaches to withdrawal of medications in CID. Descriptive statistics were used to analyze the data.

**Results**

Eighty-three of the 100 surveyed CARRA members completed the survey including 7 members that opted out, as they are not involved in clinical care of SJIA. The vast majority of participants (88%) agreed with the current criteria for CID in SJIA. Stated reasons for dissent included lack of ferritin and other inflammatory markers in the criteria, presence of uveitis in the criteria, preference for extending duration of morning stiffness, preference for changing duration of time required for CID, imprecision of physician global score and lack of patient/parent-reported outcomes. Ninety-three percent agreed with the current definition for clinical remission on medications (CRM) in SJIA. Disagreement was due to preference for 1 year (not 6 months) of inactive disease to meet CRM. Most felt it was necessary to meet CRM (78%) before tapering medications other than steroids, but others stated preferences for withdrawing other therapy before 6 months of CID. Most members (76%) reported using the CARRA SJIA consensus treatment plans always or the majority of the time. All members reported that they weaned steroids first in SJIA patients on combination therapy, 47% reported waiting greater than 6 months before tapering additional medications, and 37% preferred waiting only 2-6 months. An equal number of members (35% each) reported tapering methotrexate over more than 6 months and 2-6 months; however a higher proportion (39%) preferred tapering anakinra, canakinumab and tocilizumab more quickly over 2-6 months and favor spacing the dosing interval for canakinumab and tocilizumab. When patients are on combination therapy with methotrexate and biologics, 58% preferred tapering methotrexate first while most others would consider patient/family preference and adverse effects to guide their choice.

**Conclusion**

Most CARRA members surveyed are using previously published consensus treatment plans for SJIA and agreed with validated definitions of CID and CRM. There was also agreement with tapering steroids first in SJIA, but there was considerable variability with all other medications. Further work will need to be done to develop consensus in withdrawal plans for medications in SJIA.

**Ethics Approval**

The study was approved by Seattle Children’s Hospital’s Internal Review Board, ID: STUDY00000532, and Hospital for Special Surgery’s Internal Review Board, ID: 2017-0276.

## P33 The lupus cohort in the New CARRA Registry: 10 months of enrollment

### Mary Beth Son^1^, Stacy P. Ardoin^2^, Aimee O. Hersh^3^, Deborah Levy^4^ for the CARRA Registry Investigators

#### ^1^Boston Children’s Hospital, Boston, MA, USA; ^2^Nationwide Children’s Hospital, Columbus, OH, USA; ^3^University of Utah, Salt Lake City, UT, USA; ^4^Hospital for Sick Children, Toronto, Canada

##### **Correspondence:** Mary Beth Son

**Background**

The New CARRA Registry has enrolled patients with systemic lupus erythematosus (SLE) and related conditions since March 2017. We sought to describe the population enrolled thus far and to demonstrate the breadth and utility of the data generated by the New CARRA Registry.

**Methods**

We requested de-identified counts of several fields collected from the case report forms for subjects with SLE. Patients were eligible for enrollment in the New CARRA registry if they were diagnosed with SLE or had a flare of lupus nephritis within two years of the baseline visit. IRB approval was not required for this data request.

**Results**

To date, 102 patients (pts) have been enrolled; 88 (86%) are female. There are 30 black pts, 28 Hispanic pts, 27 white pts, 7 Asian pts and 6 pts were >1 race. Over half the pts have private health insurance (n=57, 56%) and 35 pts (34%) have Medicaid. 96 pts were ANA positive. The distribution of autoantibodies documented at the baseline visit is shown in Table 1. At the baseline visit, the mean Systemic Lupus Erythematosus Disease Activity Index (SLEDAI, n=98) score was 6.6 ± 6.5, median =6 (range 0-37). The mean Systemic Lupus International Collaborating Clinic Damage Index (SLICC DI, n=86) score was 0.4, median=0 (range 0-4). Nearly 85% of pts were prescribed corticosteroids at the baseline visit. A quarter of pts (n=26) were being treated for lupus nephritis at the time of the baseline visit. Manifestations of SLE at the baseline visit were varied (Table 2) but serologic disease, mucocutaneous disease and active nephritis were the most prevalent.

**Conclusions**

Over a hundred SLE pts have been enrolled in the New CARRA Registry to date. This is a multi-racial cohort with moderate disease activity and varied disease manifestations. Further enrollment will continue to build a robust data source to study disease course and outcomes in an incipient pediatric SLE cohort.

**Table 1:** **(abstract P33).** Auto-Antibody Data Documented at Baseline Visit (n=102)

Positive Auto-Antibodies	N (%)
ANA	96 (94%)
Anti-Smith	50 (49%)
Anti-dsDNA	61 (60%)
Anti-RNP	53 (52%)
Anti SSA	32 (31%)
Anti SSB	17 (16.7%)
Anti β2 glycoprotein I IgG	6 (5.9%)
Anti Cardiolipin IgG	18 (17.7%)
Lupus anti-coagulant	15 (14.7%)

**Table 2:** **(abstract P33).** Components of SLEDAI Present Within 30 days of Baseline Visit (n=98)

SLEDAI Component	N (%)
Seizure	1 (1%)
Psychosis	1 (1%)
Organic Brain Syndrome	0
Visual Disturbance	1 (1%)
Cranial Nerve Disorder	0
Lupus Headache	4 (3.9%)
Cerebrovascular Accident	0
Vasculitis	2 (2%)
Arthritis	16 (15.7%)
Myositis	5 (4.9%)
Urinary Casts	18 (17.6%)
Hematuria	9 (8.8%)
Proteinuria	9 (8.8%)
Pyuria	8 (7.8%)
Rash	21 (20.6%)
Alopecia	7 (6.9%)
Mucosal Ulcers	15 (14.7%)
Pleurisy	0
Pericarditis	0
Low Complement	45 (44.1%)
Increased DNA Binding	34 (33.3%)
Fever	7 (6.9%)
Thrombocytopenia	4 (3.9%)
Leukopenia	13 (12.7%)

## P34 Towards a better understanding of childhood Sjögren syndrome: evaluation of the 2016 ACR/EULAR classification criteria for use in diagnosing Sjögren syndrome in children

### Sara Stern^1^, Jay Mehta^2^, Scott Lieberman^3^ for the International Childhood Sjögren Syndrome Workgroup

#### ^1^Department of Pediatrics, University of Utah Health Sciences Center, Salt Lake City, UT, USA; ^2^Department of Pediatrics, University of Pennsylvania Perelman School of Medicine, Philadelphia, PA, USA; ^3^Stead Family Department of Pediatrics, University of Iowa, Iowa City, IA, USA

**Background**

Clinical presentation of Sjögren syndrome in children differs from that in adults: dryness symptoms are more common in adults, while parotitis is more common in children. Criteria developed for adult classification have demonstrated low sensitivity when applied to pediatric populations, and no child-specific criteria have been established. The latest adult classification criteria have not yet been evaluated for use in children. Our objective was to evaluate the applicability of these new criteria for use in children.

**Methods**

Retrospective chart reviews were conducted to collect individual patient level data for children diagnosed with Sjögren syndrome (based on clinical diagnosis at age <18 years). Data including clinical features, laboratory values, imaging studies, and test items in the 2016 ACR/EULAR criteria were collected, and de-identified data were entered into a REDCap database. This study was approved by the Institutional Review Boards or equivalent regulatory bodies at individual affiliate institutions.

**Results**

To date, 94 children with Sjögren syndrome were included from 12 institutions across 4 countries–data collection is ongoing. This constitutes the largest childhood Sjögren syndrome patient series to date. The majority of children (90%) were female with a mean age of 11.6 years at diagnosis (range 1–17.8 years). Fourteen children (15%) also had another autoimmune disease (9 with SLE, 2 with uveitis, 1 with MCTD, 1 with myositis/overlap, 1 with subacute cutaneous lupus). Frequency of clinical features were as follows: 53% with dry eyes, 52% with dry mouth, 50% with parotitis, 44% with arthralgias without arthritis, 24% with lymphadenopathy, 27% with arthritis, 14% with cytopenias, 15% with fevers, 12% with cutaneous vasculitis, and ≤10% each with weight loss, recurrent vaginitis, myositis, pulmonary, renal, or neurologic manifestations. Only 6 children had testing for all 5 items included in the 2016 ACR/EULAR criteria. Most children (95%) had testing for anti-SSA antibodies, but fewer underwent minor salivary gland (MSG) biopsy (53%), Schirmer testing (53%), unstimulated whole saliva flow (UWSF, 16%), or ocular surface staining (OSS, 20%). While most children studied (94%) were missing at least one data point, 32 of 94 children (34%) met the 2016 ACR/EULAR classification criteria for Sjögren syndrome. Of these 32 children: 29 (91%) had positive anti-SSA antibodies; 21 (66%) had positive MSG biopsy; 25 (78%) had positive Schirmer test; 3 (9%) had positive UWSF; and 1 (3%) had positive OSS. Of the 62 children not meeting criteria: 39 (63%) had positive anti-SSA antibodies; 7 (11%) had positive MSG biopsy; 8 (13%) had positive Schirmer test; and 3 (5%) had positive UWSF.

**Conclusions**

Criteria items from the 2016 ACR/EULAR criteria are not routinely assessed in children diagnosed with Sjögren syndrome making formal retrospective assessment of criteria difficult. Prospective study of these criteria along with defining child-specific normal values and adding child-specific criteria items (such as recurrent parotitis) are warranted. Establishing criteria for childhood Sjögren syndrome is a key step toward better understanding this condition.

## P35 Consenting for Biological specimens collection: the healthcare provider and research coordinator perspective

### Y. Ingrid Goh^1,2^, Lauren A. Henderson^3^, Jennifer M.P. Woo^4^, Marsha Malloy^5^, Mary Ellen Riordan^6^, CARRA Research Coordinator Advisory Committee (RCAC), CARRA Translational Research and Technology Committee (TRTC) Biobanking Workgroup

#### ^1^Division of Rheumatology, The Hospital for Sick Children, Toronto, Ontario, Canada; ^2^Child Health Evaluative Sciences, SickKids Research institute, Toronto, Ontario, Canada; ^3^Division of Immunology, Boston Children’s Hospital, Boston, Massachusetts, USA; ^4^Division of Pediatric Rheumatology, UCLA Mattel Children’s Hospital, Los Angeles, California, USA; ^5^Division of Rheumatology, Medical Center of Wisconsin, Milwaukee, Wisconsin, USA; ^6^Department of Pediatric Rheumatology, Hackensack Meridian Health, Joseph M. Sanzari Children’s Hospital, Hackensack, New Jersey, USA

##### **Correspondence:** Y. Ingrid Goh

**Background**

Biological specimens are important as they enable researchers to elucidate the links between biological pathways and disease manifestation. Specimens can be used to provide insight about why things happen; who is at risk; how it happens; understand what happens in the body; and how to improve diagnosis and treatment. The knowledge garnered through these specimens can ultimately lead to personalized medicine.

Biological specimen collection for Childhood Arthritis and Rheumatology Research Alliance (CARRA) started in 2017. To date, specimens have been collected from over 60 unique pediatric patients with rheumatologic disease residing in North America who were initiating medications.

Recruiting newly diagnosed pediatric patients can be a challenging feat as this is an emotional and psychologically taxing time for patients and their family. The objective of this project was to identify best practices that can be used by CARRA clinicians and research coordinators when consenting patients to donate biological specimens for research.

**Methods**

Members of the CARRA Translational Research and Technology Committee (TRTC) Biobanking Workgroup were asked to identify key points that are commonly discussed during the biological specimen consent process. They were also asked to provide examples of specific language used during the consent process. These items were collectively reviewed and discussed during a series of conference calls with TRTC Biobanking Workgroup and the CARRA Research Coordinator Advisory Committee (RCAC). A single document containing the items was prepared and underwent a final review by the TRTC Biobanking Workgroup and RCAC.

**Results**

Key points were received from CARRA TRTC biobank subgroup and RCAC members representing five unique CARRA sites. After reviewing, discussing, and consolidating, 14 main topics were identified as important items to address during the consent process. In addition to these themes, it was thought to be beneficial, and at some institutions required, to have a member of the healthcare team introduce the study prior to consent in order to establish rapport. It was unanimously agreed that it is important to spend time educating patients and their families about what research is and what it could potentially mean for them and future patients. It was deemed important to use clear and approachable language that patients and their families could understand when explaining the requirements of the proposed research.

**Conclusion**

The key elements identified by the CARRA TRTC Biobanking Workgroup and RCAC have been made available to CARRA members on the CARRA Coordinator internal webpage. The key points will be reviewed with the wider CARRA Research Coordinator Network during the 2018 CARRA meeting. We anticipate that this information will be beneficial to less experienced teams or teams that are experiencing difficulty recruiting patients to participate in biological specimen collection.

## P36 Validation of synovial fluid biomarkers for prediction of extension in oligoarticular juvenile idiopathic arthritis

### AnneMarie C. Brescia^1^, Megan M. Simonds^2^, Suzanne M. McCahan^2^, H. Timothy Bunnell^2^, Kathleen E. Sullivan^3^, Carlos D. Rose^1^

#### ^1^Nemours/AI DuPont Hospital for Children, Wilmington, DE, USA; ^2^Nemours Biomedical Research, Wilmington, DE, USA; ^3^Pediatric Immunology, Children’s Hospital of Philadelphia, PA, USA

##### **Correspondence:** AnneMarie C. Brescia

**Background**

Our goal is validation of synovial biomarkers to predict which children with oligoarticular juvenile idiopathic arthritis (JIA) will have persistent vs. extended course. We previously identified 5 candidate biomarkers from gene expression data of fibroblast-like synoviocytes (FLS) of 12 P and 7 E patients^1^. In the current study, we are expanding the pool of potential biomarkers using antibody array.

**Methods**

As part of an ongoing IRB approved protocol, remnant synovial fluid was obtained from patients undergoing medically indicated arthrocenteses. Using our clinical database, JIA samples were separated into two groups: (1) oligoarticular JIA with persistent course (P), (2) oligoarticular JIA with extended course, prior to extension (E). Using synovial fluid samples from 4 P and 4 E patients, RayBiotech Membrane-based Antibody Arrays were completed for expression of 89 cytokines, chemokines, and other inflammatory proteins and intensity measured using ImageJ. Twenty proteins were differentially expressed between P and E. We chose 6 biologically relevant differentially expressed proteins identified in synovial fluid by antibody arrays (**angiogenin, IL-5, VEGF, IL-1α, IL-6 and IL-3**) to add to our already identified group of candidate biomarkers from our gene expression data (**MBP, ANKRD44, HSPBAP1, KLHL13, and CD14**). To translate these differences into a clinically testable biomarker panel, we tested the corresponding synovial fluids for protein levels by ELISA. We conducted extensive chart review to identify as yet untested samples for biomarker validation testing (exclusions included, but were not limited to: prior injection, medications besides non-steroidal anti-inflammatory medications, already extended at time of sample, co-morbid conditions with associated arthropathy). We tested synovial fluid from 14 P and 8 E, which had been blinded by an honest broker. Using statistical cutoffs for each protein as determined by ELISA, each blinded sample was assigned to a group, P or E.

**Results**

We have completed this validation for 5/11 of the candidate biomarkers so far. The performance of each protein to correctly assign an E sample (sensitivity) and as not likely to extend (remain P, specificity) is shown in **Table 1**. There was reasonable specificity for all the biomarkers, however, CD14 was the most specific. IL-6 was the most sensitive for accurately predicting which synovial fluid came from patients who would ultimately extend.

**Conclusion**

We have been able to demonstrate differential synovial fluid protein expression from JIA patients who remained P vs those who are destined to extend, demonstrating detectible difference early in disease that may be useful for prediction. We are now looking to validate these potential biomarkers on a new larger validation cohort from outside our institution.

**Ethics Approval**

The study was approved by Nemours Internal Review Board, (84709, 82053).

**Table 1:** **(abstract P36).** Sensitivity and Specificity of each biomarker to assign blinded samples to the correct group, extended or persistent

	Sensitivity	Specificity
CD14	29%	86%
IL-1alpha	14%	50%
IL-5	57%	64%
IL-6	71%	71%
IL-3	14%	71%

**Reference**

1 Pediatric Rheumatol 2018 Jan 8;16(1):3. 10.1186/s12969-017-0217-6.

## P37 Chondrocyte conditioned media influences TGFβ and BMP signaling pathways in both normal and juvenile idiopathic arthritis fibroblast-like synoviocytes

### Amanda R. Schlefman^1^, Megan M. Simonds^2^, Kathleen E. Sullivan^3^, Carlos D. Rose^1^ and AnneMarie C. Brescia^1^

#### ^1^Nemours/Thomas Jefferson University, Wilmington, DE, USA; ^2^Nemours Biomedical Research, Wilmington, DE, USA; ^3^Department of Pediatrics, University of Pennsylvania, Philadelphia, PA, USA

##### **Correspondence:** Amanda R. Schlefman

**Background**

Juvenile Idiopathic Arthritis (JIA) is complicated by localized growth disturbances including condylar bony hypertrophy. Fibroblast-like synoviocytes (FLS) are one of the main cell types in the joint lining and, based on their role in cartilage damage in adult rheumatoid arthritis, may play a role in the growth alteration seen in JIA. Our prior work has shown that JIA FLS (JFLS) have a chondrocyte-like phenotype, possibly due to dysregulated TGFβ and BMP signaling, which may contribute to bony overgrowth. This study was designed to investigate the influence of chondrocytes (Ch) on FLS, relative to TGFβ and BMP signaling.

**Methods**

Three JFLS samples were selected from our IRB-approved tissue and fluid repository. We purchased a normal Ch line and three control FLS (CFLS) cell lines. At confluence, the Ch culture media was replaced with fresh Ch growth media and Ch grew untreated for 48 hours. Confluent JFLS and CFLS cells were then trypsinized and resuspended in Ch conditioned media. After plating, cell media and cell lysates were collected at 6 and 24 hours. RNA was isolated from cell lysates and analyzed using GeneChip Whole Transcriptome Expression Analysis. Quantitative PCR (Q-PCR) was used to validate microarray findings. Ingenuity Pathway Analysis (IPA) software was used to identify significant differentially expressed genes in the TGFβ and BMP signaling pathways.

**Results**

Differentially expressed probe sets were identified among CFLS (1145 probe sets) and JFLS (847 probe sets), both exposed to Ch conditioned media, with an FDR of 5%. A set of 10 genes was chosen for validation from the following comparisons based on most significant p-values: CFLS 6 h vs. 24 h, and JFLS 6 h vs. 24 h. Q-PCR analysis confirmed that these genes were either upregulated or downregulated in CFLS (Figure 1A) or JFLS (Figure 1B), in accordance with what was seen on microarray.

Using gene lists generated in IPA for TGFβ and BMP signaling pathways, we identified 21 significant overlapping differentially expressed genes within our microarray comparisons (Figure 2A and 2B). SMAD6, SKI and BMPR1a were all upregulated over the time course in both CFLS and JFLS. SMAD9 and BMP1 expression increased by 2-fold and 1.5-fold, respectively, in CFLS at 24 h compared to 6 h. SOS1 had a 1.6-fold higher expression level in JFLS at 24 h compared to 6 h. SMAD3 was downregulated over time in both CFLS and JFLS. BMPR1b and TGFβ3 expression decreased by 1.5-fold and 2-fold, respectively, in CFLS at 24 h compared to 6 h. SMURF1 had a 1.3-fold lower expression level in JFLS at 24 h compared to 6 h.

**Conclusion**

Ch influence TGFβ and BMP signaling in FLS in vitro. Hypertrophic Ch lead to bony overgrowth. Significant differences in gene expression were identified when comparing 6 h and 24 h samples from CFLS and JFLS exposed to Ch conditioned media. Upregulation of inhibitors such as SMAD6 and SKI suggest an overall decrease in canonical TGFβ signaling. Downregulation of SMAD3 and TGFβ3 also support this conclusion. Higher expression of SMAD9 and BMP1 suggest that BMP signaling is favored. SOS1, a member of the non-canonical TGFβ signaling pathway, is of interest as it was significantly expressed in JFLS.

**Ethics Approval**

This study was approved by Nemours Institution’s Internal Review Board, approval #2001-013.

**Fig. 1:**
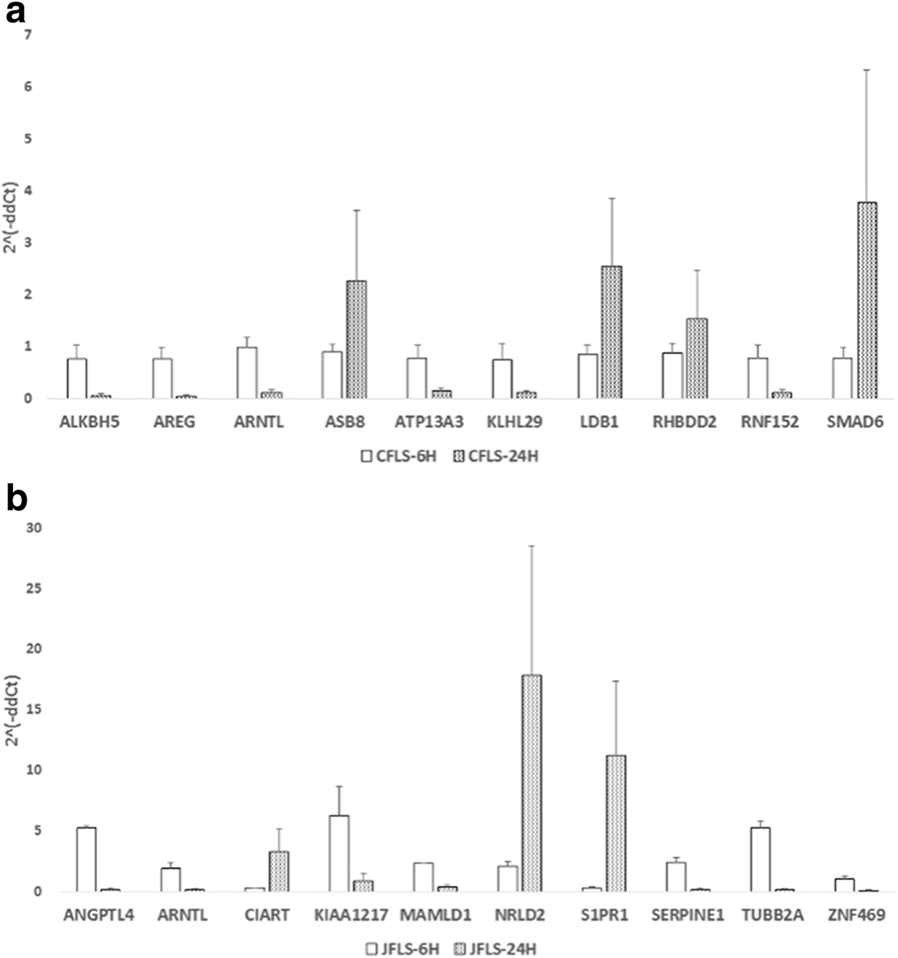
**(abstract P37).** Genes selected for validation by Quantitative PCR from microarray analyses. Top 10 significantly expressed genes, based on p-values, from (A) 6 h and 24 h CFLS samples exposed to Ch conditioned media and (B) 6 h and 24 h JFLS samples exposed to Ch conditioned media.

**Fig. 2:**
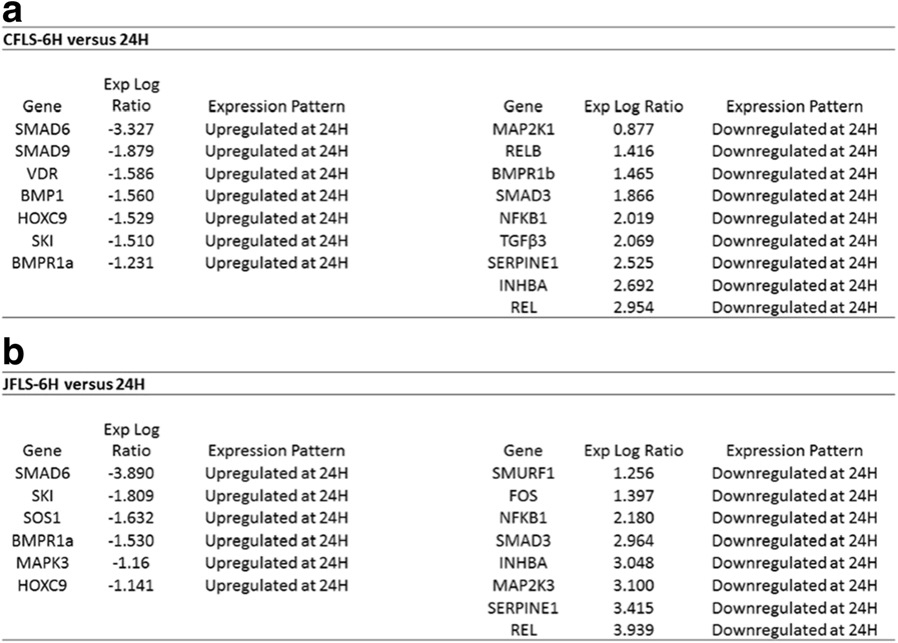
**(abstract P37).** Differentially expressed genes derived using IPA software from (A) CFLS and (B) JFLS, both exposed to Ch conditioned media over 24 h, in the TGFβ and BMP signaling pathway.

## P38 Comparison of pediatric localized scleroderma (LS) to healthy pediatric controls utilizing RNA sequencing for differentially expressed genes (DEGs)

### Roosha Mandal^1,2^, Emily Mirizio^1^, Qi Yan^1^, William Horne^1^, Kaila Schollaert-Fitch^1^, Kathryn S. Torok^1^

#### ^1^University of Pittsburgh, Pittsburgh, PA, USA; ^2^Carnegie Mellon University, Pittsburgh, PA, USA

**Background**

Pediatric localized scleroderma (LS) is a disfiguring autoimmune disease of the skin and underlying tissue. Smoldering disease activity leads to fibrosis and atrophy, causing physical and psychological disability that continues throughout childhood into adulthood. Available therapies for LS have had variable effects and are associated with morbidity themselves. A better understanding of the pathophysiology of LS, especially during the active inflammatory phase, would lead toward more directed and efficacious therapies.

RNA sequencing (RNAseq) technology provides a new approach for investigating disease operation and cellular influence by probing the genetic expression seen in direct samples. This technique also provides utility for existing paraffin-embedded skin tissue from patients that can be used to extract RNA for the investigation of differential gene expressions (DEGs) relating to the immunophenotype of the disease.

**Methods**

RNAseq was performed on skin (n = 3 LS, n = 1 Healthy Child) and corresponding peripheral blood (n = 2 LS, Healthy Child =1) samples. Fastq files (4 reads per sample) from sequencing were analyzed via CLC Genomics software identifying DEGs between groups by value of 2.5 log_2_fold change and p-values < 0.05. String-db opensource database networks and Cytoscape networks were generated based on probable significant gene lists resulting from Cluster 3.0 hierarchical clustering and CLC Genomics analyses.

**Results**

Genes of interferon inducible cytokines such as CXCL9, CXCL10, CXCL11, as well as IFN-γ itself, were significantly upregulated with an absolute log_2_fold >2.5 in both the peripheral blood and skin of LS subjects compared to healthy (*Figure 1*). String-db’s gene ontology (GO) organization indicated groupings of biological processes involved with the IFN-γ and T_H_1cellular response, such as CXCR3 receptor binding related CXCL9, CXCL10, and CXCL11, and T-helper 1 type immune response, showed absolute log_2_fold changes >7 in the LS skin samples and log_2_fold change values >2.55 in the peripheral blood compared to control.

**Conclusion**

In this pilot study of LS skin and peripheral blood RNA Seq analyses a signature of T_H_1 and IFN-γ - related genes were upregulated and were found to be networking with other genes associated with these biological processes, such as chemokine-mediated signaling pathway and regulation of T cell activation. These findings underscore the potential importance IFN-γ and T_H_1-related interactions in the skin and the blood of LS subjects. Evaluating a larger sample size and cross validating results from CLC genomics in R is underway.

**Fig. 1:**
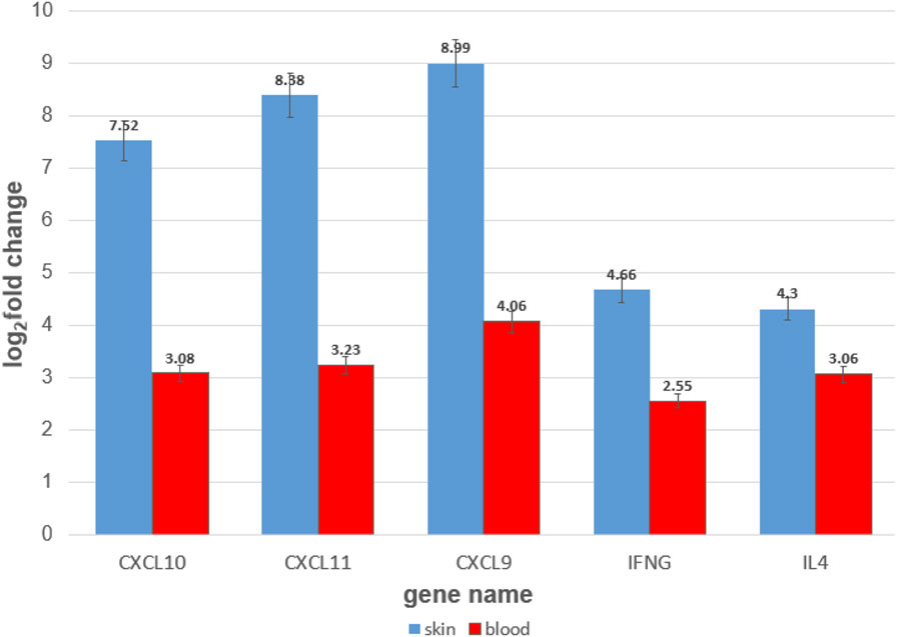
**(abstract P38).** Log_2_fold change comparison for IFN-γ and TH-related DEGs in LS blood vs. LS skin

**Acknowledgements**

This study received funding from the Nancy Taylor Foundation for Chronic Diseases Inc. and the Children’s Hospital of Pittsburgh Pediatric Scleroderma Fund.

## P39 High dimensional single-cell mass cytometry demonstrates distinct immune phenotypic and functional signatures in pediatric SLE and malaria patients

### Ryan Baxter, Josselyn Garcia Perez, Daniel Kong, Sidney Ogolla, Brianne Coleman, Conner Jackson, Xuhong Zhang, Debashis Ghosh, John Cambier, Rosemary Rochford, Elena Hsieh

#### University of Colorado Denver, Aurora, Colorado, USA

##### **Correspondence:** Ryan Baxter

**Background**

A long-standing paradox is the reduced incidence of autoimmune diseases in areas of the world where parasitic diseases are common. In both disorders, SLE and malaria, patients are exposed to chronic antigen stimulation and both develop similar immunophenotypes, with increased frequency of atypical and transitional B cells, hypergammaglobunemia, the presence of autoantibodies, and a robust systemic inflammatory response. However, mechanisms as to why chronic antigen exposure leads to the development of autoimmunity in SLE versus autoantibodies alone in malaria are not understood. We applied mass cytometry to evaluate the immune phenotypic and functional differences between children with autoantibodies and SLE, versus those with autoantibodies induced by malaria infection, to help elucidate what drives the transition to autoimmune disease.

**Methods**

Peripheral whole blood samples from 12 pediatric SLE (new diagnosis, flare, and inactive states) from the US, and 12 pediatric malaria patients from Kenya (acute infection and convalescent period) were analyzed by mass cytometry to simultaneously assess surface marker expression (phenotype) and cytokine production (function). Healthy age and gender-matched control samples from the US and Kenya were also similarly analyzed. All human donors were enrolled under a study protocol approved by the Institutional Review Board of the Research Compliance Office at the University of Colorado.

**Results**

CD14^hi^ monocytes from every pediatric SLE patient studied expressed a unique cytokine signature characterized by increased levels of monocyte chemoattractant protein-1 (MCP1), macrophage inflammatory protein-1β (Mip1β), and interleukin-1 receptor antagonist (IL-1RA), compared to malaria-infected patients, and age and gender-matched healthy controls. Pediatric malaria-infected patients demonstrated an expansion of T follicular helper cells and effector memory T cells during acute infection timepoints only, compared to pediatric SLE patients and age and gender-matched healthy controls. These distinct and unique immune phenotypic and functional signatures were conserved between children of the same disease group, suggesting that genetic predisposition (risk alleles) rather than environmental variation programs the response profile to chronic antigen stimulation.

**Conclusion**

These tightly regulated immune phenotypic and functional signatures provide clues into specific pathways that lead to the development of autoantibodies in autoimmunity versus autoantibodies induced by infection. This experimental approach can be applied to uncover immune dysregulation defects in autoimmunity and infection.

## P40

This abstract is not included here as it has already been published.

## P41 Variations in zinc finger protein gene might impact expression of transient receptor potential channel and receptor tyrosine kinase genes contributing to inflammasome dependent inflammatory response and development of clavicular cortical hyperostosis

### Lovro Lamot^1,2^, Kristina Gotovac^1^, Antonela Blažeković^1^, Danka Grčević^1^, Mirta Lamot^2^, Mandica Vidović^2^, Fran Borovečki^1^, Miroslav Harjaček^1,2^

#### ^1^University of Zagreb School of Medicine, Zagreb, Croatia; ^2^Clinical Hospital Center Sestre Milosrdnice, Zagreb, Croatia

##### **Correspondence:** Lovro Lamot

**Background**

Unilateral painful swelling of the clavicle might be the first symptom of chronic nonbacterial recurrent multifocal osteomyelitis (CNO/CRMO), but when lacking other inflammatory sites and recurrence, it might also be regarded as clavicular cortical hyperostosis (CCH), separate condition of unknown etiology. To elucidate underlying mechanisms of this disorder we performed various genome, transcriptome and proteome analysis.

**Methods**

Total RNA was isolated from whole blood of 18 new-onset CCH patients and 8 healthy controls, while DNA was extracted from three patients. Targeted exome sequencing was performed using the Nextera Rapid Capture Exome Kit (Illumina). The extracted variants were annotated and filtered using the Variant Studio software (Illumina) and Variant Interpreter (Illumina). DNA microarray gene expression was performed in five CCH and four control patients along with bioinformatical analysis of retrieved data. Carefully selected differentially expressed genes (TRPM2, TRPM3, TRPM7, CASP2, MEFV, STAT3, EIF5A, ERBB2, TLR4, NLRP3, CD24, MYST3) were additionally analyzed by qRT-PCR in all participants. In one patient, the blood cells were processed with cytosine for immunofluorescence microscopy (IF) using TRPM3 and TRPM7 antibody.

**Results**

Exome sequencing identified 428 shared identical variants among affected individuals. Thirty of these variants were not associated with a gene, 121 were in ZNF717 gene and 277 were distributed in 63 other genes. One heterozygous variant in CTBP2 gene, one in HYDIN gene and six in ZNF717 were classified as likely pathogenic. Microarray results and bioinformatical analysis revealed 974 differentially expressed genes, while qRT-PCR analysis showed significantly higher expression of TRPM3 and TRPM7, and lower expression of ERBB2. IF showed high signal of TRPM3 in blood cells of CCH patient.

**Conclusion**

Broad investigation of genetic contribution in CCH patients revealed likely pathogenic variants in ZNF717 gene which encodes a Kruppel-associated box (KRAB) zinc-finger protein from the large group of transcriptional regulators and probably influences observed gene expression patterns. Diligent analysis of those patterns indicated majority of differentially expressed genes were involved in various inflammatory processes, while further investigation confirmed aberrant expression in additional number of patients. Most significant expression divergence was detected in TRPM3 and TPRM7 genes which encodes transient receptor potential channels that are multimodal sensors able to regulate inflammasome and ERBB2 gene which encodes widely expressed cell surface growth factor receptor capable to protect cells during inflammation. Moreover, TRPM3 protein was abundant in blood cells of CCH patient confirming the increased function of this gene. Consequently, our results along with the results of previous studies imply CCH could be a new (auto)inflammatory bone disease entity in which ZNF717 variations could contribute to TRP channel overexpression and abundancy, inflammasome activation and reduced protection during inflammation.

**Ethics Approval**

The study was approved by Clinical Hospital Center Sestre Milosrdnice Institutional Review Board (IRB).

## P42 Transcription factor set binds multiple juvenile idiopathic arthritis genetic susceptibility loci

### Halima Moncrieffe^1,2^, Leah C. Kottyan^1,2^, Xiaoting Chen^1^, Mario Pujato^1^, John B. Harley^1,2,3^, Matthew T. Weirauch^1,2^ and Susan D. Thompson^1,2^

#### ^1^Center of Autoimmune Genomics and Etiology (CAGE), Cincinnati Children’s Hospital Medical Center, Cincinnati, OH, USA; ^2^Department of Pediatrics, University of Cincinnati College of Medicine, Cincinnati, OH, USA; ^3^US Department of Veterans Affairs Medical Center, Cincinnati, OH, USA

##### **Correspondence:** Halima Moncrieffe

**Background**

Juvenile idiopathic arthritis (JIA) genetic association studies have identified 17 loci (p<5×10^-8^) and 21 additional loci with suggestive evidence of association (p<1×10^-6^). As the majority of these genetic associations are located in non-coding regions of the genome, we tested the hypothesis that specific transcription factors (TFs) bind DNA at multiple JIA-associated genetic loci.

**Methods**

We defined a locus as those variants that had disequilibrium at r^2^>0.8 with the most significant genotyped marker at the locus. We assembled a large collection of publically available datasets, including 1,544 chromatin immunoprecipitation with next generation sequencing (ChIP-Seq) datasets, and “Active Chromatin” maps generated from combinations of histone marks in 221 different cell and tissue types. We developed a simulation test called RELI (Harley et al., Nature Genetics, in press) to assess the statistical significance of the number of JIA loci that intersect with each ChIP-seq dataset. Bonferroni corrected P-values (p_c_) were estimated for each ChIP-seq dataset evaluated. We focused our analysis on TF data sets where DNA was bound in ≥3 JIA genetic loci and p_c_<0.01. The ToppGene web portal was used to characterize functional annotations and protein interactions between TFs.

**Results**

Forty-three JIA loci were defined in this analysis. 129 of 1,544 ChIP-seq datasets, containing 63 unique TFs, had DNA bound in ≥3 JIA genetic loci and had p_c_<0.01. Cell populations identified in these 129 ChIP-seq datasets implicate several immune cell types in JIA pathogenesis, including CD4+ T cell subsets such as Th17, CD8+ T cells, CD56+ NK cells, and B cells. Ten TFs identified have a role in Th17 cell differentiation (p_c_ <10^-8^). The top result involves NFKB1 binding at 20/43 (46.5%) of JIA loci (p_c_<10^-14^). Two vitamin D receptor (VDR) ChIP-seq datasets are also highly significant (p_c_<10^-10^) and each intersects with at least 6 JIA loci. The viral TF Epstein-Barr nuclear antigen 2 (EBNA2) intersects with 17/43 (39.5%) JIA loci.

**Conclusion**

These data are consistent with common transcriptional control mechanisms operating across multiple JIA risk loci in a shared intracellular environment. Notably, most GWAS loci have small odds ratios (usually <1.2). If there are coordinated mechanisms across loci that alter disease risk with larger effect sizes, including viral TFs, then shared gene regulatory mechanisms such as these are important in generating disease risk.

## P43 Anti-transthyretin antibody positivity in pediatric patients with rheumatic disease

### Janet E. Orrock^1^, Cristina C. Clement^2^, Marco Vigano^3^, Tamar B. Rubinstein^1^, Laura Santambrogio^2^

#### ^1^The Children’s Hospital at Montefiore, Bronx, NY, USA; ^2^Albert Einstein College of Medicine, Bronx, NY, USA; ^3^Istituto Ortopedico Galeazzi, Milan, Italy

##### **Correspondence:** Janet E. Orrock

**Background**

Juvenile idiopathic arthritis (JIA) is composed of 7 subtypes, differentiated by clinical characteristics and serologic markers; however, there is much unknown about pathophysiology, and subtypes currently classified under the umbrella of JIA may be distinct diseases. This supposition is particularly relevant to systemic JIA (SJIA), a disease process with autoinflammatory, instead of autoimmune, features.

Transthyretin (TTR) is a serum protein implicated in the pathogenesis of amyloid diseases such as senile systemic amyloidosis. TTR functions normally as a transport protein in a homotetramer conformation. Each monomer is rich in β-sheet structures, which can lead to misfolding and aggregation, increasing immunogenicity. Preliminary investigations of TTR in adults with rheumatic disease have found evidence of anti-TTR antibody positivity in patients with rheumatoid arthritis (RA) and osteoarthritis (OA). There has been only 1 prior study of anti-TTR antibody positivity in juvenile arthritis, prompting our current investigation into patients with JIA, including the subgroups of SJIA versus all other types of JIA, and pediatric systemic lupus erythematosus (pSLE).

**Methods**

Serum samples were obtained from pediatric rheumatology clinic patients as well as from 2 biorepositories. There were a total of 45 patients with SJIA, 29 patients with other JIA, 36 patients with pSLE, 32 adults with osteoarthritis, and 11 healthy pediatric controls.

Anti-TTR antibody levels were measured by ELISA. Data were normalized to account for total serum protein levels, reported in optical density (OD) per milligram (mg) of protein. Statistical analyses comparing expression of anti-TTR antibody between the different groups were performed using the ordinary one-way ANOVA followed by Tukey multiple comparison test. A *p* value less than 0.05 was considered significant.

**Results**

Anti-TTR antibody levels were most elevated in the other JIA group (Figure 1). There were statistically significant differences between other JIA versus SJIA groups, as well as other JIA versus SLE groups and other JIA versus healthy controls groups. Compared to healthy controls group, there was no significant difference of anti-TTR antibody level for SJIA group.

**Conclusions**

Anti-TTR antibody levels were highest in the group of patients with JIA subtypes other than SJIA and had statistically significant differences when compared to the levels of patients with SJIA, pSLE, or healthy controls. This observation suggests an immune response to TTR may be part of the pathogenesis of some types of JIA. Further investigation with larger patient numbers of each JIA subtype is indicated to determine if anti-TTR antibody positivity may be a useful diagnostic and/or prognostic test.

**Fig. 1:**
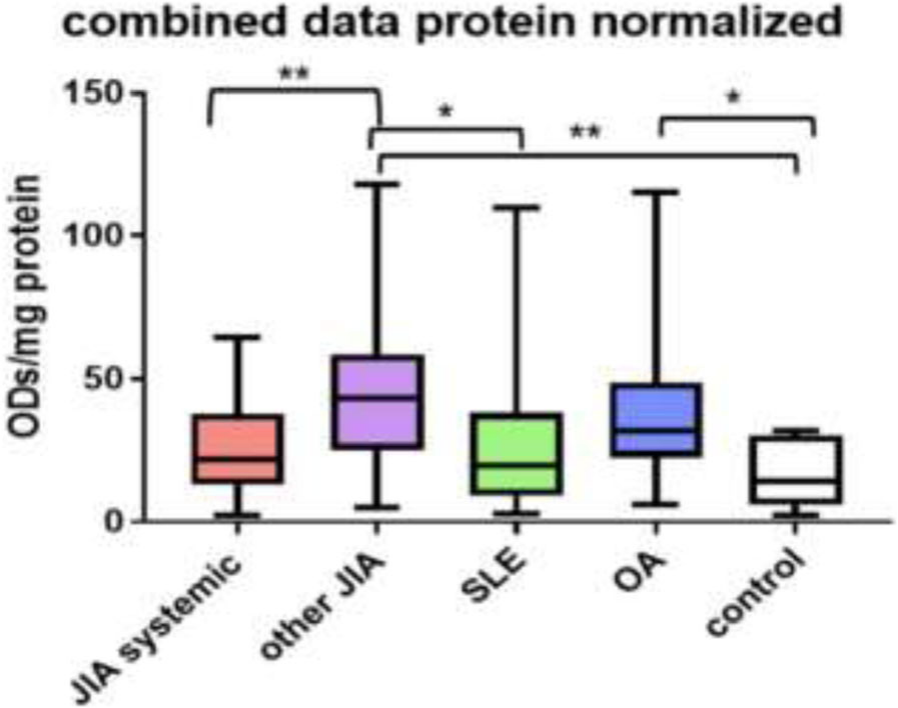
**(abstract P43).** Anti-TTR antibody levels normalized by protein. Data are shown as box plots. Lines inside the boxes represent the mean. Each box represents the standard error of measurement. Lines outside the boxes represent the minimum and maximum values detected. * means *p* less than 0.05. ** means *p* less than 0.005.

## P46 Juvenile idiopathic arthritis mediated by defective GATA-binding Protein 3

### Anna E. Patrick, T. Brent Graham, Thomas M. Aune, Jessica B. Duis

#### Vanderbilt University Medical Center, Nashville, TN, USA

##### **Correspondence:** Anna E. Patrick

**Background**

Juvenile inflammatory arthritis (JIA) is the most common inflammatory joint disease in childhood, yet its etiology and pathogenesis remain poorly understood. Genetic defects contribute to both pediatric and adult arthritis, including JIA and rheumatoid arthritis (RA). Previous studies suggest that immune regulatory genes play an important role in these conditions, particularly in early onset disease. GATA-binding protein 3 (GATA-3) is a key transcription factor that drives the differentiation of CD4+ T cells along the Th2 pathway. GATA-3 suppresses other cellular pathways, including the CD4+ Th1 and Th17 pathways. Importantly, patterns of CD4+ T cell function, including an increase in Th1 and Th17 and a decrease in Th2 pathways, have been implicated in various models of arthritis. Mutations in *GATA3* cause a syndrome of hypoparathyroidism, sensorineural deafness, and renal dysplasia (HDR syndrome) due to loss of GATA-3 function. In genome wide associated studies, *GATA3* has an association with RA and displays altered levels of transcription in JIA. We report the novel case of a frameshift mutation causing stop-gain and elongation of the C-terminus of the protein. Our work indicates a previously undescribed mechanism of JIA.

**Methods**

Whole exome sequencing was performed on a clinical basis by GeneDx. Peripheral blood mononuclear cells (pBMCs) were collected from the index patient and controls. Effector Th1, Th2, and Th17 cells were produced by polarization and re-stimulation of pBMCs. RNA was assessed by preparation of cDNA and analysis by polymerase chain reaction (PCR), Sanger sequencing, and quantitative PCR. Western blot and ELISA were used to assess intracellular and secreted proteins respectively. A mammalian expression vector containing GATA-3 was altered by site-directed mutagenesis to make mutant GATA-3. Constructs were expressed in Jurkat cell culture. The expressed GATA-3 or mutant ability to bind a GATA site was examined by luciferase assay.

**Results**

Our index case has c.1201_1202delAT: p.Met401ValfsX106 in exon 6 of *GATA3.* The patient has mutant GATA-3 that is expressed at both at the RNA and protein level, indicating it can escape nonsense-mediated decay. In an *ex vivo* assay, the index patient has polarized pBMCs that have decreased Th2 effector activity and increased Th1 and Th17 effector activity. In an *in vitro* expression assay the mutant GATA-3 is less able to drive GATA-dependent luciferase expression.

**Conclusions**

We find that dominant-negative mutations in *GATA3* cause an expanded spectrum of disease that includes inflammatory arthritis, and in this case psoriatic JIA. *GATA3* is a key regulatory gene in immune cells. A reduction in GATA-3 activity by aberrantly produced protein interferes with normal function causing dysregulation of T cell effector function, including a decrease in Th2 activity with a concomitant increase in Th1 and Th17 activity. This case demonstrates a link of GATA-3 function to psoriatic JIA. Moreover, these findings reveal a potential role of GATA-3 in the pathogenesis of autoimmune disease. Finally, this work suggests GATA-3 as a novel therapeutic target for patients with JIA or RA.

**Ethics Approval**

The study was approved by the Vanderbilt University Institutional Review Board.

## P45 Identification of optimal subcutaneous doses of tocilizumab in children with systemic juvenile idiopathic arthritis

### Hermine Brunner^1^, Nicolino Ruperto^2^ Daniel Lovell^1^, Gerd Horneff^3^, Maria Luz Gámir Gámir^4^, Markus Hufnagel^5^, Joy Hsu^6^, Min Bao^6^, Wendy Douglass^7^, Navita L. Mallalieu^6^, Chris Wells^7^, Christopher M. Mela^7^, Fabrizio de Benedetti^8^

#### ^1^Pediatric Rheumatology Collaborative Study Group (PRCSG), Cincinnati Children’s Hospital Medical Center, Cincinnati, OH, USA; ^2^Pediatric Rheumatology International Trial Organization (PRINTO), Istituto Giannina Gaslini Pediatria II - Reumatologia, Genoa, Italy; ^3^Asklepios Clinic Sankt Augustin, Sankt Augustin, University Hospital of Cologne, Cologne, Germany; ^4^Rheumatology, Hospital Ramon y Cajal Unidad de Reumatologia Pediatrica, Madrid, Spain ^5^Universitätsklinikum Freiburg, Freiburg, Germany. ^6^Roche Innovation Center, New York, NY, USA; ^7^Roche Products Ltd, Welwyn Garden City, United Kingdom; ^8^IRCCS Ospedale Pediatrico Bambino Gesù, Rome, Italy for the PRINTO and PRCSG Investigators

##### **Correspondence:** Fabrizio de Benedetti

**Background**

The efficacy and safety of intravenous (IV) tocilizumab (TCZ) were demonstrated in patients with systemic juvenile idiopathic arthritis (sJIA) in the phase 3 TENDER study^1^ (WA18221). Study WA28118 (ClinicalTrials.gov, NCT01904292) investigated dosing regimens of subcutaneous (SC) TCZ in patients with sJIA by bridging to data for IV TCZ to identify the optimal SC regimen.

**Methods**

This was a phase 1b multicenter, open-label study to evaluate the pharmacokinetics (PK), pharmacodynamics (PD), and safety of TCZ SC in patients aged 1-17 years with sJIA and inadequate response to glucocorticoids and nonsteroidal anti-inflammatory drugs. Interim analysis was conducted after 24 patients had received TCZ SC for 14 weeks. Patients could be TCZ naive or could switch from TCZ IV to SC. TCZ SC was administered for 52 weeks according to body weight: patients <30 kg received either 162 mg every 10 days before interim analysis or 162 mg every 2 weeks (Q2W) after interim analysis; patients ≥30 kg received 162 mg every week (QW). The study objective was to characterize the PK, PD, and safety of TCZ SC in patients with sJIA; efficacy was an exploratory objective.

**Results**

In total, 51 patients were enrolled, including 25 weighing <30 kg (8 before and 17 after interim analysis) and 26 weighing ≥30 kg. Twenty-six patients (51%) were TCZ naive and 25 (49%) switched from TCZ IV. Median steady state C_min_ was similar for patients <30 kg receiving TCZ 162 mg Q2W and those ≥30 kg receiving TCZ 162 mg QW, and the range largely overlapped (Table 1). More than 95% (49/51) of patients treated with TCZ SC had model-computed steady state C_min_ higher than the 5th percentile achieved with TCZ IV. The median and range of AUC_2weeks_ were similar for both body weight groups (Table 1). Changes in interleukin-6, C-reactive protein, and erythrocyte sedimentation rate were similar for both body weight groups. Almost all patients had ≥1 adverse event (AE; n = 50; 98.0%). Injection site reactions (ISRs) occurred in 21 patients (41.2%); most were mild, and none led to treatment interruption or withdrawal. The AE rate was 1200.3/100 patient-years (PY) (909.3/100 PY, excluding ISRs). The most common AEs were viral upper respiratory tract infection (13; 25.5%), neutropenia (13; 25.5%), and cough (12; 23.5%). Nine serious AEs occurred in 7 patients (13.7%; 19.3/100 PY); 5 were infections, all in the <30 kg group. Two deaths occurred, both in the <30 kg group. Median Juvenile Arthritis Disease Activity Score-71 improved (decreased) from baseline to week 52 for TCZ-naive patients (<30 kg, –13.9; ≥30 kg, –12.4) and was maintained or improved further for patients who switched from TCZ IV (<30 kg, –0.7; ≥30 kg –0.2).

**Conclusion**

A PK-based strategy was successful in bridging TCZ SC to TCZ IV in patients with sJIA. Dosing regimens of 162 mg Q2W in patients <30 kg and 162 mg QW in patients ≥30 kg provided adequate exposure to support efficacy comparable to that of TCZ IV. Except for ISRs, safety results were consistent with the known safety profile of TCZ in sJIA.

**Reference**

1. De Benedetti F et al. *N Engl J Med*. 2012;367:2385-2395.

**Table 1:** **(abstract P45).** See text for description

	TCZ SC (WA28118)		TCZ IV (WA18221)	
	<30 kg TCZ 162 mg Q2W n = 25	≥30 kg TCZ 162 mg QW n = 26	<30 kg TCZ 12 mg/kg Q2W n = 46	≥30 kg TCZ 8 mg/kg Q2W n = 43
Model-computed steady state PK parameters, median (range)				
C_min_,_ss_ μg/mL	64.2 (16.6-135.9)	72.4 19.5-157.8)	65.9 (19.0-135.5)	70.7 (5.3-126.6)
C_max_,_ss_ μg/mL	126.6 (51.7-265.8)	89.8 (26.4-190.2)	274.4 (148.8-444.0)	253.0 (119.6-404.3)
AUC_2weeks_,_ss_ μg/mL×day	1298 (539-2792)	1154 (334-2370)	1734 (840-2712)	1631 (526-2779)

## P46 Safety of tocilizumab in patients aged <2 years with active systemic juvenile idiopathic arthritis treated for one year

### Sunethra Wimalasundera^1^, Wendy Douglass^1^, Chris Wells^1^, Yukiko Kimura^2^, Carine Wouters^3^

#### ^1^Roche Products Ltd, Welwyn Garden City, United Kingdom; ^2^Hackensack University Medical Center, Hackensack, NJ, USA; ^3^University Hospital Gasthuisberg, Leuven, Belgium

##### **Correspondence:** Sunethra Wimalasundera

**Background**

The US Food and Drug Administration approved intravenous (IV) administration of tocilizumab (TCZ) for the treatment of patients ≥2 years of age with systemic juvenile idiopathic arthritis (sJIA) in 2011 based on results of the phase 3 TENDER study^1^ (WA18221). This approval was associated with a postmarketing requirement to investigate the use of TCZ in patients with sJIA <2 years of age (study NP25737; ClinicalTrials.gov, NCT01455701). Results from the 12-week main evaluation period (MEP) have been reported.^2^ Safety results following completion of the optional extension period (OEP) of NP25737 (until 52 weeks from baseline or 2 years of age, whichever was longer, was reached) are now reported.

**Methods**

NP25737 was a multicenter, open-label, single-arm study to evaluate the pharmacokinetics and safety of IV TCZ 12 mg/kg every 2 weeks for 12 weeks in patients aged <2 years with active sJIA for ≥1 month whose treatment with corticosteroids and nonsteroidal anti-inflammatory drugs failed and who were receiving stable background therapy. After the 12-week MEP, patients could participate in the OEP and continue TCZ treatment (without requirement for stable background therapy) to evaluate long-term safety. Cumulative adverse events (AEs) over the entire study period are reported.

**Results**

Seven of 11 patients enrolled in the MEP continued to the OEP and received ≥1 dose of TCZ. Across the entire study period (n = 11), the median number of TCZ doses was 11.0 (range, 2-26), and the median duration of exposure to TCZ was 22.1 weeks (range, 4.1-58.1). Most patients (10/11; 90.9%) had ≥1 AE; most AEs were mild or moderate in intensity and unrelated to study drug. The most common AEs were upper respiratory tract infection (6/11 patients; 54.5%), followed by hypersensitivity, neutropenia, rash, viral upper respiratory tract infection, and vomiting (each in 3/11 patients; 27.3%). Seven serious AEs occurred in 5 of 11 patients (45.5%), of which 2 occurred during the OEP (transaminases increased and histiocytosis hematophagic), 3 occurred during the MEP (3 hypersensitivity events), and 2 occurred during the safety follow-up of the MEP (sJIA flare and hand-foot-and-mouth disease). AEs leading to dose modification occurred in 5 of 11 patients (1 in the MEP and 4 in the OEP) mostly because of infections, neutropenia, and elevated liver enzymes, all mild or moderate in intensity. AEs leading to withdrawal occurred in 5 of 11 patients (45.5%): during the OEP, 1 patient was withdrawn because of a serious AE of increased transaminases; during the MEP, 3 patients were withdrawn because of serious hypersensitivity reactions to TCZ, and 1 patient was withdrawn because of thrombocytopenia. No deaths were reported during the study. AE rates per 100 patient-years of exposure are reported in Table 1.

**Conclusion**

During the OEP of the study, long-term treatment with TCZ was well tolerated in sJIA patients aged <2 years, and no additional safety signals were reported in the OEP beyond those reported in the MEP or observed previously for patients with sJIA aged >2 years.

**References**

1. De Benedetti F et al. *N Engl J Med*. 2012;367:2385-2395.

2. Mallalieu NL et al. *Arthritis Rheumatol*. 2017;69(suppl 10):abstract 2856.

**Table 1:** **(abstract P46)**. Rates of AEs

	TCZ 12 mg/kg IV every 2 weeks		
	Main Evaluation Period n = 11	Optional Extension Period n = 7	Entire Study Period n = 11
Total patient-years at risk	2.3	5.1	7.4
Number of events (AE rate per 100 patient-years at risk)			
Any AE	32 (1396.4)	47 (926.5)	79 (1072.7)
Serious AE	5 (218.2)	2 (39.4)	7 (95.1)
AE with fatal outcome	0	0	0
AE leading to withdrawal	4 (174.6)	1 (19.7)	5 (67.9)
AE leading to dose interruption	1 (43.6)	12 (236.5)	13 (176.5)

## P47 Systemic Treatment of Uveitis in Children A Single Center Experience

### Khaled Alsaeid^1,2^, Jasim Alfailakawi^2^, Hamid Alenezi^2^, Hazem Alsaeed^2^

#### ^1^Kuwait University, Kuwait; ^2^Mubarak Alkabeer Hospital, Jabriya, Kuwait

**Background**

Uveitis is a condition frequently seen in children with juvenile idiopathic arthritis (JIA). A proportion of children with uveitis do not respond to local treatment with corticosteroid eye drops. Because of the risk of vision loss associated with uveitis and the complications of prolonged local steroid treatment, patients with severe uveitis are referred to the pediatric rheumatologist for systemic therapy. Traditional systemic treatments may not be effective, are slow in onset of action (traditional DMARDs) or are associated with unacceptable toxicity profile (corticosteroids). TNF alfa antagonists have been found of benefit in uveitis. Adalimumab has been recently licensed in our country for this indication. Herein we report our experience in treating uveitis in the setting of a referral pediatric rheumatology center.

**Methods**

The files of children with uveitis referred from ophthalmology centers across Kuwait were reviewed. All patients had systemic therapy with traditional DMARDs, corticosteroids or biologic DMARDs (anti-TNF). All patients had bi-weekly to 4 weekly visits to the ophthalmologist during therapy. Degree of improvement or worsening was reported in percentage of change in Standardization of the Uveitis Nomenclature (SUN) classification criteria. All children had a complete blood count with liver and renal function tests every 6-8 weeks. All patients treated with a biologic agent had T-spot test prior to starting treatment to rule out latent tuberculosis.

**Results**

20 patients with uveitis were included in this review. The underlying disease in 11 patients was JIA, one patient had Vogt-Koyonagi-Harada syndrome and 8 patients had idiopathic uveitis. Four patients were excluded due to missing data. The mean age of patients at diagnosis was 11 years. Six patients were treated with a traditional DMARD alone (systemic corticosteroids, methotrexate, cyclosporine and or mycophenylate), 3 patients with adalimumab monotherapy and 7 patients were treated with adalimumab and a DMARD. One patient with JIA on etanercept was switched to adalimumab once uveitis had developed. Overall, 75% improvement after 3 months and 93.3% after 9 months was reported. Signs of active uveitis were more likely to improve at 3 months and 9 months in patients treated with adalimumab (monotherapy or combined with a DMARD) than patients on DMARDs (78% and 100% improvement vs 66% and 81%). No serious adverse events or tuberculosis developed during the study period.

**Conclusion**

Systemic treatment of uveitis refractory or dependent on high dose local corticosteroids treatment is warranted and appears to be effective. Adalimumab appears to be more effective than conventional DMARDs and safe in treating uveitis in children. Our experience is consistent with the results of the Sycamore trial. The cooperation between pediatric ophthalmologists and rheumatologists and early referral is paramount.

**Ethics Approval**

IRB Approval obtained reference number VDR/EC/3200, Kuwait University.

## P48 Resilience and mental illness in children with arthritis in a national sample of US youth

### Tamar Rubinstein^1^, Danielle R. Bullock^2^, Kaveh Ardalan^3^, Wenzhu Mowrey^1^, Nicole Brown^1^, Ruth E. Stein^1^

#### ^1^Albert Einstein College of Medicine/Children’s Hospital at Montefiore, Bronx, NY, USA; ^2^University of Minnesota/Masonic Children’s Hospital, Minneapolis, MN, USA; ^3^Northwestern University Feinberg School of Medicine/Ann & Robert H. Lurie Children’s Hospital of Chicago, Chicago, IL, USA

##### **Correspondence:** Tamar Rubinstein

**Background**

Resilience is the ability to recover from adversity or chronic stress and is associated with psychological health in children with chronic illness. Emerging data show that chronic stress may contribute to rheumatologic diseases and that children with these diseases are particularly at risk for mental illness. However, the role resilience plays in youth with rheumatologic diseases has not been examined. Our objective was to investigate the relationship between resilience and mental illness among children with arthritis.

**Methods**

Using data from the 2016 National Survey of Children’s Health (NSCH), we investigated differences in personal and family resilience between children with arthritis, children with other chronic acquired physical illnesses (CAPC)*, and children with neither. The NSCH is a survey of sampled households with children <18 years conducted by the National Center for Health Statistics at the Centers for Disease Control. Children were determined to have current conditions, including arthritis, if their guardian indicated yes to both “has a doctor or other health care provider ever told you that this child has…” and “does this child currently have the condition?” We defined personal resilience as high/moderate/low by responses of ‘definitely true’/‘somewhat true’/‘not at all true’ to the following questions: does the child ‘bounce back quickly when things do not go his or her way?’ (for children < 6 years old); or, do they ‘stay calm and in control when faced with a challenge?’ (for children 6 years or older). Family resilience was scored (range: 0-4) based on positive responses to the following query: when faced with a problem do they 1) talk together 2) work together 3) know their strengths 4) stay hopeful. Higher scores were indicative of greater resilience. Bivariate analyses examined differences between the groups. We investigated whether resilience was inversely associated with comorbid depression/anxiety among children with arthritis in logistic regression models.

**Results**

Among 50,212 children included in the survey, 16,892 had CAPC and 138 had current arthritis. There were fewer children with arthritis and high personal resilience compared to children with other CAPC and children with neither (44% vs 51%, 61%, p<0.001). Children with arthritis had the lowest mean family resilience scores (1.5 vs 1.8, 2.0, p=0.0001). More children with arthritis had anxiety/depression (34% vs 13%, 6%, p<0.001). Personal resilience, but not family resilience, was inversely associated with anxiety/depression among children with arthritis. A graded relationship was seen between the level of personal resilience and the odds of having anxiety/depression, which persisted after adjusting for age, sex, minority race/ethnicity, and poverty (Table 1).

**Conclusions**

Children with arthritis exhibit lower personal and family resilience compared to peers. Lower personal resilience is associated with higher odds of anxiety/depression among children with arthritis. Further studies will help us understand whether resilience building interventions may improve mental health among children with arthritis.

*Asthma, allergies, diabetes, and epilepsy.

**Table 1:** **(abstract P48).** Odds Ratios (OR) for Anxiety/Depression by Resilience Level in Children with Arthritis

	Unadjusted			Adjusted**		
Resilience Level*	OR	Confidence Interval	p value	OR	Confidence Interval	p value
High	1	---	---	1	----	----
Moderate	3.13	1.40 – 7.01	0.005	2.69	1.13 – 6.41	0.025
Low	4.57	1.29 – 16.1	0.018	5.16	1.32 – 20.14	0.018

* Personal resilience defined in children <6 years old as ‘bounces back quickly when things do not go his or her way’ and in older children as ‘stays calm and in control when faced with a challenge’: ‘definitely true’ (high), ‘somewhat true’ (moderate), or ‘not true’ (low).

** Adjusted for age, sex, minority race/ethnicity, and poverty status.

**Ethics Approval**

Approval for exemption was obtained from the Einstein-Montefiore Institutional Review Board.

## P49 Does anti-cyclic citrullinated peptide antibody positivity predict erosive disease in polyarticular juvenile idiopathic arthritis jia patients? Experience from a tertiary care medical center in the United States

### Saumya V. Joshi^1^, Bipin Malla^1^, Yujuan Zhang^1^, Trevor Davis^1^

#### ^1^Department Pediatric Rheumatology, Floating Hospital for Children, Tufts Medical Center, Boston, MA, USA

##### **Correspondence:** Saumya V. Joshi

**Background**

The primary hypothesis of this study is that anti-CCP antibodies measured in the serum of subjects with polyarticular juvenile idiopathic arthritis(JIA) are associated with increased risk of bony erosions. We further hypothesize that anti- anti-cyclic citrullinated peptide (CCP) antibody positivity is an independent risk factor for severe bone involvement in both rheumatoid factor (RF) positive and RF negative polyarticular JIA.

**Methods**

Extensive chart review of Floating Hospital for Children’s electronic medical records (EMR) identified 58 subjects between January 2011 through December 2017 (7 years) with polyarticular RF positive or polyarticular RF negative JIA who had at least one RF and CCP value tested. Subjects with a diagnosis of enthesitis related arthritis, juvenile psoriatic arthritis, chronic unspecified arthritis or extended oligoarticular arthritis were excluded. Further EMR review within these subjects identified those with bony erosions on the available radiographs read by staff radiologists trained in pediatric musculoskeletal radiography. We defined positive CCP and RF above 20 units/ml and 15 units/ml respectively based on our lab standards. Subjects with either two consecutive positive values or a single positive value, not checked again, were considered positive for this study. Subjects with a single or multiple negative serum values or subjects with an initial positive but the subsequent negative CCP or RF were considered negative for this study. We evaluated the statistical significance of the association between CCP positivity and erosive disease as well as RF positivity and erosive disease.

**Results**

The mean subject age at the time of the chart review was 18.2 years-old, age range 4 to 58 years. 81% subjects were females and 76% white. About 28% subjects had erosive disease in all (16/58). Out of these 16 subjects, 10 had both RF and CCP negative, 4 had both positive, 1 subject had CCP positive but RF negative and another one had CCP negative but RF positive. Eight subjects were positive for anti-CCP antibodies, out of which 5 subjects had evidence of erosive bony disease on radiographs (p value significant at 0.03 on Fischer test). Anti-CCP antibodies had a specificity of 92.8% (CI 80.5% to 98.5%) and a sensitivity of 31.2% (CI 11.0% to 58.6%). Out of 10 RF positive subjects, 5 had erosions and 5 did not. Interestingly RF positivity was not significantly correlated with erosive disease (p value 0.1). However, with this small sample size, we would only be able to detect a difference of about 70% erosion rate between the two groups (Power 80%, 95% CI). RF had similar sensitivity (31.2%, CI 11.0-58.6) but poorer specificity (88.1%, CI 74.3-96.0) compared to anti-CCP antibodies.

**Conclusions**

This study provides preliminary evidence that CCP is a biomarker of more aggressive disease in JIA. These findings support inclusion of anti-CCP antibody positivity in the diagnostic criteria for polyarticular JIA in any future revisions of ILAR criteria, but these findings must be validated by future larger scale studies.

**Ethics Approval**

The study protocol underwent exempt review by the IRB.

## P50 Is the patient global health assessment reliable in juvenile idiopathic arthritis?

### Cindy M. Wang^1^, Rebecca Trachtman^1^, Jackie Szymonifka^2^, Karen B. Onel^1^

#### ^1^Hospital for Special Surgery/Weill Cornell Medicine, New York, NY, USA; ^2^Hospital for Special Surgery, New York, NY, USA

**Background**

Juvenile idiopathic arthritis (JIA) is a chronic autoimmune disease that poses many challenges in monitoring and management. There is increasing recognition of the importance of patient-reported outcomes (PROs), because they reflect the patient's own view of their rheumatic illness. Although newer PROs are being developed and more widely utilized both in clinical care and in research, their performance and reliability remain unclear.  This study seeks to evaluate: (1) performance of the patient global health assessment (PGA) compared to standard disease activity measures in children with JIA, (2) correlations of the PGA with socioeconomic status (SES) in children with JIA, and (3) relationship between PGA and physician global health assessment in children with JIA.

**Methods**

A convenience sample of patients with JIA (N = 47) aged 2-18 were recruited from a single center. Patients aged ≥ 10 years completed the questionnaire, and parents of patients aged 2-9 completed a proxy questionnaire on their child’s behalf. Correlations between (1) the PGA and disease activity, as measured by the Juvenile Disease Activity Score-71 (JADAS-71), (2) the PGA and physician global health assessment, and (3) the physician global health assessment and the JADAS-71 were evaluated using Spearman correlation coefficients. PGAs were compared by age, sex, insurance status, race, and ethnicity using Wilcoxon rank-sum tests. Differences between PGA and physician global health assessments were compared using Wilcoxon rank-sum tests.

**Results**

16 parents and 31 patients completed the patient global assessments (Table 1). There was a moderate correlation between PGA and JADAS-71 (r= 0.503, p<0.001), and PGA and physician global health assessments (r= 0.503, p= 0.002). There was a stronger correlation between physician global health assessments and JADAS (r= 0.612, p<0.001). PGA median scores and IQRs appeared to be higher among patients with Medicaid insurance, non-white race, and Hispanic ethnicity, with the greatest difference seen in the category of race (Table 2). There were no differences between patient and physician assessments across all groups except among patients with Medicaid (difference median =-1.25) and Hispanic patients (difference median = 2) (Table 3).

**Conclusion**

Our results demonstrate that physician global health assessment had a stronger correlation with standard disease activity measures than the PGA. These scores were higher in patients who were non-White race, Hispanic, and had Medicaid insurance; however, these were not statistically significant. These data indicate that the PGA is fairly stable across groups, and can be used reliably for disease monitoring. Further validation using data from other centers and patient cohorts is needed prior to implementation in routine clinical care.

**Table 1:** **(abstract P50).** Patient Characteristics

	Patients and Parents (N = 47)	Patients (n = 31)	Parents (n = 16)
Age, years (median, IQ range)	12.4 [8.6, 15.0]	14.2 [12.4, 15.8]	4.3 [3.3, 9.0]
Sex			
Male	17 (36.2%)	14 (45.2%)	3 (18.8%)
Race			
Caucasian/White	38 (80.9%)	24 (77.4%)	14 (87.5%)
Asian	3 (6.4%)	3 (9.7%)	0 (0.0%)
Other	6 (12.7%)	4 (12.9%)	2 (12.5%)
Ethnicity			
Hispanic	8 (17.0%)	5 (16.1%)	3 (18.8%)
Insurance			
Medicaid	8 (17.0%)	4 (12.9%)	4 (25.0%)
Private	39 (83.0%)	27 (87.1%)	12 (75.0%)
BMI, kg/m^2^ (median, IQ range)	18.3 [15.6, 22.3]	21.3 [18.2, 23.8]	15.5 [15.0, 16.8]

**Table 2:** **(abstract P50).** Comparison of Patient Global Assessments by age, sex, insurance status, race and ethnicity

	Patient global median [IQR]	P-value
Age		
Patient completed	2 [0, 5]	0.892
Parent completed	2 [0.05, 4.5]	
Sex		
Male	2 [0, 5]	0.679
Female	2 [0, 4]	
Insurance		
Medicaid	2.5 [1, 5,]	0.313
Private	2 [0, 4]	
Race		
White	1.5 [0, 4]	0.266
Non-white	4 [2, 5]	
Ethnicity		
Hispanic	2.5 [2.5, 5]	0.134
Non-Hispanic	1 [0, 4]	

**Table 3:** **(abstract P50).** Discordance of patient and physician assessments by age, sex, insurance status, race, and ethnicity

	Difference median** [IQR]	P-value
Age		
Patient completed	0 [-1, 2]	0.370
Parent completed	0 [-2.25, 1]	
Sex		
Male	0 [-2, 2]	0.731
Female	0 [-2, 1]	
Insurance		
Medicaid	-1.25 [-4.5, 1.0]	0.226
Private	0 [-2, 1]	
Race		
White	0 [-2, 1]	0.957
Non-white	0 [-2, 2]	
Ethnicity		
Hispanic	2 [-0.5, 2.25]	0.127
Non-Hispanic	0 [-2, 1]	

**Differences between patient and physician global assessments were calculated as patient global – physician global

**Ethics Approval**

This study was approved by the Hospital for Special Surgery IRB.

## P51 Stepping it up: the use of physical activity monitors as an outcome measure in juvenile myositis

### Emily Brunner^1^, Laura Tasan^1^, Kathryn S. Torok^1^, Kimberly Francis^1^, Emily Mirizio^1^, Kaila Schollaert-Fitch^1^, Chester Oddis^2^, Rohit Aggarwal^2^

#### ^1^Children’s Hospital of Pittsburgh of UPMC, Pittsburgh, PA, USA; ^2^UPMC, Pittsburgh, PA, USA

**Background**

Clinical trials in juvenile myositis (JM) are limited, partly until recently due to lack of validated longitudinal outcome measures. More robust, objective and continuous functional assessments should supplement currently validated outcome measures in JM. The use of physical activity monitors (PAM), which effectively quantify movement, may enhance assessment of disease activity and complement the current core set measures (CSM) as established by Pediatric Rheumatology International Trials Organization (PRINTO) and International Myositis Assessment and Clinical Studies Group (IMACS). We examined the use of variables obtained from a commercially available PAM device, Fitbit® One, as outcome measures in patients with JM.

**Methods**

JM patients from age 5-17 years were enrolled from rheumatology clinics with an ongoing enrollment. Following evaluations were performed in each patient at baseline, 1, 3 and 6 months: a) PRINTO and IMACS CSMs including Manual Muscle Testing (MMT) and Childhood Myositis Assessment Scale (CMAS), b) Patient/Parent Reported Outcome measures (PRO): Patient-Reported Outcome Measurement Information System Mobility Short Form (PROMIS-SF) and Childhood Health Assessment Questionnaire (CHAQ), and office functional tests: Sit-to-Stand (STS), Timed Up and Go (TUG) and Six Minute Walk Distance (6MWD). A waist-based Fitbit® One was worn by patients for 7 consecutive dates every month for 6 months and average daily step counts were assessed. Spearman’s correlation coefficient was used to evaluate the relationship between Fitbit® step counts as compared to current IMACS and PRINTO CSMs, PROs and functional tests. Patients were characterized as active and stable disease based on PRINTO criteria and MD assessment, and ability of Fitbit® average daily steps to differentiate between the two groups were assessed.

**Results**

As data collection and analysis are ongoing, the following results represents the cross-sectional analysis at baseline. A total of 17 out target of 25 JM participants have been enrolled, including 5 active and 12 stable, 76% female, 94% White with mean (SD) age of 11.5 years (3.4). Spearman’s correlation analyses demonstrated a moderate to strong correlation between Fitbit® average daily step counts and baseline MMT8, patient/parent global, PROMIS-SF and CHAQ (Table 1), but not with CMAS, MD Global and functional tests. Fitbit average daily step counts, most CSMs and physical functional variables were different between active vs. stable patients at baseline visit (Table 2), however, p values not calculated due to small sample size.

**Conclusion**

The number of steps recorded by Fitbit® in the free-living environment had moderate to strong correlation with key JM CSMs except CMAS, supporting further study of the potential for the use of the Fitbit® steps as outcome measures in JM. Continued analysis of longitudinal data will help to determine the utility of a commercially available PAM as an outcome measure in JDM.

**Funding**

This study received funding from Cure JM.

**Ethics Approval**

This study was approved by the Institutional Review Board at the Children’s Hospital of Pittsburgh of University of Pittsburgh Medical Center PRO16110410.

**Table 1:** **(abstract P51).** Spearman’s correlation analyses of Fitbit® steps in free-living environment with CSMs^1^, PROs^1^ and functional tests

	Average Fitbit® steps Rho (p value)
CHAQ^1^	-0.68 (0.007)
Patient/Parent Global	-0.49 ( 0.07)
PROMIS-SF^1^	0.49 ( 0.08)
MD global	-0.18 ( 0.5)
CMAS^1^	0.15 ( 0.6)
MMT8^1^	0.76 ( 0.002)
STS^1^	-0.07 ( 0.84)
TUG^1^	0.11 ( 0.71)
6MWD^1^	0.21 ( 0.44)

^1^Core Set Measures (CSM), Patient Reported Outcomes (PROs), Childhood Health Assessment Questionnaire (CHAQ), Patient-Reported Outcome Measurement Information System Mobility Short Form (PROMIS-SF), Manual Muscle Testing (MMT), Childhood Myositis Assessment Scale (CMAS), Sit-to-Stand (STS), Timed Up and Go (TUG), Six Minute Walk Distance (6MWD).

**Table 2:** **(abstract P51).** Baseline differences in CSM^1^ and functional tests in active versus stable patients. Median (IQR^1^)

Variables	Active (n=5) Median (IQR)	Stable (n=12) Median (IQR)
CHAQ^1^	0.63 (0.5 - 0.875)	0 (0 - 0.1875)
Patient/Parent Global	6 (6 - 6.5)	0.25 (0 - 1.125)
PROMIS SF^1^	29 (28 - 32)	32 (31.75 - 32)
MD Global	5.5 (3 - 6)	0 (0 - 0.065)
CMAS^1^	45 (35 - 49)	49 (46.75 - 51.25)
MMT8^1^	139 (133 - 146)	149.5 (147.5 - 150)
STS^1^	14 (14 - 16)	15.5 (13.75 - 18.25)
TUG^1^	7 (6 - 7)	7 (7 - 8)
6MWD^1^	368 (336 - 410)	403 (345.75 - 428)
Average Fitbit® Steps	4197.5 (3415.5 - 5744)	7135 (5460 - 8863)

^1^Core Set Measures (CSM), Interquartile Range (IQR), Childhood Health Assessment Questionnaire (CHAQ), Patient-Reported Outcome Measurement Information System Mobility Short Form (PROMIS-SF), Manual Muscle Testing (MMT), Childhood Myositis Assessment Scale (CMAS), Sit-to-Stand (STS), Timed Up and Go (TUG), Six Minute Walk Distance (6MWD).

## P52 Analyze myositis with ultrasound and exercise (AMUSE) kids - analysis of baseline data

### Laura Tasan^1^, Emily Brunner^1^, Rohit Aggarwal^2^, Chester Oddis^2^, Emily Mirizio¹, Kaila Schollaert-Fitch^1^, Kim Francis¹, Judith Squires^1^, Amisha Shah^1^, Kathryn Torok^1^

#### ^1^Children’s Hospital of Pittsburgh of UPMC, Pittsburgh, PA, USA; ^2^University of Pittsburgh Medical Center, Pittsburgh, PA, USA

##### **Correspondence:** Laura Tasan

**Background**

Pediatric Rheumatology International Trials Organization (PRINTO) and International Myositis Assessment and Clinical Studies Group (IMACS) have validated core set outcome measures (CSM) in Juvenile Myositis (JM). However, given some limitations of these CSMs, there is an unmet need for more dynamic yet objective outcome measures for JM. The aim of this pilot study is to test the reliability, validity and responsiveness of advanced ultrasound (US) modalities as dynamic imaging outcome measures in JM patients.

**Methods**

This prospective observational cohort study includes JM subjects consecutively recruited from a rheumatology clinic for the collection of clinical, functional and US data at baseline, 3 and 6 months follow up. Clinical and functional variables include: demographics, clinical characteristics, PRINTO/IMACS defined CSMs, Sit To Stand (STS), Time Up and Go (TUG), and 6 Minute Walk Distance (6MWD). US modalities to evaluate muscle consistency and perfusion include: Gray Scale with Echogenicity (EI) and Muscle thickness (MT), Power Doppler (PD), 2D Shear wave© Elastography (SWE) and Contrast Enhanced US with Lumason© (CEUS) performed on unilateral proximal (vastus lateralis) and distal muscle (medial gastrocnemius) groups. At each study visit, US is performed before and after all functional measures and exercise to stress the muscles of interest. Spearman’s correlation coefficient was utilized to evaluate the relationship of US measures with CSMs and functional tests, specifically using Manual Muscle testing (MMT8) as the primary CSM outcome for validation of US measures.

**Results**

We have enrolled 9 JM patients currently out of our goal of 20 JM patients and 10 healthy controls. Patients enrolled in this pilot study are 89% Non-Hispanic females with a mean age of 11.5 and a mean disease duration of 36 months. Several US measures had moderate to strong correlations with JM CSMs (MMT, MDAS, CMAS), including Echogenicity, Power Doppler, CEUS blood flow measurements (Time to Peak (T_p_) and Peak Intensity (I_p_)) and CEUS blood volume measurement (area under the curve (AUC)), both pre- and post-exercise, with most associations being stronger post-exercise (Table 1, r_s_ >0.35 bolded). Importantly, the US parameters EI, PD, CEUS, correlate moderately to strongly with the MMT8, the pre-defined primary outcome measure for this study.

**Conclusions**

Preliminary results demonstrate that US measures of Echogenicity, Power Doppler and CEUS (both blood flow and blood volume) correlate moderately to strongly with core JM outcome measures of functionality at baseline, supporting their potential to serve as supportive outcome measures in JM studies. The stronger correlations in the blood flow and blood volume CEUS variables post exercise likely reflect the increased blood perfusion in muscles, reflecting normal muscle function. Whereas the stronger negative correlation with Power Doppler reflects increased blood flow secondary to active muscle inflammation. Further longitudinal analysis is underway to examine which modality is more sensitive and specific to changes over time.

**Table 1:** **(abstract P52).** Baseline Spearman Correlations of US muscle measures, *Core Set Measures, and Functional

Test in all JM Patients (n=9)										
	*CHAQ SDI	*Pt/Parent Global	PROMIS SF Pt/Parent	*MDAS	*MD global	*CMAS	*MMT8	STS	TUG	6MWD
Echogencity Pre	0.30	0.30	-0.21	**0.38**	**0.45**	**-0.52**	**-0.84**	-0.17	**-0.43**	**-0.36**
Echogencity Post	0.34	0.34	-0.28	**0.45**	**0.50**	**-0.56**	**-0.84**	-0.10	**-0.43**	-0.30
Muscle thickness Pre	0.01	0.04	0.04	0.08	-0.08	0.32	-0.20	**-0.47**	0.08	0.10
Muscle thickness Post	-0.02	-0.01	-0.19	0.19	0.11	0.25	-0.19	**-0.54**	-0.10	0.28
Elastography Pre	0.05	0.03	-0.18	0.10	0.12	-0.24	0.27	-0.34	0.15	-0.34
Elastography Post	0.01	-0.04	**0.39**	0.29	0.20	-0.06	0.22	**-0.66**	0.05	-0.02
Power doppler Pre	0.29	0.29	**-0.45**	**0.41**	**0.42**	**-0.41**	**-0.41**	0.28	-0.22	0.14
Power doppler Post	0.00	0.00	0.00	0.33	**0.40**	-0.13	**-0.78**	-0.09	**-0.78**	0.00
CEUS T_p_ Pre	**-0.47**	**-0.51**	-0.20	-0.10	-0.06	0.17	**0.57**	-0.05	-0.22	**0.57**
CEUS T_p_ Post	-0.27	-0.29	-0.30	**-0.76**	**-0.60**	0.19	**0.43**	0.00	**0.55**	0.14
CEUS I_p_ Pre	-0.33	-0.33	0.04	0.21	0.16	0.00	**0.39**	0.15	**-0.49**	-0.04
CEUS I_p_ Post	0.09	0.09	0.27	**0.71**	**0.70**	-0.09	-0.09	-0.29	**-0.68**	0.03
CEUS AUC Pre	-0.15	-0.15	0.10	**-0.36**	-0.29	-0.11	-0.11	0.33	0.16	0.07
CEUS AUC Post	-0.09	-0.09	-0.27	**-0.37**	**-0.43**	-0.26	**0.60**	**0.90**	**0.68**	**-0.37**

Bold r_s_ values >0.35, *Core Set Measures, Pre (Pre exercise), Post (Post exercise), CEUS (Contrast Enhanced Ultrasound), T_p_ (Time to Peak), I_p_ (Peak Intensity), AUC (Area Under the Curve), CHAQ (Childhood Health Assessment Questionnaire), Pt (Patient), Patient-Reported Outcome Measurement Information System Mobility Short Form (PROMIS-SF), MDAS (Myositis Disease Activity Score), CMAS (Childhood Myositis Assessment Scale), MMT8 (Manual Muscle Testing 8), STS (Sit to Stand), TUG (Time Up and Go), 6MWD (6 Minute Walk Distance).

**Funding**

This study received funding from Cure JM and Children’s Hospital of Pittsburgh Radiology Department.

**Ethics Approval**

University of Pittsburgh Medical Center IRB was obtained and approved for this study following protocol #17020677.

## P53 Patients with pediatric onset systemic lupus erythematosus in South Africa

### Laura B. Lewandowski^1^, Christiaan Scott^2^

#### ^1^Systemic Autoimmunity Branch, National Institute of Arthritis and Musculoskeletal and Skin Diseases, National Institutes of Health, Bethesda, MD, USA; ^2^Paediatric Rheumatology, Red Cross War Memorial Children’s Hospital and University of Cape Town, Cape Town, South Africa

**Background**

Systemic lupus erythematosus (SLE) is a systemic autoimmune disease which can be fatal. While African ancestry is a risk for severe disease and poor outcomes, less is known about SLE on the African continent. Our group recently reported that pediatric onset SLE patients (pSLE) in South Africa(SA) have higher disease activity at presentation and suffer more organ damage than North American counterparts, but was limited to one geographical area. The aim of this study is to describe organ involvement, organ damage, renal survival and overall survival in a larger, more geographically diverse South African cohort.

**Methods**

We conducted a retrospective chart review of pediatric and adult rheumatology and nephrology patients seen at 3 centers in Cape Town and one center in Johannesburg, South Africa from 1988-2017 meeting American College of Rheumatology criteria for pSLE. Patient age, gender, race, presenting features, treatment, and outcomes were recorded. All calculations were performed using R statistical software.

**Results**

The South Africa Cohort includes 170 patients; mean age 11.2 years, 91% female. The larger cohort continues to have high rates of organ system involvement. The current cohort has a 56% rate of renal disease and 35% rate of central nervous system (CNS) SLE. Of those with lupus nephritis, 54% underwent renal biopsy. The majority (60%) of those that underwent biopsy had class III or class IV lupus nephritis. Twenty-two percent of all patients had acute renal failure (ARF) at enrollment, and 9% went on to develop end-stage renal disease (ESRD). The cohort had a high SLE Systemic Lupus International Collaborating Clinics Damage Index (SLICC-DI) score at enrollment.

**Conclusions**

SA patients from a larger, more geographically diverse cohort present at a young age with multisystem involvement. They have high rates of renal and CNS involvement, and a high mortality rate. These differences may be due to access to diagnosis and treatment, genetic predisposition, or a combination of factors. Further research is required to determine identify risk factors for damage and death in this high-risk population.

**Table 1:** **(abstract P53).** Demographic and clinical features at enrollment

	South African Cohort (N=151)
Age at diagnosis, years median (SD)	11.2 (3.2)
% female	91
% African descent	90.5
% ANA positive	97
% lupus nephritis	56
% CNS SLE	35

**Table 2:** **(abstract P53).** Renal features, outcomes, and organ damage

	South African Cohort (N=151)
% renal biopsy	54
Class III/IV	60
Class V	13
% ARF	22
% ESRD	9
% Dialysis	10
% Transplant	4
% Death	9
SLICC-DI median, (SD)	1.537 (1.6)

## P54 Development of the treatment and education approach for childhood-onset lupus (TEACH) intervention for adolescents and young adults

### Natoshia Cunningham^1,2^, Erin Moorman^1^, Lauren Fussner^1^, Pinar Ozge Avar Aydin^1^, Allen Watts^1^, Onengiya Harry^1^, Susmita Kashikar-Zuck^1,2^, Hermine Brunner^1,2^

#### ^1^Cincinnati Children’s Hospital Medical Center, Cincinnati, Ohio, USA; ^2^University of Cincinnati College of Medicine, Cincinnati, Ohio, USA

##### **Correspondence:** Natoshia Cunningham

**Background**

Childhood-onset lupus (cSLE) has been linked to significant impairment in health-related quality of life including increased fatigue, depressive symptoms, and pain. To date, a customized psychological intervention to meet the unique needs of adolescents and young adults (AYA) with cSLE (e.g., adjustment to diagnosis, sleep hygiene) has not been developed. Thus, the current study tested the feasibility and initial effectiveness of a tailored cognitive behavioral treatment for AYA with cSLE (Treatment and Education Approach for Childhood Onset Lupus; TEACH) with the goal of improving fatigue, depressive symptoms, and pain.

**Methods**

TEACH consists of six one-hour individual treatment sessions administered by a psychologist. Separate protocols were developed for adolescents and young adults. Both protocols consist of psychoeducation, cognitive strategies and behavioral skills training adapted for AYA with cSLE. To qualify, patients were required to meet clinical cut offs for fatigue (PROMIS Pediatric Fatigue- short form; cut off: ≥20/40), depressive symptoms (youth < 18 years: Children’s Depression Inventory 2; cut off: ≥14/56; youth ≥ 18 years: Beck Depression Inventory-II; cut off: ≥ 14/63), or pain intensity (Visual Analog Scale; cut off: ≥4/10). All measures were completed before and immediately following treatment. Participants also provided feedback on the content and structure of TEACH via a semi-structured interview.

**Results**

Sixteen patients are enrolled for participation in the study (recruitment is ongoing). Of these, 2 failed to begin and 2 discontinued treatment early. Eleven female participants (mean age 16.4 years) completed the intervention and 1 participant is in treatment. Wilcoxon signed-rank tests indicated that treatment completers <18 years (n = 8) experienced significant reductions in fatigue (M∆=4.37, Z=-2.52, p=<05) and depressive symptoms (M∆=4.29, Z=-2.38, p=<05), but not pain. Treatment completers ≥ 18 years (n = 3) experienced reductions in fatigue (M∆=11.33), depression (M∆=10.00), and pain (M∆=1.50). Participants reported they enjoyed the intervention content and format, and reported increased confidence and self-efficacy.

**Conclusion**

Reductions in fatigue and depressive symptoms were reported among treatment completers. Young adult treatment completers experienced reduction in pain. Participants felt the intervention was beneficial and practical. TEACH may be a feasible and effective method to help AYA manage symptoms related to cSLE.

**Ethics Approval**

The study was approved by Cincinnati Children’s Hospital Medical Center’s Internal Review Board, approval number 2016-0802.

## P55 Living with Childhood-onset Systemic Lupus Erythematosus: A Focus Group with Caregivers

### Onengiya Harry, Angela C. Combs, Brooke Hater, Emily Roemisch, Aimee W. Smith, Lauren M. Fussner, Rhyanne McDade, Leslie Favier, Najla Aljaberi, Allen Watts, Jennifer L. Huggins, Lori E. Crosby, Avani C. Modi

#### Cincinnati Children’s Hospital Medical Center

##### **Correspondence:** Onengiya Harry

**Background**

Non-adherence in childhood-onset systematic lupus erythematosus (cSLE) is estimated to be between 40-50% [1, 2]. In patients with cSLE, non-adherence results in a significant health care burden with increased hospitalizations, preventable disease damage, disease flares, and higher health care costs [1]. There are no published data regarding caregivers’ perspectives on management of cSLE including barriers to treatment adherence for adolescents with cSLE. The aim of this study is to characterize caregivers’ perspective on the management of cSLE and its impact on family life.

**Methods**

Nine caregivers (100% mothers; 67% White; 33% Black) of adolescents diagnosed with cSLE (N=10, 100% female; 70% White; 30% Black; mean age = 15.5± 3.03 years) were recruited from a pediatric rheumatology clinic and its cSLE registry to participate in focus groups. Two group sessions were led by trained facilitators to discuss topics around living with cSLE and its management. The sessions were audio-taped, transcribed, and coded for themes by three independent coders.

**Results**

Preliminary analysis revealed six major themes: 1) Barriers/Facilitators of treatment adherence, 2) Symptoms Impacting Daily Life, 3) Lack of Understanding/Knowledge about cSLE, 4) Need for School Advocacy, 5) Sense of Normalcy/Disruption of Family Schedule, and 6) Worry about the Future. Adherence barriers included the number, taste, timing and side effects of oral medications. Facilitators of adherence were desire to avoid being sick/in pain/hospitalized, use of pill boxes, reminder apps, and storage location of pills. Fatigue, pain, and mood significantly impacted daily life. Caregivers expressed difficulty with balancing adolescents’ autonomy with treatment supervision. All caregivers emphasized need for school advocacy regarding absenteeism due to symptoms and hospitalizations, in-school accommodations, and healthcare providers helping increase schools’ awareness and understanding of cSLE. Worry about the future involved insurance coverage, quality of life, transition to college, and job opportunities.

**Conclusion**

While adherence was identified as a significant problem, caregivers also noted beneficial strategies related to self-management. Caregivers’ desired increased knowledge/understanding from the public regarding cSLE, especially aimed at school advocacy. Important next steps are to identify themes from our adolescent and young adult focus groups, with the long-term goal of developing interventions to improve management of cSLE for adolescents and young adults.

**Ethics Approval**

This study was approved by the Cincinnati Children's Hospital IRB Medical Center board (IRB # 2017-5659).

**References**

1. Feldman, C.H., et al., *Medication Nonadherence Is Associated With Increased Subsequent Acute Care Utilization Among Medicaid Beneficiaries With Systemic Lupus Erythematosus.* Arthritis Care Res (Hoboken), 2015. **67**(12): p. 1712-21.

2. Bugni, V.M., et al., *Factors associated with adherence to treatment in children and adolescents with chronic rheumatic diseases.* J Pediatr (Rio J), 2012. **88**(6): p. 483-8.

## P56 Safety of belimumab in patients with adult- and pediatric-onset neuropsychiatric systemic lupus erythematosus

### Meiqian Ma^1^, Suhas K Ganguli^1^, Barbara Anne Eberhard^1^, Joyce S Hui-Yuen^1*^, Anca Askanase^2*^

#### ^1^Pediatric Rheumatology, Cohen Children’s Medical Center and Hofstra University School of Medicine, NY, USA; ^2^Division of Rheumatology, New York-Presbyterian Hospital/Columbia University Medical Center, NY, USA

**Background**

Belimumab (Benlysta) is a human monoclonal antibody that inhibits soluble B-lymphocyte stimulator. It was approved by the FDA in 2011 for treatment of adults with active, autoantibody-positive, systemic lupus erythematosus (SLE). To date, data are scarce on the use of belimumab in patients with neuropsychiatric lupus (NPSLE).

**Methods**

Retrospective study of SLE patients treated with belimumab who either had NPSLE prior to start of belimumab, or developed NPSLE symptoms while under treatment with belimumab. Data were collected on clinical features, laboratory results, disease course, and treatment response. Descriptive statistics were used where appropriate.

**Results**

We previously reported a cohort of 195 patients treated with belimumab from 10 academic centers. Twenty-one of these patients, from 8 centers, had NPSLE prior to starting belimumab and 5 developed NPSLE while on belimumab. The mean age at start of belimumab was 34.7 (+/- 10.9) years; the median disease duration was 8 years. All 26 patients were female, 9.5% Black, 62% Caucasian, 4.7% Asian, 4.7% Alaskan, and 23% other/unknown. All patients were taking other background medications prior to initiation of belimumab (85.7% hydroxychloroquine, 81% prednisone mean daily dose of 18.8±12.1mg prednisone/equivalent, 19% azathioprine, 52.3% mycophenolate mofetil). Indications for initiation of belimumab included rash, arthritis, renal, hematologic abnormalities, serositis, and worsening serologic activity (low complement and increasing anti-dsDNA). The mean duration of belimumab therapy was 22.7 +/- 13.9 months. Belimumab was discontinued in 5 patients after a mean of 14.3 +/- 15.6 months due to neuropsychiatric symptoms such as headache, stroke-like symptoms, or worsening NPSLE. Of the 5 patients who developed NPSLE symptoms while on belimumab therapy, 3 had pediatric-onset SLE (pSLE, diagnosed before 19^th^ birthday). Symptoms included seizures, stroke, and severe migraines.

Of note, 1 pSLE patient was started on belimumab for peripheral neuropathy and serologic abnormalities. Her clinical symptoms stabilized and her complement and anti-dsDNA levels trended toward normal. In addition, another pSLE patient without NPSLE in the past, developed cognitive impairment on treatment with belimumab and was diagnosed with CNS vasculitis.

**Conclusion**

We report 26 patients who developed or had worsening NPSLE while on treatment with belimumab. This is the first study describing neuropsychiatric outcomes and adverse effects in patients treated with belimumab. Our data are consistent with the observed effects of neuropsychiatric symptoms from the randomized trials, with 8-10% of patients treated with belimumab developing NPSLE symptoms. We are also the first to describe the use of belimumab to treat peripheral neuropathy. Thus, we remain cautious about the use of belimumab in patients with NPSLE. Further studies with larger populations and longer follow up will be beneficial to elucidate the role of belimumab in NPSLE.

**Ethics Approval**

This study was approved by the Columbia University Internal Review Board.

## P57 Cutaneous Scarring in Neonatal Lupus: A Retrospective Cohort Study

### Lauren Briggs^1^, Rebecca Levy^2^, Earl Silverman^2^, Elena Pope^1,2^, Irene Lara-Corrales^1,2^

#### ^1^Faculty of Medicine, University of Toronto, Toronto, ON, Canada; ^2^Hospital for Sick Children, Toronto, ON, Canada

##### **Correspondence:** Lauren Briggs

**Background**

Neonatal Lupus Erythematosus (NLE) is a rare autoimmune condition occurring in newborns secondary to trans-placental passage of maternal autoantibodies during pregnancy. Cutaneous rashes are among the most common manifestations of NLE seen in up to 50% of patients. Lesions are classically described as annular, erythematous plaques favoring periorbital and photo-distributed sites. Traditionally thought to be self-resolving, some literature suggests that cutaneous sequelae may persist in a subset of patients.

**Objective**

To explore the clinical features of cutaneous NLE, and characterize the presence of cutaneous sequelae, including atrophic scarring.

**Methods**

A retrospective cohort study of pediatric patients diagnosed with cutaneous NLE, and born between January 1980 and May 2017, was carried out at a tertiary pediatric referral center. 106 patients met inclusion criteria. All patients in the study were at least 6 months of age at last follow-up. Data were summarized using descriptive statistics; a regression analysis was used to explore risk factors for atrophy. Statistical analysis was performed using STATA 14.0, Texas, US.

**Results**

57% of patients were female. Known maternal connective tissue disease was noted in 65% of cases. The antibodies most frequently present included anti-Ro, anti-La with frequencies of 93% and 68% respectively. The rash was most often described as papulosquamous (72%) and annular (71%). Sites of involvement included head and neck only (39%), body only (8%), or widespread (54%). At last follow up, 34% of patients had cutaneous sequelae from the NLE rash. Of these, 13% of patients had residual telangiectasia, 17% had dyspigmentation and 9% had atrophic scarring. Mean duration of follow-up was 3.8 years +/- 4.3 (0.2-18.5), and mean age at last follow-up was 4 +/- 4.3 (0.5-18.7). Atrophic scarring was significantly associated with both maternal anti-Ro antibodies (Pearson Chi2 9.7, P=0.0002) and topical treatment of NLE lesions (Pearon Chi2 5.5, P=0.02).

**Conclusion**

The results of this study suggest that NLE can result in cutaneous sequelae, including atrophic scarring, telangiectasia, hyperpigmentation and hypopigmentation. Anti-Ro antibodies may signify increased propensity of cutaneous scarring. This suggests the importance of ensuring accurate diagnosis of cutaneous NLE, in conjunction with long-term monitoring.

**Ethics Approval**

This study was approved by the Hospital for Sick Children’s Internal Review Board, approval number 1000055474.

## P58 Childhood primary angiitis of the central nervous system: deciphering a rare disease through international collaboration

### Marinka Twilt^1^, Shehla Sheikh^2^, Courtney Moore^2^, Anastasia Dropol^1^, Ana Sepulveda^1^, Susanne Benseler^1^, on behalf of the BrainWorks investigators

#### ^1^Alberta Children’s Hospital, University of Calgary, Calgary, Alberta, Canada; ^2^Sickkids, Toronto, Ontario, Canada

##### **Correspondence:** Marinka Twilt

**Introduction**

Childhood Primary Angiitis of the central nervous system (cPACNS) is an increasingly recognized inflammatory brain disease with potentially devastating neurological deficits. Early recognition and initiation of treatment has led to dramatically improved mortality and decreased morbidity. Immunosuppressive treatment is tailored to the disease subtype. Three distinct subtypes have been recognized and are based on the size of the brain vessel involved. Large vessel cPACNS, angiography positive, includes two subtypes; progessive (P-cPACNS) and non-progressive cPACNS (NP-cPANCS). Small vessel, angiography negative cPACNS (AN-cPACNS) is confirmed by brain biopsy. BrainWorks is an international registry for inflammatory brain diseases, endorsed by CARRA initiated to collect these rare cases. The aim of this study is to describe the presentation, course and outcome of children with cPACNS in BrainWorks.

**Methods**

Consecutive patients with inflammatory brain diseases age < 18 years at the time of diagnosis were prospectively enrolled in the BrainWorks study since 2007. BrainWorks is a multicenter collaborative cohort study of all subtypes of inflammatory brain diseases. Children were included if they had a confirmed diagnoses of P-cPACNS, NP-cPACNS, or AN-cPACNS according to modified Calabrese criteria. Demographics, clinical features, inflammatory markers, and standardized functional assessments were extracted from BrainWorks. Informed consent was obtained, following approval from the respective Research Ethics Boards.

**Results**

To date, a total of 445 patients have been enrolled in BrainWorks. Of these, 257 patients (58%) were diagnosed with cPACNS. Of these, large vessel vasculitis was diagnosed in 158 patients (61%) including NP-cPACNS in 121 and P-cPACNS in 37. Small vessel cPACNS was diagnosed in 99 (39%). more Children with large vessel disease more commonly presented with hemiparesis (NP-cPACNS: 105/121, 87%, P-cPACNS: 20/37, 54% versus AN-cPACNS: 36/99, 36%) and speech impairment (NP-cPACNS: 66/121, 54%, P-cPACNS: 17/37, 46% versus AN-cPACNS: 39/99, 39%). Seizures (general, focal and status) were seen most frequently in the AN-cPACNS: 85/99, compared to large vessel vasculitis (25/121 NP-cPACNS: 21%, P-cPACNS:24%). Interstingly, behavioral changes and cognitive impairment were seen most frequently in AN-cPACNS (38/99, 38% and 41/99, 41%) and P-cPACNS patients (8/37, 22% and 12/37, 32%). The Pediatric Stroke Outcome Score was available for 191 patients (P-cPACNS: 22, NP-cPACNS: 96, AN-cPACNS: 73) and the average score was 1.25 (P-cPACNS: 1.6 , NP-cPACN: 0.9, AN-cPACNS: 1.75), the highest and therefore the largest functional burden was documented in children with small vessel vasculitis.

**Conclusion**

The BrainWorks study determined the clinical phenotypes and outcomes of children with cPACNS: the large vessel cohort presented primarily with hemiparesis and speech impairment, while small vessel CNS vasculitis results in seizures, cognitive dysfunction and behavioral abnormalities. At the one-year-follow-up, children with cPACNS demonstrated a moderate functional impairment, most significantly in the AN- and P-cPACNS cohorts.

## P59 Descriptive analysis of pediatric patients with systemic vasculitis admitted to the intensive care unit of a tertiary care hospital: 10-year experience

### Ellen Go^1^, Alicia Teagarden^2^, Courtney M. Rowan^2^, Stacey Tarvin^1^

#### ^1^Division of Pediatric Rheumatology, Indiana University School of Medicine, Riley Hospital for Children, Indianapolis, IN, USA; ^2^Division of Critical Care, Indiana University School of Medicine, Riley Hospital for Children, Indianapolis, IN, USA

##### **Correspondence:** Ellen Go

**Background**

Primary systemic vasculitis (PSV) is a heterogeneous group of disease with diverse clinical symptoms. The non-specific clinical manifestation frequently leads to a delay disease diagnosis. Therefore, patients often present with complications such as severe hypertension, heart failure, kidney injury or a neurological event requiring intensive care unit admission. We aim to describe patient characteristics and interventions required for patients with PSV admitted to a tertiary care hospital pediatric intensive care unit (PICU).

**Methods**

A retrospective cohort study of all patients diagnosed with PSV who were referred to pediatric rheumatology at Riley Hospital for Children from January 2007 to December 2017 was conducted. We investigated PICU admission, clinical characteristics, and critical care intervention.

**Results**

142 PSV patients were seen by pediatric rheumatology at Riley Hospital for Children. Henoch-Schonlein purpura (HSP) (n=100, 70.4%) was the most common PSV diagnosis, followed by refractory Kawasaki disease (KD) (n=17, 12%), anti-nuclear cytoplasmic antibody (ANCA)-associated vasculitis (n=12, 8.5%), Takayasu’s arteritis (n=8, 5.6%), polyarteritis nodosa (n=3, 2.1%) and primary central nervous system (CNS) vasculitis (n=2, 1.4%). There were 18 patients (12%) with PSV and who required PICU admission (Table 1). Highest rate of PICU admissions were Takayasu’s arteritis (62.5%) and primary CNS vasculitis (50%). For critical care characteristics (Table 2), rule out sepsis was the leading cause of admission and more than half required respiratory support with at least a third received vasoactive agents. No mortality seen in PICU PSV cohort.

**Conclusions**

This is one of the first descriptive cohort of pediatric patients with PSV requiring critical care. Generally patients with HSP and refractory KD are unlikely to have severe enough disease to require specific PICU intervention. While HSP is the most frequent, the PICU admission rate is highest in chronic systemic vasculitides like Takayasu arteritis. Further diagnosis subgroup data analysis is ongoing.

**Ethics Approval**

This study was approved by Indiana University’s Institutional Review Board, approval number 1711175931.

**Table 1:** **(abstract P59).** Patient Characteristics

	PICU PSV Cohort (n=18)
Median age, years (IQR)	9.5 (5.9-13.7)
Male/Female, n (%)	7 (38.9)/11 (61.1)
Diagnosis, n (%)	
Refractory Kawasaki disease	6 (33.3)
Takayasu’s arteritis	5 (27.8)
ANCA-associated vasculitis	4 (22.2)
Henoch-Schonlein purpura	2 (11.1)
Primary CNS Vasculitis	1 (5.6)
Others	0 (0)
Diagnosis during hospital admission, n (%)	15 (83.3)
Treatment during hospitalization	
Glucocorticoids	15 (83.3)
IVIg	7 (38.9)
Aspirin	5 (27.8)
Cyclophosphamide	4 (22.2)
Disease modifying anti-rheumatic drugs	2 (11.2)
Biologics	1 (5.6)
Median hospital length of stay, days (IQR)	8.5 (4.0-18.8)

**Table 2:** **(abstract P59)**. Characteristics of PICU Stay

Reason for PICU admission, n (%)	
Rule out sepsis	6 (33.3)
Hypertension	2 (11.1)
GI bleeding	2 (11.1)
Others	8 (44.5)
PICU interventions, n (%)	
Vasoactive agent	7 (38.9)
Invasive ventilation	6 (33.3)
High flow nasal cannula	4 (22.2)
Non-invasive ventilation	2 (11.1)
Dialysis	1 (5.6)
Plasmapheresis	1 (5.6)
Median PICU length of stay, n (%)	
0-2 days	9 (50)
3-7 days	5 (27.8)
>7 days	4 (22.2)
PICU Mortality, n (%)	0 (0)

Abbreviations: ANCA=anti-neutrophil cytoplasmic antibody; CNS=central nervous system; GI = gastrointestinal; IVIg=intravenous immune globulin; PICU= pediatric intensive care unit

## P60

This abstract is not included here as it has already been published.

## P61 Prevalence of Psychiatric Disorders in a Transition Clinic of Adolescents with Rheumatic Diseases in México

### Nadina E. Rubio-Pérez^1^, Ana C. Arana-Guajardo^2^, Marcia D. Torres Made^1^, Fernando García-Rodríguez^1^, Ana V. Villarreal-Treviño^1^, Antonio López-Rangel^3^, María E. Corral-Trujillo^4^, Dionicio A. Galarza-Delgado^2^

#### ^1^Pediatric and; ^2^Adult Rheumatology Services; ^3^Psychiatry Department; and ^4^Clinical Psychologist. Autónoma de Nuevo León University. Hospital Universitario "Dr. José Eleuterio González", Monterrey, Mexico

**Background**

The adolescence is a critical period when different process are made, as identity, cognitive changes, enhance of autonomy, and emotional, physical and sexual development. Besides that, an adolescent with rheumatic disease has to front facing the disease acceptance. The recognition of psychiatric disorders is of utmost importance in order to achieve a healthy transition to adult-centered health care services.

**Objective**

To describe prevalence of psychiatric disorders in adolescents who attended our Transition Clinic.

**Methods**

We included rheumatologists, psychiatrist, clinical psychologist, nurse and social services. We included patients with established rheumatic diseases older than 16 years old, who attended the Transition Clinic in the period between July and December 2017. We used MINI KID assessment tool to characterized psychiatric disorders in our patients. Each patient performs an interview with both a clinical psychologist and a pediatric psychiatrist to confirm diagnosis. We use descriptive statistics with measures of central tendency or frequencies depending on variable characteristics.

**Results**

Nineteen patients had already seen at the clinic, most of them are female (79%) and with mean age of 18 years old. The most frequent diagnosis were juvenile idiopathic arthritis (48%) and systemic lupus rythematosus (26%).

After a screening assessment with MINI KID tool, 14 (74%) patients shown an abnormal result. Those patients were later evaluated finding agoraphobia (21%), depression (26%), specific phobias (21%) and social anxiety disorder (26%) as the most prevalent diseases (Table 1 and Figure 1).

**Conclusion**

Transition from pediatric to adult health-care is a transcendental step in life, especially when a chronic disease is present. In this pilot study, we shown a high prevalence of psychiatric problems among adolescents.

These results encourage the need of an organized, specialized, multidisciplinary, and integrated clinic in which the patient could adapt to adult care centers.

**References**

1. Oen K. Comparative epidemiology of rheumatic diseases in children. Curr Opin Rheumatol 2000;12:410.

**Table 1:** **(abstract P61).** Psychiatric diagnosis in the patients using MINI KID tool. Each patient could have more than one diagnosis

Diagnosis	Number of patients	%
Agoraphobia	4	21
Anxiety	1	5
Separation anxiety disorder	3	16
Depression	5	26
Mild Intellectual Disability	1	5
Dysthymia	2	11
Specific Phobias	4	21
Social Anxiety Disorder	5	26
Oppositional defiant disorder	1	5
Obsessive-Compulsive Disorder	2	11
Past suicide risk	1	5
Attention-deficit/hyperactivity disorder	1	5

**Fig. 1:**
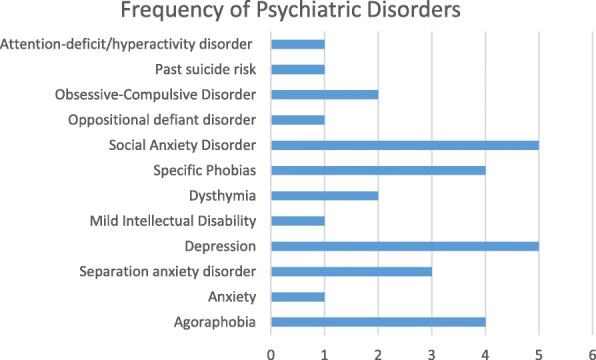
**(abstract P61).** Psychiatric diagnosis in the patients using MINI KID tool. Each patient could have more than one diagnosis

## P60 Leveraging a patient-powered research network to understand the impact of adverse childhood experiences and resilience on children with rheumatic diseases

### Danielle R. Bullock^1^, Kaveh Ardalan^2^, Tamar Rubinstein^3^

#### ^1^University of Minnesota/Masonic Children’s Hospital, Minneapolis, MN, USA; ^2^Northwestern University Feinberg School of Medicine/Ann & Robert H. Lurie Children’s Hospital of Chicago, Chicago, IL, USA; ^3^Albert Einstein College of Medicine/Children’s Hospital at Montefiore, Bronx, NY, USA

##### **Correspondence:** Danielle R. Bullock

**Background**

Adverse Childhood Experiences (ACEs), such as violence, abuse, neglect, scarcity, and household dysfunction, are associated with chronic disease and worse health. Studies in children suggest that resilience, defined as the ability to recover from adversity, can mitigate health effects of ACEs. While an association between ACEs and autoimmune diseases in adults has been described, a relationship between ACEs and childhood-onset rheumatic diseases has not yet been examined in detail. We performed preliminary explorations of the relationships between arthritis, ACEs, and resilience using data from the 2016 National Survey of Children’s Health (NSCH). Further prospective data is needed in order to better understand the relationships between ACEs, resilience, and childhood-onset rheumatic diseases. Our objectives are: 1) to engage the CARRA community and stakeholders in eliciting feedback on a project studying ACEs and resilience in youth with rheumatic diseases; 2) to assess the acceptability among patients and parents of collecting data on ACEs and resilience during CARRA Registry visits at selected sites.

**Methods**

We propose a project leveraging the Patients, Advocates and Rheumatology Teams Network for Research and Service (PARTNERS) consortium to engage children with rheumatic diseases and their parents in the design of a survey to collect preliminary data on ACEs, resilience, patient-reported outcomes in juvenile idiopathic arthritis, systemic lupus erythematosus, and juvenile dermatomyositis populations. We also propose collecting data on the acceptability of administering these measures during CARRA Registry visits to families of patients enrolled in the CARRA Registry. Because assessing ACEs can involve sensitive topics and has implications for patient and family safety, it is important to represent patient/family voices in the design of this study.

**Results**

Knowledge gained from this project will improve our understanding of a relationship between ACEs, resilience, and patient-reported outcomes in childhood-onset rheumatic diseases and will help determine whether collecting data on ACEs and resilience will be acceptable to families participating in the CARRA Registry. Future projects could include a pilot study of CARRA Registry participants to assess the feasibility of collecting this data, the rate of missingness, and perceptions of the usefulness of collecting such data more broadly for all registry patients in future iterations of the CARRA Registry.

**Conclusions**

ACEs and resilience may help explain differential outcomes among patients with pediatric rheumatic diseases. A greater understanding of these factors can guide the development and testing of novel resilience-building interventions for improving clinical and patient-reported outcomes. Engaging families in the design of studies of ACEs and resilience will help ensure that data is collected in a manner that is acceptable to patients and parents.

**Ethics Approval**

Prior to initiation of any data collection, the necessary IRB approval will be obtained.

## P63 Getting a HEEADSSS in psychosocial screening: use of standardized clinic note templates for psychosocial screening in a pediatric rheumatology clinic

### Erin Brennan Treemarcki, Jackie Szymonifka, Alexa Adams, Nancy Pan, Sarah Taber, Karen Onel

#### Hospital for Special Surgery, New York, New York, USA

##### **Correspondence:** Erin Brennan Treemarcki

**Background**

Children and adolescents with rheumatic diseases are at risk for psychosocial challenges related to illness, treatment, and normal development and should receive psychosocial screening. Psychosocial assessments have been successfully utilized in other pediatric specialties, including oncology and gastroenterology. A common psychosocial screening tool is the HEEADSSS (home environment, education/employment, eating, activities, drugs, sexuality, suicide/depression, and safety). This tool is easy to remember, quickly administered, and modifiable for younger children. We conducted a quality improvement project aimed to increase psychosocial screening by providers in a single pediatric rheumatology clinic by instituting standardized follow up templates for juvenile idiopathic arthritis (JIA) and systemic lupus erythematosus (SLE) including the HEEADSSS assessment.

**Methods**

The quality improvement intervention evaluated in this study involved the creation of disease specific follow up note templates inclusive of the HEEADSSS assessment and introduction of the templates to the pediatric rheumatology fellows’ clinic. Charts of patients with an ICD-10 diagnosis of JIA or SLE were reviewed during a 3 month window prior to (February-April 2017) and after (October-December 2017) the intervention. Charts were reviewed for presence of 2 elements of the HEEADSSS assessment documented within the previous 12 months: smoking exposure (all patients) and sexual activity (ages 11 years and up). The proportion of patients with completed HEEADSSS assessment components pre- and post-intervention were compared using Fisher’s exact test.

**Results**

Chart review included 36 patients pre- and 42 post-intervention (Table 1). There was an increase in assessment of both measures in the period immediately following the intervention. Assessment of smoking exposure increased from 0.0% to 21.4% (p=0.003) with greater but not significant improvement in SLE versus JIA patients (29.4% versus 16.4%, p=0.45). Assessment of sexual activity in patients 11 years and older increased from 13.0% to 37.5% (p=0.09), although this comparison may be underpowered due to decreased sample sizes.

**Conclusion**

Introduction of standardized note templates with the HEEADSSS assessment has resulted in preliminary improvement in psychosocial screening by providers in a single pediatric rheumatology clinic as demonstrated by improved rates of screening for smoking exposure and a trend towards improved screening rates for sexual activity. Additional studies are needed to confirm these findings, and future studies will assess whether the improvement seen is sustained and whether usage of this template can be expanded to other pediatric patient populations.

**Table 1:** **(abstract P63).** Documentation status pre- and post- implementation of standardized follow up templates for providers

	Pre-Intervention	Post-Intervention	p-value
Smoking assessed			
Overall	0/36 (0.0%)	9/42 (21.4%)	0.003
JIA	0/20 (0.0%)	4/25 (16.0%)	0.12
SLE	0/16 (0.0%)	5/17 (29.4%)	0.04
Sexual Activity (11+) assessed			
Overall	3/23 (13.0%)	9/24 (37.5%)	0.09
JIA	1/9 (11.1%)	2/8 (25.0%)	1.00
SLE	2/14 (14.3%)	7/16 (43.8%)	0.14

Abbreviations: JIA=juvenile idiopathic arthritis; SLE=systemic lupus erythematosus

## P64 A collaborative approach to diagnosis and treatment of hematophagocytic lymphohstiocytosis and macrophage activation syndrome: a single center’s approach to the development of evidence-based guidelines

### Olha Halyabar^1,2^, Margaret H. Chang^1,2^, Michelle Schoettler^1,3^, Marc A. Schwartz^1,3^, Leslie Benson^1^, Catherine M. Biggs^4^, Fatma Dedeoglu^1^, Mark Gorman^1^, Leslie Lehmann^1,3^, Mindy S. Lo^1^, Peter A. Nigrovic^1,2^, Craig D. Platt^1^, Gregory P. Priebe^1^, Jared Rowe^1,3^, Robert P. Sundel^1^, Neeraj K. Surana^1^, Katja Weinacht^5^, Barbara A. Degar^1,3^, Melissa M. Hazen^1^*, Lauren A. Henderson^1*^

#### ^1^Boston Children’s Hospital and Harvard Medical School, Boston, MA, USA; ^2^Brigham and Women’s Hospital and Harvard Medical School, Boston, MA, USA; ^3^Dana-Farber Cancer Research Institute and Harvard Medical School, Boston, MA, USA; ^4^British Columbia Children’s Hospital, Vancouver, BC, Canada; ^5^Lucile Packard Children’s Hospital Stanford, 300 Pasteur Drive, Stanford, CA, USA

##### **Correspondence:** Olha Halyabar

**Background**

Historically, primary hemophagocytic lymphohistiocytosis (HLH) and macrophage activation syndrome (MAS) were thought of as distinct diseases and management was divided between hematology/oncology and rheumatology with little communication between the two specialties. It is now evident that the underlying pathophysiology of these entities is closely related, underscoring the need to develop a multidisciplinary approach to HLH/MAS. We utilized quality improvement methods to develop consensus on the diagnosis and treatment of patients with HLH/MAS at our hospital.

**Methods**

For support, we leveraged the Evidence Based Guideline (EBG) Initiative, which is administered through the Quality Improvement Program of the Department of Medicine. An EBG is a clinical algorithm constructed from available evidence in the literature and expert opinion of locally involved clinicians. The guideline is created through prescribed steps that are designed to build consensus and ensure successful implementation. Clinical outcomes are measured and evaluated to allow for improvement and additional iterations of the EBG.

As per the EBG protocol, we gathered a multidisciplinary workgroup of specialists with expertise in HLH/MAS. A series of face-to-face meetings was held monthly from March 2016 to December 2017 and the meeting format was based on nominal group techniques. The goals of the group included: 1) increased communication across specialties; 2) development of a diagnostic and treatment algorithm for HLH/MAS based on evidence in the literature and expert opinion; 3) establishment of a clinical response team. Once the diagnostic and therapeutic pathway for HLH/MAS was developed, the EBG was presented to the wider hospital community and revisions were made based on feedback. Quality metrics were selected to monitor outcomes and the EBG was implemented.

**Results**

Entry criteria for the EBG, fever and ferritin ≥ 500 ng/mL in a hospitalized patient, is broad and designed to capture as many patients with HLH/MAS as possible. The rheumatology consult team serves as the initial point of contact if HLH/MAS is suspected, and performs patient triage and provides recommendations for additional laboratory testing and consultations. The rest of the core HLH/MAS team is involved through emails sent to HLH/MAS distribution list, which serves as an open platform for patient discussion. An HLH/MAS order set is available to facilitate the recommended diagnostic work up and treatment guidelines. The treatment algorithm includes suggested medications and doses based on the acuity of illness and risk of concurrent infection (Fig 2). Quality metrics to be tracked prospectively and compared to the 2 years prior to EBG implementation include, time to initiation of treatment, fever duration, length of hospital stay, time to normalization of laboratory markers, and mortality.

**Conclusion**

HLH and MAS are increasingly considered to be a spectrum of related conditions and joint management across subspecialties will likely improve patient outcomes. Our experience in creating a multidisciplinary approach to HLH/MAS management based on consensus can serve as a model at other institutions.

## P65 The role of food in the self-management of pain in teens with juvenile arthritis: gender and ethnic differences

### Kimberly A. Lewis^1,2,3^, Amanda Mabry-Flynn^3^, Ruy Carrasco^4^, Lauren Nielson^2^

#### ^1^The University of Texas at Austin, Austin, TX, USA; ^2^Ascension Texas, Nursing Research, Austin, TX, USA; ^3^The University of Illinois at Urbana-Champaign, Urbana, IL, USA; ^4^Dell Children’s Medical Center, ‘Specially for Children Pediatric Rheumatology Clinic, Austin, TX, USA

##### **Correspondence:** Kimberly A. Lewis

**Background**

The complex emotional interplay between chronic pain and familial and cultural norms about food could lead to the development of disordered eating and obesity in Juvenile Arthritis (JA) patients. JA is a collection of inflammatory joint diseases with symptom onset prior to age 16 that accounts for over $285 mil in annual healthcare costs. Joint pain leads to stiffness, loss of fine motor skills, immobility, or fatigue, and requires daily self-management. Weight gain can result from a sedentary lifestyle or increased appetite and fluid retention from immunosuppressant medications. The negative physical and psychological consequences (i.e. low self-esteem and bullying) may present a challenge for adolescents at a developmental stage in which the pressure to maintain a particular body image is communicated at all levels, from peers to mass media, and is more likely to be internalized resulting in unhealthy behaviors and distorted self-image.

The purpose of this study is to explore the meanings adolescents diagnosed with JA place on food choice in the context of pain, and how messages teens receive about food from interpersonal to mass mediated communication may influence their nutritional self-care beliefs and practices.

**Methods**

The cross-sectional, inductive qualitative study was guided by the social-ecological model. Diverse focus groups of teens aged 13-17 (total sample *N*=46) were recruited from a clinic in Austin, Texas, using a two (Hispanic ethnicity, white) by two (male or female) array. An experienced moderator led the discussion using a semi-structured interview guide. The recorded transcripts were systematically analyzed following an established protocol from previous literature.

**Results**

The preliminary sample from three focus groups (n=9) was 56% female, 33% Hispanic ethnicity, and 22% overweight or obese (body mass index percentile >85). All participants described the multiple levels of interplay between food and pain: individual, familial, community, and societal. Communication at multiple levels (relational, cultural, and mass-media) was a source of influence in all groups for varying reasons. Food choices centered on individual, familial, and peer preferences and beliefs about how the food would make them feel. Hispanic males and females were more likely to mention a spiritual component; consume different foods; and minimize symptoms in order to avoid distressing family members. Peer relationships and societal influences (school, commercials, social media) both positively and negatively affected food choices and pain.

**Conclusion**

Findings from this study indicate similarities and differences based on gender and ethnicity in a sample of adolescents with JA when they communicate about food and pain. These findings have implications for clinical practice to address the dietary needs of this population in a developmentally appropriate, gender-specific, and culturally sensitive way. The communication influence at multiple levels (relational, cultural, and mass-media) is a novel consideration in designing interventions and policy recommendations to promote the best outcomes. Further research is warranted to confirm these findings.

**Ethics Approval**

This study received Institutional Review Board Approval (CR-15-036) from the Seton Institutional Review Board in Austin, Texas prior to enrollment and research activities. All participants and parents provided informed assent and consent prior to participating in the research activities.

**Acknowledgements**

The authors would like to acknowledge Dr. Tracie Harrison, PhD, RN, FAAN, for her role as faculty sponsor for the project. The study was funded by a grant from the Health Communications Scholars Program through the Moody College of Communication, The University of Texas at Austin, Austin, Texas, USA.

## P66 Increasing adult rheumatology fellows’ confidence and competence in providing transition care to transferring young adult rheumatology patients

### Rebecca E. Sadun, Gary R. Maslow, Richard J. Chung, Lisa G. Criscione-Schreiber

#### Duke University, Durham, NC, USA

##### **Correspondence:** Rebecca E. Sadun

**Background**

The transition from pediatric to adult healthcare is a vulnerable time for adolescents and young adults (AYA) with chronic conditions. EULAR and the Paediatric Rheumatology European Society (PReS) jointly published expert opinions regarding transition care of AYA with juvenile-onset rheumatic diseases, and the ACR recently developed a transition toolkit. However, there are no published curricula for teaching transition guidelines, skills, or utilization of existing tools. We therefore designed and evaluated a workshop to help adult rheumatology fellows learn key skills for providing effective transition care to transferring young adult patients.

**Methods**

A 1-hour skills-based workshop on transition and transfer best practices was developed alongside an objective standardized clinical examination (OSCE) station in which trainees welcomed a young adult with lupus – and her parent – to a first visit in an adult clinic. Adult rheumatology fellows (n=19) from 5 institutions were asked to self-asses their ability to perform 10 transition/transfer skills pre- and post-workshop on a Likert scale from 1-4, with 1 being “not at all prepared” and 5 being “completely prepared.” The OSCE evaluation rubric assessed 5 transition/transfer skills on a Likert scale of 1-5, with 5 being the best performance. Twelve fellows were tested with the OSCE de novo, whereas 7 were tested with the OSCE after participating in the workshop. Aggregated pre- and post-workshop survey responses were compared using Fisher’s exact test, and OSCE scores were compared using an unpaired *t*-test. To assess skill retention, second year fellows will be re-tested with the OSCE transfer station twelve months after the initial workshop.

**Results**

After participating in the workshop, fellows felt more confident in their ability to perform 8 of the 10 transition/transfer skills (Table 1). In addition, OSCE performance (Table 2) was significantly better among the fellows who participated in the OSCE after the workshop than among those who took the OSCE de novo. Twelve-month-post-workshop data, establishing the degree of skill retention, will be collected January 2018 and will be analyzed in time for presentation at the 2018 CARRA Meeting.

**Conclusion**

This brief educational intervention successfully increased adult rheumatology fellows’ confidence with many transition/transfer skills as well as increasing fellows’ ability to employ transition best practices in an OSCE setting. It will be important to measure skill retention 1 year following the workshop to ensure long-term impact of the curriculum. Next steps include the development of a complementary workshop for pediatric rheumatology fellows; this workshop will focus on skills that are key to helping young adult rheumatology patients prepare for the transfer to adult care.

**Table 1:** **(abstract P66).** Impact of workshop on fellows’ confidence in ten transition and transfer skills

Self-Assessed Preparedness Increased	Self-Assessed Preparedness Not Increased
Orient young adult to adult rheumatology care (p<0.01)	Establish rapport and trust with young adult patients (p=0.13)
Provide expectations of the young adult patient (p<0.01)	Speak w/ pediatric providers re transferring patients (p=0.07)
Explain differences between pediatric & adult care (p<0.01)	
Assess young adult self-management skills (p<0.0001)	
Assure young adult of confidentiality (p<0.001)	
Ask parent to leave the room for social history (p=0.01)	
Take a transition-focused adolescent social history (p<0.01)	
Identify barriers to transition and adherence (p<0.05)	

**Table 2:** **(abstract P66).** Impact of workshop on fellows’ competence in 5 core transition and transfer skills

	Explaining differences between pediatric and adult care	Placing the AYA patient in the primary role (parent for corroboration)	Assessing self-management skills	Performing a confidential adolescent social history	Assessing barriers to transition & adherence	Total score / Average score
Pre-workshop (n=12)	3.5	4.3	3.8	2.8	2.3	16.7/3.3
Post-workshop (n=7)	4.6	5.0	4.4	4.7	2.6	21.3/4.3
p-value	<0.01	<0.05	0.18	0.01	0.86	0.01

**Ethics Approval**

This study received exemption from the Duke University Internal Review Board.

## P67 The value of ethnography in understanding burden of disease and designing better care delivery systems in pediatric care

### David C. McDonald^1^, Matt Bradley^1^, Brian Shakley^1^, Hermine Brunner^2^, Teresa A. Simon^3^

#### ^1^LIFT 1428, Chattanooga, TN, USA; ^2^Division of Rheumatology, Cincinnati Children’s Hospital Medical Center, Cincinnati, OH, USA; ^3^Bristol-Myers Squibb, Princeton, NJ, USA

##### **Correspondence:** David C. McDonald

**Background**

Qualitative measures of care delivery and disease burden include patient (pt)-reported measures of satisfaction; post-care experience; and social, physical, and mental functioning. Parents’ and patients (pts)’ emotional distress derived from family systems, cultural beliefs, or other unique situations may not be captured using traditional qualitative methods. The purpose of this study is to evaluate the impact of ethnography in understanding burden of disease.

**Methods**

Interviews (INTs) are conducted by a healthcare ethnographer. Ethnographers use a formal INT guide (IG) with parents/pts to better understand their lived experience and clinical environment experiences. INTs are audio- and video-recorded, and audio recordings are transcribed verbatim. A code book is developed for each study; transcripts are analyzed and coded to identify common themes related to guiding the research question and the key topics we investigated (e.g. lived experience, individual/cultural belief systems and pt/parent journey).

This method has been used in several pediatric settings. We describe 2 here: a study of pediatric urgent care delivery environments (A) and a study of juvenile idiopathic arthritis (JIA)pts (B, ongoing). In A, a bilingual semi-structured IG was used for exit INTs with parents within clinical point of care. Post-exit INT, participants could continue being observed and interviewed by an ethnographer (lived [in-home] environment). In B, JIA pts and their parents are being observed and interviewed (lived environment) by an ethnographer using an IG designed to understand individual pt beliefs/behavioral drivers related to the realities of transitioning from a pediatric care environment to an adult care environment, measure impact of beliefs/behavioral drivers on the individual/family via pt-reported experiences, and leverage learning to implement self-management tools into care delivery environments.

**Results**

In A: 38 unique INTs were conducted among 19 clinical staff members (intake staff, healthcare providers) and 19 parents (parents/grandparents); 162 hours were spent observing/interviewing participants in clinical and/or lived environment(s). Four major themes emerged: healthcare is a layered emotional journey for parents/pts and clinicians; there are many opportunities to provide a more meaningful experience; parents/pts can be difficult to manage; and communication creates connection. We also uncovered that the parent paradigm is informed along 4 categories among the 4 themes (Table 1). For B, a sample of 5 pt/parent pairs will be interviewed; preliminary results are planned at time of presentation.

**Conclusion**

There are innate challenges in providing meaningful qualitative insights to help frame a better care delivery experience and improve the current care delivery model. The integration of ethnography, an important tool to gain meaningful insights into the human condition, into patient care may improve the accuracy and comprehensiveness of burden of disease measurements and improve healthcare transactions.

**Table 1:** **(abstract P67).** Parent Perspectives on Patient-Reported Measures of Lived Experience and Burden of Disease

Category	Issues
Sensory overload throughout the journey	▪ Fitting event into activity of daily living ▪ Difficulty suppressing anticipatory anxiety ▪ Difficulty managing logistics ▪ Situation can be psychologically distressing
Multiple determinants of anxiety	▪ Severity of the child’s injury/illness ▪ Day-to-day life circumstance ▪ Mood ▪ Experience level ▪ Family and work–life dynamics
General feeling of being overwhelmed	▪ The perception/belief that ”life is not fair” ▪ The reality that life is a battle ▪ Multiple healthcare challenges in same family unit
Sensitivities to cultural barriers can lead to a positive or negative experience	▪ Socioeconomic circumstances ▪ Language barriers ▪ Cultural/ideological values ▪ Healthcare providers may generalize experiences based on spoken language

**Trial Registration**

Not applicable.

**Ethics Approval**

Study A was performed for a healthcare system and all participants were consented at the time of the study. We have received the appropriate IRB approval for the research involved in this submission for Study B.

